# Metabolomics integrated with network pharmacology and serum-urine pharmacochemistry unveils the antidiabetic mechanism of Anemarrhenae Rhizoma

**DOI:** 10.3389/fendo.2025.1618584

**Published:** 2025-10-09

**Authors:** Xunlong Zhong, Huaidong Peng, Chang Xiao, Chunhua Xiao, Xinyu Zhu, Haixuan Liang, Ruolun Wang, Yanmei Zhong, Jingwen Feng

**Affiliations:** ^1^ Department of Pharmacy, The Second Affiliated Hospital of Guangzhou Medical University, Guangzhou, China; ^2^ Centre for Drug Research and Development, Guangdong Pharmaceutical University, Guangzhou, China; ^3^ Department of Pharmacy, Panyu Hospital of Traditional Chinese Medicine, Guangzhou, China

**Keywords:** *Anemarrhenae Rhizoma*, metabolomics, network pharmacology, pharmacochemistry, antidiabetic mechanism

## Abstract

**Objective:**

Anemarrhenae Rhizoma (AR) is a traditional Chinese medicine widely used for the treatment of type 2 diabetes mellitus (T2DM). However, the specific bioactive constituents responsible for its *in vivo* effects and their underlying mechanisms of action remain unclear. We hypothesise that serum-absorbed and metabolised AR components modulate key metabolic and inflammatory pathways in T2DM. To test this hypothesis, this study employs an integrated strategy combining metabolomics with serum-urine pharmacochemistry and network pharmacology to systematically identify AR’s active constituents and elucidate their multi-target mechanisms in T2DM management.

**Methods:**

UHPLC-Q-TOF-MS coupled with multivariate statistical analysis was employed to identify the AR-derived constituents in serum and urine of T2DM rats. Network pharmacology was utilised to predict the targets of the AR’s active components, while biochemical assays, liver histopathology, and metabolomics were performed to evaluate its therapeutic effects. Molecular docking and molecular dynamics (MD) simulations were conducted to assess the binding affinities between key components and their targets.

**Results:**

77 AR components were identified, among which 47 prototypes and 11 metabolites were detected in serum and urine. The key bioactive constituents included sarsasapogenin, markogenin/neogitogenin, digitogenin, norathyriol, and mangiferin. AR treatment significantly reduced blood glucose and lipid levels, ameliorated insulin resistance, attenuated inflammation, and modulated the PPAR and NF-κB signalling pathways. Serum metabolomics analysis revealed 35 differential metabolites, with linoleic acid metabolism and PPAR signalling identified as the predominant metabolic pathways. Molecular docking and MD simulations demonstrated strong binding affinity between core components and key targets (PPARA, NFKB1, IL6, AKT1, IL1B). Pharmacological validation confirmed AR’s therapeutic efficacy in T2DM through regulation of these core targets.

**Conclusion:**

AR ameliorates T2DM by suppressing NF-κB signalling and activating PPAR pathways, thereby improving metabolic dysregulation.

## 1 Introduction

Type 2 diabetes mellitus (T2DM) is a prevalent chronic metabolic disorder associated with an increased risks of cardiovascular and renal complications. Current pharmacological treatments – such as biguanides, sulfonylureas, and GLP-1 receptor agonists - are effective but may pose adverse effects, including hypoglycaemia, bladder cancer, and pancreatitis (1). In contrast, traditional Chinese medicine (TCM) offers a promising alternative for T2DM managements due to its multi-target mechanisms and favourable safety profile, particularly in improving insulin sensitivity and mitigating disease-related complications (2).

Anemarrhenae Rhizoma (AR), derived from the dried rhizome of Anemarrhena asphodeloides Bunge (Liliaceae family), is a widely used TCM with a long-standing history of hypoglycaemic applications. Its use in diabetes management was first documented in Shennong’s Herbal Classic. Modern pharmacological studies demonstrate that AR possesses multiple therapeutic effects, including anti-inflammatory, antioxidant, hypoglycaemic, lipid-lowering, anti-aging, and neuroprotective properties (3). Mangiferin, timosaponin AIII, timosaponin BII, and timosaponin BIII represent the most extensively studied AR components. Our previous research identified that mangiferin, an active component of AR, ameliorates insulin resistance (IR) and hyperglycaemia in T2DM rats by modulating glycerophospholipids (GP), sphingolipids, and arachidonic acid (AA) metabolism in erythrocyte membranes (4). Timosaponins AIII, BII, and BIII exert multi-target anti-diabetic and anti-inflammatory activities in preclinical models via distinct molecular mechanisms. Owing to their inherent physicochemical properties—high molecular weight and limited membrane permeability—these compounds display low oral bioavailability. Consequently, their *in vivo* pharmacological effects are predominantly mediated by metabolites rather than by the parent molecules. Despite AR’s therapeutic potential, its bioactive constituents *in vivo* and their mechanisms of action remain incompletely characterised. It is well established that only blood-absorbed components are likely to form the substantive basis for TCM’s therapeutic effects (5). Notably, metabolites detectable in urine may also represent critical components to TCM’s pharmacological activity. Serum-urine pharmacochemistry, an integrative approach for identifying both blood-absorbed components and their metabolites, is therefore essential for elucidating TCM efficacy. However, AR’s specific bioactive constituents in circulation, their metabolic fate, and their mechanistic roles in T2DM pathophysiology remain insufficiently investigated. Building upon AR’s established effects on metabolic regulation and our preliminary findings regarding mangiferin’s modulation of lipid metabolism, we hypothesise that specific serum-absorbed AR components and their metabolites regulate key metabolic pathways (particularly lipid metabolism) and inflammatory signalling (notably the NF-κB/PPAR axis) in T2DM.

Ultra-high performance liquid chromatography coupled with quadrupole time-of-flight mass spectrometry (UHPLC-Q-TOF-MS) is a leading analytical technique in TCM component analysis and metabolomics, owing to its high sensitivity, resolution, and mass accuracy (6). Metabolomics facilitates the qualitative and quantitative analysis of small-molecule metabolites within biological systems, mapping endogenous compound responses to internal and external environmental stimuli. This approach helps elucidate pathological metabolic and signalling pathways, which are critical for disease mechanisms and drug actions. Network pharmacology employs computational data mining to model multi-layered interactions among TCM bioactive components, therapeutic targets, biological pathways, and diseases. This systems-level perspective reveals synergistic multi-component, multi-target mechanisms, supporting the discovery of efficient, low-toxicity multi-target drugs and mechanistic clarification (7). Complementary techniques, such as molecular docking and molecular dynamics (MD) simulations, predict binding conformations and validate component-target interactions. The selection of optimal binding modes, based on binding energy calculations, further confirms strong affinity interactions.

This study employs a multi-faceted strategy integrating serum-urine pharmacochemistry, network pharmacology, metabolomics, and pharmacological experiments to characterise AR’s *in vivo* active components and elucidate its anti-T2DM mechanisms.

## 2 Materials and methods

### 2.1 Material and reagents

The reference standards of neomangiferin, timosaponin BII, timosaponin D, anemarrhenasaponin I, timosaponin AIV, and timosaponin AIII were obtained from the National Institute for the Control of Pharmaceutical and Biological Products (Beijing, China). Rosiglitazone was procured from Taiji Group Chongqing Peiling Pharmaceutical Co., Ltd. (Chongqing, China). Assay kits for total cholesterol (TC), triacylglycerols (TG), high-density lipoprotein cholesterol (HDL-C), low-density lipoprotein cholesterol (LDL-C), insulin (INS) ELISA, and Haematoxylin-Eosin (HE) staining (batch no. A111-1-1, A110-1-1, A112-1-1, A113-1-1, H203-1-2, and 20160126, respectively) were purchased from Nanjing Jiancheng Bioengineering Institute (Nanjing, China). ELISA kits for tumour necrosis factor-α (TNF-α) (batch no. 0180R1), interleukin-6 (IL-6) (batch no. 0163M1), and interleukin-1β (IL-1β) (batch no. 0047R1) were acquired from Jiangsu Meimian Industrial Co., Ltd. (Jiangsu, China). HRP-conjugated goat anti-rabbit IgG (batch no. SV0002), the diaminobenzidine (DAB) chromogenic kit (batch no. AR1002), and rabbit anti-PPARγ antibody (batch no. 191262) were supplied by Boster Biological Technology, Co., Ltd. (Wuhan, China). Acetonitrile (UHPLC grade) was obtained from Merck (Shanghai, China), while acetic acid and ammonium acetate were sourced from DIMA (Richmond Hill, USA), Leucine-enkephalin, methanol, and streptozotocin (STZ) were purchased from Sigma-Aldrich (Steinheim, Germany). Double-distilled water was procured from Watson’s Food & Beverage (Guangzhou, China). Anemarrhena Rhizoma samples were provided by Zisun Pharmaceutical Co., Ltd. (Guangdong, China) (batch no. 130501). The crude drug was authenticated by Professor Jizhu Liu (Guangdong Pharmaceutical University).

### 2.2 Preparation of AR extract sample

The AR extract was prepared as follows: the dried herb (whole plant or rhizome *of Anemarrhena asphodeloides* Bge) was cut into small pieces and extracted twice (1h per extraction) with 80% ethanol using reflux extraction. The resulting solution was filtered through gauze, and the filtrates were combined and evaporated under reduced pressure to yield an ethanol (EtOH) extract. For further processing, 2.00 g of the AR extract was dispersed in 50 mL of methanol and sonicated for 1 h to ensure complete dissolution. A 1 mL aliquot of this solution was centrifuged at 15,000 rpm at 4 °C for 5 min, and the supernatant was collected for subsequent analysis.

### 2.3 Animals and treatments

Fourty-two male Sprague-Dawley rats (mean body weight: 180 ± 20 g) were obtained from the Medical Experimental Animal Center of Guangdong Province (Foshan, China, No. SCXK 2013-0002). The animals were housed under controlled environmental conditions (temperature: 22 ± 2°C; relative humidity: 55 ± 5%; 12 h light/dark cycle) with ad libitum access to standard laboratory diet and water. Following a minimum 1-week acclimatisation period, we stratified the rats by body weight and randomly assigned them to either: normal control (NC, n=7) or diabetic model (DM, n=35). We induced diabetes in the DM group by administering a high-fat diet (composed of 65% basal rat chow, 10% lard, 20% sucrose, 2.5% cholesterol, 1% mineral mixture, 1% sodium cholate, and 0.5% cellulose mixture) for 6 weeks, followed by a single intraperitoneal injection of 2% streptozotocin (STZ) solution at a dose of 35 mg/kg. Seven days post-injection, we measured fasting blood glucose (FBG) from tail vein blood samples. Rats exhibiting persistent hyperglycaemia (FBG > 11.1 mmol/L) were deemed to have successful diabetes induction (**Supplementary Figure S1**).

The DM model rats were randomly divided into five groups: the DM model group (DM, n=7), AR extract high-, medium- and low-dose groups (ARH, ARM, ARL, n=7 per group), and the rosiglitazone positive control group (ROG, n=7). The DM group and NC group received 0.8 mL/100g body weight of distilled water by oral gavage daily. The ROG group was administered 5 mg**/**kg body weight of rosiglitazone suspension daily, while the ARH, ARM, and ARL groups received 400, 200, and 100 mg/kg body weight of AR extract, respectively, once daily for four weeks. All experimental procedures were conducted in strict accordance with the Guidelines for the Care and Use of Laboratory Animals and were approved by the Institutional Animal Care and Use Committee of Guangdong Pharmaceutical University (Guangzhou, China; Approval no. gdpulacspf2018132).

### 2.4 Biochemical analysis

Following the final administration, FBG, FINS, insulin resistance index (HOMA-IR), insulin sensitivity index (ISI), and lipid levels were measured in each experimental group. Serum concentrations of TNF-α, IL-6, and IL-1β were determined using ELISA.

### 2.5 Pathological changes of liver tissues

Following collection, rat liver tissues were immediately fixed in 10% neutral buffered formalin at a 10:1 (fixative:tissue) volume ratio. Selected tissues samples were then paraffin-embedded and sectioned at 5 μm thickness. Tissue sections were stained with haematoxylin and eosin (H&E), examined by light microscope, and imaged at 400× magnification (40× objective with 10× eyepiece) for analysis.

### 2.6 Rt-qPCR analysis of NF-κB p65 and TGF-β1 mRNA expression in liver tissue

Total mRNA was extracted from liver tissue using TRIzol reagent (Promega). For each sample, 1 μg of total RNA was reverse-transcribed into complementary DNA using a reverse transcription system. RT-qPCR was subsequently performed using the SYBR Green qPCR SuperMix (Invitrogen). The qPCR primer sequences were synthesised by Sangon Biotech (Shanghai) Co., Ltd. and are listed in **Supplementary Table S1**. Following 40 cycles, the relative gene expression levels of nuclear factor-κB (NF-κB) p65 and transforming growth factor-β1 (TGF-β1) were quantified using the 2^-ΔΔCt^ method.

### 2.7 Immunohistochemical analysis of PPARγ expression in perirenal adipose tissue

Prepare paraffin sections from perirenal adipose tissue. Dewax the sections with xylene and rehydrate them through a graded ethanol series. Perform antigen retrieval before incubating the sections in 0.3% H_2_O_2_ solution for 10 minutes to block endogenous peroxidase activity. Wash the sections with distilled water, then block them with 5% BSA solution for 10 minutes. Incubate the sections with primary antibody PPARγ (diluted 1,200) at 37 °C in a humidified chamber for 2 hours. Afterwards, incubate them with HRP-conjugated goat anti-rabbit IgG secondary antibody at 37 °C for 30 minutes. Develop the staining using diaminobenzidine (DAB), counterstain with haematoxylin, then dehydrate, clear, and mount the sections with neutral resin. Examine and photograph the stained sections under an optical microscope using a 10× eyepiece and 40× objective. Analyse the integrated optical density (IOD) using Image-Pro Plus 6.0 software.

### 2.8 Preparation of rat serum and urine sample

On day 7 post-administration, blood samples were collected from the ARH groups via the ophthalmic vein at 30, 60, and 120 minutes after dosing for *in vivo* chemical analysis. Serum was obtained by centrifugation (4000 rpm, 4 °C for 10 minutes), with samples from each time point being pooled and stored at -80°C prior to analysis. Following drug administration, rats were housed in metabolic cages to enable 24-hour urine collection into bottles containing NaN3 (0.05% wt/vol). Urine samples were centrifuged (4000 rpm, 10 minutes, 4°C), and the supernatants were stored at -80°C until UHPLC-MS analysis. After four weeks of AR extract administering, serum samples from each group were collected for metabolomics analysis.

Following thawing, both serum and urine samples were vortex-mixing for 2 minutes and centrifuged (4000 rpm, 10 minutes, 4°C). For serum processing, 1 mL of supernatant was mixed with 3 mL acetonitrile, vortex-mixed, and centrifuged (12,000 rpm, 10 minutes, 4 °C). The resulting supernatant was dried under nitrogen gas, and the residue was redissolved in 200 μL of 50% methanol for analysis. For urine processing, 100 μL of supernatant was mixed with 300 μL of acetonitrile, vortex-mixed, and centrifuged (10,000 rpm, 15 minutes, 4 °C), with 200 μL of the final supernatant collected for analysis.

### 2.9 Instrumentation and conditions

The Waters Acquity™ Ultra Performance LC system (Waters Corporation, Milford, USA) was equipped with a quaternary pump, vacuum degasser, cooled autosampler, and diode-array detector. Chromatographic separation was performed using an Acquity BEH C18 column (50 mm×2.1 mm, 1.7 µm) maintained at 30 °C. The gradient elution programs for AR constituent identification *in vivo* and metabolomics analysis are detailed in **Supplementary Tables S2, S3**, respectively. The flow rate was set to 0.40 ml/min, and the autosampler temperature was maintained at 4 °C. A Waters Micromass Q-TOF Micro™ mass spectrometer (Waters Co., UK), fitted with a LockSpray and ESI interface, was operated in both positive and negative ion modes. The system was controlled using Masslynx data analysis software. The capillary voltage and cone voltage were set to 3000 V and 30 V, respectively, in both ionisation modes. The ion source temperature and desolvation temperature were maintained at 120 °C and 350 °C. Nitrogen (60 L/h) served as the cone gas, while argon (600 L/h) was used as the collision gas. Mass spectrometric data were acquired over a range of 100~1500 Da in both ionisation modes. To ensure mass accuracy and reproducibility, Leucine-enkephalin (Sigma, batch no. L9133-50MG, 600 ng/mL) was used as the lock mass via the LockSpray interface, generating reference ions at m/z 556.2771 [M+H]^+^ (positive mode) and m/z 554.2615 [M-H]^-^ (negative mode). The LockSpray frequency was set to 10 s. For MS/MS experiments, a variable collision energy (20-50 eV) was applied and optimised for each constituent. An Acquity UHPLC-Q-TOF Micro™ system (Waters Co., USA) coupled with MassLynx 4.1 software was used to obtain accurate mass measurements and compositional data for precursor and fragment ions.

### 2.10 Metabolomics analysis

We normalised the sum of the data matrices and imported them into SIMCA-P 14.1 software for pattern recognition analyses, including principal component analysis (PCA) and orthogonal partial least squares-discriminant analysis (OPLS-DA). Using variable importance in projection (VIP) values > 1, we filtered differential variables. We then performed volcano plot analysis, selecting variables with a fold change (FC) > 1.2 or < 0.8 and a Student’s t-test p-value < 0.05. We considered variables meeting all criteria (VIP > 1, FC > 1.2 or < 0.8, and p-value < 0.05) as potential biomarkers. We identified the structures of these potential biomarkers using MS and MS/MS mass spectrometry data, cross-referencing HMDB (http://www.hmdb.ca/), LipidMaps (http://www.lipidmaps.org/), and METLIN databases. Finally, we imported the confirmed biomarkers into MetaboAnalyst 6.0 to generate heatmaps, perform pathway and Pearson correlation analyses, and conduct joint pathway analysis.

### 2.11 Network pharmacology analysis

#### 2.11.1 Prediction of AR active constituents targets and collection of T2DM-related therapeutic targets

The structural formulas and Canonical SMILES of the identified components were obtained using ChemDraw software and online databases such as PubChem (https://pubchem.ncbi.nlm.nih.gov/) and ChemSpider (http://www.chemspider.com/). Potential targets associated with the identified constituents were retrieved through global prediction using databases and online servers, including SwissTargetPrediton (http://www.swisstargetprediction.ch/), STITCH (Version 5.0, http://stitch.embl.de/), CTD (http://ctdbase.org/), ETCM (http://www.tcmip.cn/ETCM/index.php/Home/Index/), BATMAN-TCM (Version 2.0, http://bionet.ncpsb.org/batman-tcm), SEA (http://sea.bkslab.org/), and TargetNet (http://targetnet.scbdd.com/). Only evidence-based targets were included (8). Targets linked to T2DM were sourced from multiple databases, including OMIM (https://omim.org/), GeneCards (https://www.genecards.org/), TTD (http://db.idrblab.net/ttd/), and DisGeNET (http://www.disgenet.org/), using the keywords “Type 2 diabetes metillus” or “Non-Insulin-Dependent diabetes metillus”. Duplicates were removed to compile the final set of potential therapeutic targets for T2DM.

#### 2.11.2 PPI network and enrichment analysis

Both the targets of AR constituents and T2DM-related targets were imported into the UniProt protein database to standardise their official gene symbols. The protein-protein interaction (PPI) network for the common targets between AR’s bioactive constituents and T2DM-related targets was constructed using STRING 12.0 (https://version-12.string-db.org/). The study species was set to Homo sapiens, and the minimum interaction threshold was defined as a confidence score > 0.9 (“highest confidence”). The PPI hub network was generated based on node degree values, which were calculated using the cytoHubba plugin in Cytoscape 3.7.2 software.

Gene ontology (GO) enrichment analysis and KEGG pathway enrichment analysis were performed using DAVID database (https://david.ncifcrf.gov/home.jsp) to elucidate the biological functions of the intersecting targets. GO biological processes and KEGG pathways with a *P*-value < 0.01 were identified and subsequently analysed using the bioinformatics cloud platform (http://www.bioinformatics.com.cn/) for visualisation. T2DM-related pathways were further extracted based on their association with the bioactive constituents of AR and their corresponding effective targets. A “components-targets-pathways” network was constructed using Cytoscape 3.7.2. The degree value and betweenness centrality parameters were applied to evaluate the significance of each node within the network.

### 2.12 Integrated analysis of metabolomics and network pharmacology

Differential metabolic biomarkers were imported into the MetaboAnalyst 6.0 database and the Metscape plugin (Cytoscape 3.7.2) to identify metabolically related targets. Venn diagrams were used to identify common targets between the potential targets from network pharmacology and those associated with metabolism. The metabolic biomarkers and common targets were further analysed in MetaboAnalyst 6.0 for joint pathway analysis. Subsequently, the metabolic biomarkers and key-associated metabolic pathways were integrated to construct a “metabolites-key targets-pathways” network. This network was developed to elucidate the mechanistic role of AR in the treatment of T2DM.

### 2.13 Molecular docking

To further assess whether AR bioactive constituents and T2DM-related targets exhibit strong binding activity, we performed molecular docking using AutoDock 4.2.6. We screened the top target proteins based on the contribution degree values of the nodes in the “metabolites-key targets-pathways” network. We obtained the target protein files in PDB format from the RCSB PDB database (http://www1.rcsb.org/) and pre-processed the original protein structures using PyMOL. For the AR bioactive components, we either retrieved their 3D structures from the PubChem database or drew them using Chemoffice 19.0, then converted them to PDB format and minimised their energy with Chem3D 19.0. We evaluated the binding affinity between AR bioactive components and T2DM-related target proteins based on binding energy. According to existing literature, a binding energy < -4.25 kcal/mol suggests good ligand-receptor, and the while a value < -7.0 kcal/mol indicates strong binding activity (9, 10).

### 2.14 Molecular dynamics simulations

GROMACS 2022 was employed to run 100 ns molecular dynamics (MD) simulations of complexes between AR bioactive constituents and representative core targets, assessing their binding stability and dynamic behavior. The protein parameters were derived from the CHARMM36 force field, while we generated the ligand topology using the GAFF2 force field. We applied periodic boundary conditions and solvated the protein-ligand complex in a cubic box with TIP3P water molecules, maintaining a 1.2 nm periodic boundary. For electrostatic interactions, we used the Particle Mesh Ewald (PME) method and the Verlet algorithm. The system was equilibrated in two phases (100 ps each, 0.1 ps coupling constant): NVT (isothermal-isochoric) and NPT (isothermal-isobaric) ensembles. We calculated both van der Waals and Coulomb interactions using a 1.0 nm cutoff. Finally, we ran the production MD simulation using GROMACS 2022 under constant conditions (310 K, 1 bar) for 100 ns using GROMACS 2022.

### 2.15 Statistical analysis

Data from each group were analyzed statistically using GraphPad Prism 8.0 software. Measurement data are presented as mean ± standard deviation (SD), and comparisons among multiple groups were performed using one-way analysis of variance (ANOVA) followed by Tukey’s *post-hoc* test for pairwise comparisons. A P-value of less than 0.05 was considered statistically significant. To enhance the interpretability of results, effect sizes (Cohen’s d) were calculated to quantify group differences, and 95% confidence intervals (CIs) for means were reported together with point estimates. Power analysis was performed using GPower 3.1.9.7 to determine the sample size prior to the experiment. For a one-way ANOVA with an expected medium effect size (f = 0.25), α = 0.05, and 80% power, a minimum of 5 rats per group was required. The final sample size (n = 7 per group) exceeded this estimate to account for potential biological variability.

## 3 Results

### 3.1 AR treatment ameliorates STZ-induced T2DM in rats

After 28 days of treatment, the blood glucose and lipid levels of each experimental group are detailed in **Table 1**. Compared with the NC group, the DM group exhibited significantly elevated PBG, FINS, HOMA-IR, TC, TG, and LDL-C levels (P<0.01), indicating severe IR in the DM model rats. This was accompanied by impaired insulin utilisation, hyperinsulinaemia, and dyslipidaemia. FBG levels were significantly reduced in the ARH, ARM, and ROG groups compared with the DM group. Furthermore, FINS, HOMA-IR, TC, TG, and LDL-C levels were significantly decreased in all AR dosage groups and ROG group, while ISI and HDL-C levels were markedly increased. These results indicated that AR significantly improves IR and partially reverses lipid metabolic disorders in DM model rats, thereby ameliorating diabetic lipotoxicity.

**Table 1:** AR effects on blood glucose and lipid levels in rat serum ( $\bar{x}$ ± *s, n=*7 ).

Group	Dose (mg/kg)	FBG (mmol/L)	FINS (mU/L)	TC (mmol/L)	TG (mmol/L)	LDL-C (mmol/L)	HDL-C (mmol/L)	HOMA-IR	ISI
		Cohen’s d/95%CI	Cohen’s d/95%CI	Cohen’s d/95%CI	Cohen’s d/95%CI	Cohen’s d/95%CI	Cohen’s d/95%CI		
NC	0	4.68 ± 0.41	14.29 ± 1.15	2.19 ± 0.31	0.68 ± 0.09	0.79 ± 0.13	1.76 ± 0.12	1.08 ± 0.16	-4.20 ± 0.16
DM	0	24.13 ± 1.04^##^ 24.61[18.44, 20.47]	29.15 ± 3.07^##^ 6.41[11.88, 17.84]	4.67 ± 0.43^##^ 6.62[2.00, 2.96]	1.61 ± 0.19^##^ 6.26[0.74, 1.12]	1.93 ± 0.13^##^ 8.77[0.97, 1.37]	0.95 ± 0.10^##^ -7.33[-0.95, -0.67]	3.44 ± 0.08^##^	-6.55 ± 0.08^##^
ROG	5	18.82 ± 1.40** 4.31[3.65, 6.98]	18.65 ± 0.90** 4.64[7.26, 13.73]	3.50 ± 0.16** 3.61[0.71, 1.64]	1.19 ± 0.06** 2.98[0.22, 0.61]	1.31 ± 0.07** 5.94[0.47, 0.77]	1.49 ± 0.04** -7.09[-0.65, -0.44]	2.62 ± 0.13**	-5.73 ± 0.13**
ARH	400	17.30 ± 2.41** 3.68[4.49, 9.17]	15.56 ± 1.07** 5.91[10.88, 16.30]	2.53 ± 0.13** 6.74[1.76, 2.51]	0.81 ± 0.06** 5.68[0.63, 0.95]	0.96 ± 0.11** 8.06[0.82, 1.12]	1.58 ± 0.09** -6.62[-0.75,-0.51]	2.65 ± 0.17**	-5.76 ± 0.17**
ARM	200	20.64 ± 3.02* 1.55[0.63, 6.35]	17.71 ± 1.10** 4.96[8.72, 14.16]	3.44 ± 0.19** 3.70[0.84, 1.63]	1.14 ± 0.07** 3.28[0.30, 0.63]	1.10 ± 0.10** 7.16[0.69, 0.97]	1.40 ± 0.09** -4.73[-0.56, -0.33]	2.85 ± 0.12**	-5.96 ± 0.12**
ARL	100	22.98 ± 1.45 0.91[-0.47, 2.77]	20.89 ± 1.64** 3.36[5.10, 11.42]	3.65 ± 0.19** 3.07[0.59, 1.45]	1.28 ± 0.08** 2.26[0.15, 0.52]	1.42 ± 0.08** 4.73[0.37, 0.66]	1.33 ± 0.06** -4.61[-0.48,-0.27]	2.88 ± 0.09**	-5.99 ± 0.09**

Data are expressed as mean ± SD. Compared with the NC group, ^##^P<0.01; Compared with the DM group, *P < 0.05, **P < 0.01.

NC, normal control group; DM, diabetes mellitus group; ROG, rosiglitazone group; ARH, high-dose AR group; ARM, medium-dose AR group; ARL, low-dose AR group; FBG, fasting blood glucose; FINS, fasting insulin; HOMA-IR, insulin resistance index; ISI, insulin sensitivity index; TC, total cholesterol; TG, triglycerides; LDL-C, low-density lipoprotein cholesterol; HDL-C, high-density lipoprotein cholesterol. Cohen’s d is a standardized effect size measuring the magnitude of the mean difference between groups. According to Cohen’s standards, |*d|*≥ 0.8 indicates a large effect size, and all intergroup comparisons in this study show significant differences. 95%CI, 95% confidence interval of mean difference (mmol/L).

**Table 2** reveals that the DM model group developed significantly elevated serum inflammatory factor levels and increased NF-κB and TGF-β1 expression, establishing a pronounced inflammatory state in diabetic rats. Compared to the DM group, the ARH, ARM and ROG groups exhibited significantly reduced TNF-α, IL-6 and IL-1β levels. All AR dosage groups and ROG group demonstrated substantially decreased hepatic NF-κB and TGF-β1 mRNA expression, with the ARH group showing the most marked reduction. These results confirm that AR inhibits the NF-κB signalling pathway, suppresses inflammatory factor generation, and ameliorate the inflammatory status in T2DM. *Post hoc* comparisons of pharmacodynamic parameters among experimental groups are shown in **Supplementary Table S4**.

**Table 2:** AR effects on serum inflammatory cytokines (TNF-α, IL-6, IL-1β) and hepatic NF-κB p65 and TGF-β1 mRNA expression in rats ( $\bar{x}$ ± *s, n=*7 ).

Group	Dose (mg/kg)	TNF-α (pg/mL)	IL-6 (pg/mL)	IL-1β (pg/mL)	NF-κB mRNA	TGF-β1 mRNA
		Cohen’s d/95%CI	Cohen’s d/95%CI	Cohen’s d/95%CI		
NC	0	73.15 ± 17.75	21.98 ± 10.33	125.47 ± 18.28	0.48 ± 0.04	0.38 ± 0.06
DM	0	177.95 ± 35.31^##^ 3.75[82.15, 127.46]	114.32 ± 27.09^##^ 4.50[59.28, 125.39]	251.99 ± 25.89^##^ 5.65[97.69, 155.34]	18.11 ± 3.05^##^	35.36 ± 6.49^##^
ROG	5	128.28 ± 15.32** 1.83[23.46, 75.89]	81.02 ± 15.33* 1.51[39.98, 78.11]	145.06 ± 28.15** 3.95[72.13, 141.71]	2.32 ± 0.31**	2.76 ± 0.61**
ARH	400	116.26 ± 41.01** 1.61[26.56, 96.82]	73.19 ± 16.04* 1.85[31.53, 70.89]	134.64 ± 34.80** 3.83[77.90, 156.80]	0.63 ± 0.07**	0.53 ± 0.05**
ARM	200	138.71 ± 36.99* 1.09[4.24, 74.25]	79.37 ± 16.44* 1.56[37.37, 77.42]	173.12 ± 29.77** 2.83[42.98, 114.75]	1.53 ± 0.31**	3.81 ± 0.86**
ARL	100	163.27 ± 10.74 0.56[-11.18, 40.55]	95.99 ± 25.84 0.69[45.31, 102.72]	219.40 ± 23.47 1.32[0.81, 64.38]	1.98 ± 0.62**	3.77 ± 1.52**

Data are expressed as mean ± SD. Compared with the NC group, ^##^P<0.01; Compared with the DM group, *P < 0.05, **P < 0.01.

NC, normal control group; DM, diabetes mellitus group; ROG, rosiglitazone group; ARH, high-dose AR group; ARM, medium-dose AR group; ARL, low-dose AR group; TNF-α, tumor necrosis factor-α; IL-6, interleukin 6; IL-1β, interleukin-1β; NF-κB, Nuclear factor-kappa B; TGF-β1, transforming growth factor-β1. Cohen’s d is a standardized effect size measuring the magnitude of the mean difference between groups. According to Cohen’s standards, |*d|*≥ 0.8 indicates a large effect size. 95%CI, 95% confidence interval of mean difference (pg/mL).

**Figure 1:**
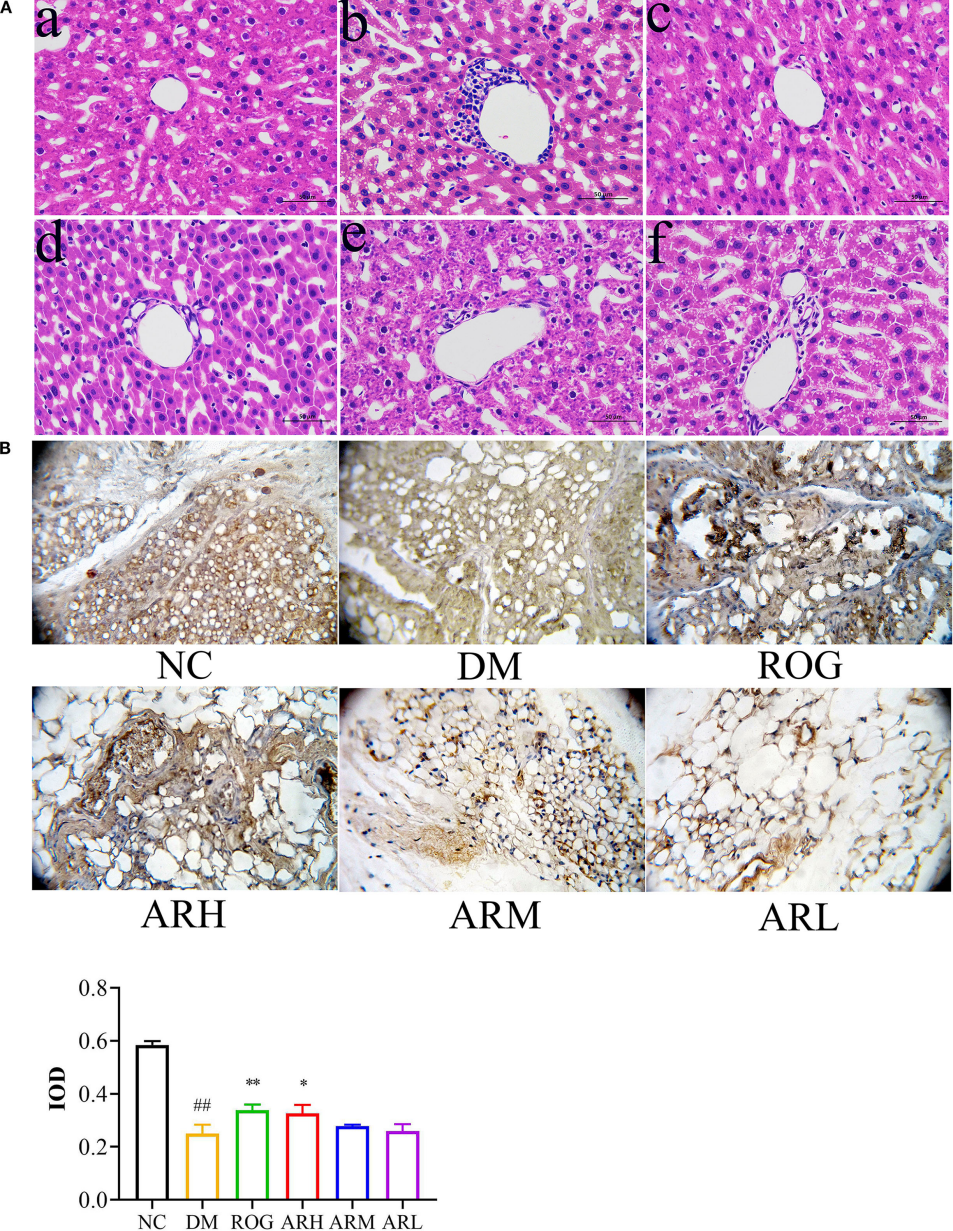
AR exhibited therapeutic effects in STZ-induced T2DM rats. **(A)** Pathological changes of liver tissues (at 400× magnification). **(B)** Immunohistochemical analysis of PPARγ expression in perirenal adipose tissue. The positively stained cells exhibited a brown coloration. Positive expression regions of PPARγ in perirenal adipose tissues were measured by IOD value. Data are expressed as mean ± SD (n = 7). Compared with the NC group, ##P<0.01; Compared with the DM group, *P < 0.05, **P < 0.01. (a) NC group, (b) DM group, (c) ROG group, (d) ARH group, (e) ARM group, (f) ARL group. NC, normal control group; DM, diabetes mellitus group; ROG, rosiglitazone group; ARH, high-dose AR group; ARM, medium-dose AR group; ARL, low-dose AR group.

Liver tissue pathological sections from rats in each group are presented in **Figure 1A**. In the NC group, the liver tissue structure is intact and clearly defined, with hepatic cords arranged radially around the central vein. The liver sinusoids are distinct, hepatocytes are uniform in size and well-organised, cell boundaries are sharp, the cytoplasm exhibits even eosinophilic (red) staining, and nuclei are centrally located without signs of inflammatory cell infiltration. In the DM model group, granular lesions are visible within hepatocytes, along with numerous round fat droplets of varying sizes in the cytoplasm, accompanied by inflammatory cell infiltration. Compared to the DM group, the ARH and ROG groups show no significant inflammatory infiltration or granular lesions, and diffuse microvesicular steatosis is absent. In the ARM and ARL groups, mild inflammatory cell infiltration is observed, along with a reduction in granular lesions. However, the ARL group still displays substantia diffuse fat droplet formation.

The positive expression rate of PPARγ protein in the adipose tissue of the DM group was significantly lower than that of the NC group (P<0.01) (**Figure 1B**). Immunohistochemical analysis of perirenal adipose tissues revealed a marked increase in PPARγ protein expression in the ROG and ARH group (P<0.05, P<0.01). These findings suggest that AR ameliorates IR and enhances adipose tissue insulin sensitivity by upregulating PPARγ expression in adipocytes.

### 3.2 Metabolomics analysis

**Figure 2:**
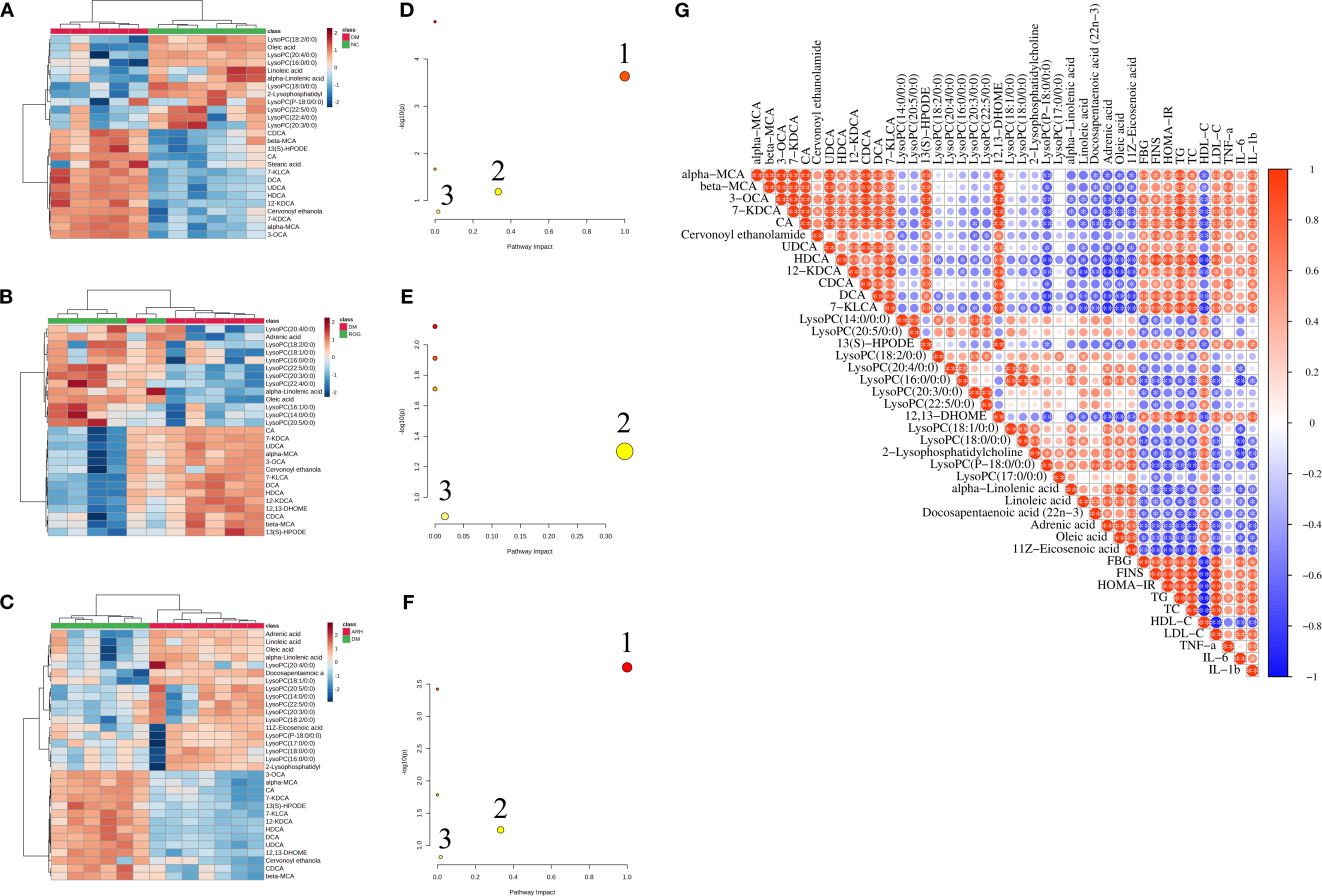
AR regulated the metabolic characteristics of serum in STZ-induced T2DM rats. **(A–C)** Heatmaps of differential metabolites in serum samples between NC, DM, ROG, and ARH groups. **(D–F)** Main metabolic pathways of differential metabolites in serum samples among the groups. 1. Linoleic acid metabolism, 2. alpha-Linolenic acid metabolism, 3. Glycerophospholipid metabolism. **(G)** Pearson correlation analysis of differential metabolites and biochemical indicators between the DM and the ARH groups. *P<0.05, **P<0.01. NC, normal control group; DM, diabetes mellitus group; ROG, rosiglitazone group; ARH, high-dose AR group; ARM, medium-dose AR group; ARL, low-dose AR group.

The screening process identified 35 differential serum metabolites in both positive and negative ion modes, including eleven bile acids, fourteen lysophosphatidylcholines (LysoPCs), nine fatty acids (FAs), and one cervonoyl ethanolamide (see **Table 3** for details). The methodological validation and identification process of metabolomics are detailed in the **Supplementary Materials**. Using the relative peak area of each metabolite, we generated a heatmap to visualise level changes across groups (**Figures 2A–C**). Compared to the NC group, the DM group exhibited higher serum levels of bile acids, cervonoyl ethanolamide, 13(S)-HPODE, and stearic acid but lower levels of LysoPCs and oleic acid. Following AR and ROG treatment, serum levels of bile acids, 13(S)-HPODE, and 12,13-DHOME decreased, whereas LysoPCs and unsaturated fatty acids (UFAs) increased. We further analysed the differential metabolites in MetaboAnalyst 6.0 for pathway enrichment. Three pathways showed significant alterations (impact value > 0.01; **Figures 2D–F**): linoleic acid metabolism, alpha-linolenic acid metabolism, and glycerophospholipid metabolism.

**Table 3:** The details of identified biomarkers of AR treatment of T2DM in both positive and negative ion modes.

No.	t_R_/min	Ion mode	Mass(*m/z*)		Error (ppm)	Molecular formula	Identification	HMDB ID	Relative change		
			Calculated	Measured					DM *vs.* NC	ROG *vs.* DM	ARH *vs.* DM
1	7.27	[M-H]^-^	407.2797	407.2782	-3.7	C_24_H_40_O_5_	α-Muricholic acid (α-MCA)	HMDB0000506	↑^##^	↓^**^	↓^**^
2	7.95	[M-H]^-^	407.2797	407.2798	0.2	C_24_H_40_O_5_	β-Muricholic acid (β-MCA)	HMDB0000415	↑^##^	↓^**^	↓^**^
3	8.20	[M+H]^+^	407.2797	407.2789	-2.0	C_24_H_38_O_5_	3-Oxocholic acid (3-OCA)	HMDB0000502	↑^##^	↓^**^	↓^**^
	8.14	[M-H]^-^	405.2641	405.2652	2.7						
4	9.32	[M+NH_4_]^+^	424.3063	424.3072	2.1	C_24_H_38_O_5_	7-Ketodeoxycholic acid (7-KDCA)	HMDB0000391	↑^##^	↓^**^	↓^**^
		[M-H]^-^	405.2641	405.2647	1.5						
5	9.53	[M+NH_4_]^+^	426.3219	426.3210	-2.1	C_24_H_40_O_5_	Cholic acid (CA)	HMDB0000619	↑^##^	↓^**^	↓^**^
	9.54	[M-H]^-^	407.2797	407.2792	-1.2						
6	9.55	[M+H]^+^	373.2743	373.2754	2.9	C_24_H_36_O_3_	Cervonoyl ethanolamide	HMDB0013627	↑^##^	↓^*^	↓^*^
7	11.02	[M-H]^-^	391.2848	391.2845	-0.8	C_24_H_40_O_4_	Ursodeoxycholic acid (UDCA)	HMDB0000946	↑^##^	↓^**^	↓^**^
8	11.13	[M-H]^-^	391.2848	391.2862	3.6	C_24_H_40_O_4_	Hyodeoxycholic acid (HDCA)	HMDB0000733	↑^##^	↓^**^	↓^**^
9	11.51	[M+H]^+^	391.2848	391.2867	4.9	C_24_H_38_O_4_	12-Ketodeoxycholic acid (12-KDCA)	HMDB0000328	↑^##^	↓^**^	↓^**^
10	12.47	[M-H]^-^	391.2848	391.2840	-2.0	C_24_H_40_O_4_	Chenodeoxycholic acid (CDCA)	HMDB0000518	↑^##^	↓^**^	↓^**^
11	12.63	[M+H]^+^	468.3090	468.3104	3.0	C_22_H_46_NO_7_P	LysoPC(14:0/0:0)	HMDB0010379	–	↑^*^	↑^*^
12	12.82	[M+NH_4_]^+^	410.3270	410.3260	-2.4	C_24_H_40_O_4_	Deoxycholic acid (DCA)	HMDB0000626	↑^##^	↓^**^	↓^**^
		[M-H]^-^	391.2848	391.2830	-4.6						
13	12.90	[M+NH_4_]^+^	408.3114	408.3102	-2.9	C_24_H_38_O_4_	7-Ketolithocholic acid (7-KLCA)	HMDB0000467	↑^##^	↓^**^	↓^**^
		[M-H]^-^	389.2692	389.2711	4.9						
14	12.98	[M+H]^+^	542.3247	542.3267	3.7	C_28_H_48_NO_7_P	LysoPC(20:5/0:0)	HMDB0010397	–	↑^**^	↑^*^
15	13.25	[M+H]^+^	494.3247	494.3259	2.4	C_24_H_48_NO_7_P	LysoPC(16:1/0:0)	HMDB0010383	–	↑^*^	–
16	13.77	[M-H]^-^	311.2222	311.2221	-0.3	C_18_H_32_O_4_	13(S)-HPODE	HMDB0003871	↑^##^	↓^**^	↓^**^
17	13.92	[M+H]^+^	520.3403	520.3389	-2.7	C_26_H_50_NO_7_P	LysoPC(18:2/0:0)	HMDB0010386	↓^##^	↑^*^	↑^**^
		[M+HCOO]^-^	564.3301	564.3305	0.7						
18	13.98	[M+H]^+^	544.3403	544.3402	-0.2	C_28_H_50_NO_7_P	LysoPC(20:4/0:0)	HMDB0010395	↓^##^	↑^**^	↑^*^
		[M+HCOO]^-^	588.3301	588.3300	-0.2						
19	14.27	[M+H]^+^	496.3403	496.3405	0.4	C_24_H_50_NO_7_P	LysoPC(16:0/0:0)	HMDB0010382	↓^#^	↑^**^	↑^**^
		[M+HCOO]^-^	540.3301	540.3325	4.4						
20	14.34	[M+H]^+^	546.3560	546.3563	0.5	C_28_H_52_NO_7_P	LysoPC(20:3/0:0)	HMDB0010393	↓^#^	↑^**^	↑^*^
21	14.41	[M+H]^+^	570.3560	570.3577	3.0	C_30_H_52_NO_7_P	LysoPC(22:5/0:0)	HMDB0010402	↓^##^	↑^**^	↑^*^
22	14.52	[M-H]^-^	313.2379	313.2380	0.3	C_18_H_34_O_4_	12,13-DHOME	HMDB0004705	–	↓^**^	↓^**^
23	15.15	[M+H]^+^	522.3560	522.3544	-3.1	C_26_H_52_NO_7_P	LysoPC(18:1/0:0)	HMDB0002815	–	↑^**^	↑^*^
		[M+HCOO]^-^	566.3458	566.3453	-0.9						
24	15.30	[M+HCOO]^-^	568.3614	568.3601	-2.3	C_26_H_54_NO_7_P	LysoPC(18:0/0:0)	HMDB0010384	↓^##^	–	↑^**^
25	15.44	[M+H]^+^	572.3716	572.3717	0.2	C_30_H_54_NO_7_P	LysoPC(22:4/0:0)	HMDB0010401	↓^#^	↑^**^	–
26	15.57	[M+HCOO]^-^	568.3614	568.3598	-2.8	C_26_H_54_NO_7_P	2-Lysophosphatidylcholine	HMDB0258493	↓^##^	–	↑^**^
27	15.63	[M+H]^+^	508.3767	508.3753	-2.8	C_26_H_54_NO_6_P	LysoPC(P-18:0/0:0)	HMDB0013122	↓^##^	–	↑^*^
28	15.68	[M+H]^+^	510.3560	510.3554	-1.2	C_25_H_52_NO_7_P	LysoPC(17:0/0:0)	HMDB0012108	–	–	↑^*^
29	16.41	[M-H]^-^	277.2168	277.2159	-3.2	C_18_H_30_O_2_	α-Linolenic acid (ALA)	HMDB0001388	↓^#^	↑^*^	↑^*^
30	17.19	[M-H]^-^	279.2324	279.2338	5.0	C_18_H_32_O_2_	Linoleic acid (LA)	HMDB0000673	↓^#^	–	↑^*^
31	17.46	[M-H]^-^	329.2481	329.2466	-4.6	C_22_H_34_O_2_	Docosapentaenoic acid (22n-3) (DPA)	HMDB0006528	–	–	↑^*^
32	17.83	[M-H]^-^	331.2637	331.2639	0.6	C_22_H_36_O_2_	Adrenic acid (ADA)	HMDB0002226	–	↑^*^	↑^**^
33	18.06	[M-H]^-^	281.2481	281.2490	3.2	C_18_H_34_O_2_	Oleic acid (OA)	HMDB0000207	↓^##^	↑^**^	↑^**^
34	18.95	[M-H]^-^	283.2637	283.2631	-2.1	C_18_H_36_O_2_	Stearic acid (SA)	HMDB0000827	↑^#^	–	–
35	19.01	[M-H]^-^	309.2794	309.2785	-2.9	C_20_H_38_O_2_	11Z-Eicosenoic acid	HMDB0002231	–	–	↑^**^

NC, normal control group; DM, diabetes mellitus group; ROG, rosiglitazone group; ARH, high-dose AR group; Compared with those in the control group, ^##^P < 0.01, ^#^P< 0.05; Compared with the DM model group, ^**^P < 0.01, ^*^P < 0.05. The relative levels of potential biomarkers were denoted as up-regulated (↑) or down-regulated (↓).

We used Pearson correlation analysis to assess the potential relationships between differential metabolites and biochemical indicators in the DM model group and the ARH group. As illustrated in **Figure 2G**, bile acids, 13(S)-HPODE, and 12,13-DHOME showed significant positive correlations with FBG, FINS, HOMA-IR, TC, TG, LDL-C, TNF-α, IL-6, and IL-1β, but significant negative correlations with HDL-C. Conversely, LysoPCs and UFAs exhibited significant negative associations with FBG, FINS, HOMA-IR, TC, TG, LDL-C, IL-6, and IL-1β, while displaying significant positive correlations with HDL-C.

### 3.3 Identification of serum and urine constituents from AR in T2DM rats

We identified 77 constituents by comprehensively analysing their retention behaviour, MS and MS/MS fragmentation patterns, and comparing these with reference standards and published literature. Among these, 47 prototypes were detected in AR extracts and 11 metabolites in rat serum and urine (**Supplementary Tables S5, S6**). Details of the identification process for these constituents are provided in the **Supplementary Materials**.

### 3.4 Network pharmacology

**Figure 3:**
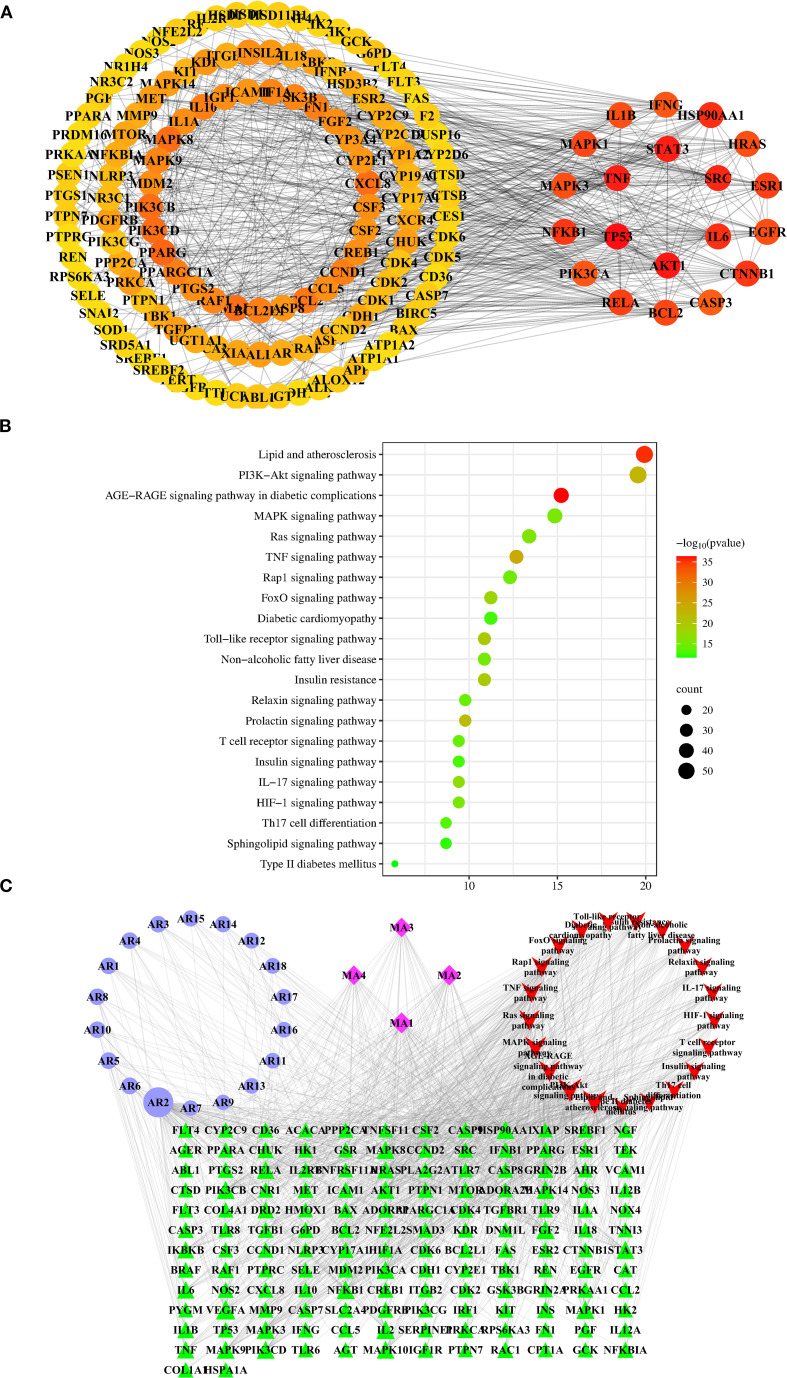
Network pharmacology analysis of AR against T2DM. **(A)** The top 150 targets of the interactive PPI network of AR targets and T2DM targets. Identification of 20 hub targets of AR in treating T2DM based on degree≥20, BC>0.014, and CC>0.357. BC, betweenness centrality; CC, closeness centrality. **(B)** The top 21 pathways of KEGG enrichment analysis of the targets of the bioactive components of AR. **(C)** “Components-targets-pathways” network of AR in treatment of T2DM. The abbreviations of the corresponding components are listed in **Supplementary Table S7**.

We identified 22 *in vivo* migrant compounds (excluding structurally identical ones), consisting of 18 prototype compounds and 4 metabolites (**Supplementary Table S7**), through serum-urine pharmacochemical analysis. These compounds were then analyzed using network pharmacology to investigate their potential mechanisms of action. Our analysis predicted 528 unique component targets after redundancy removal. From disease databases, we identified 2645 T2DM-related targets (selected at twice the median degree threshold). The intersection of these datasets yielded 276 potential therapeutic targets for AR against T2DM. The resulting PPI network contained 231 nodes and 1700 interaction edges, with the top 150 targets shown in **Figure 3A**. In this network, target centrality is visually represented by color intensity, where redder hues indicate greater proximity to the network core. The top 20 highest-degree targets in the PPI network were: TP53, AKT1, STAT3, TNF, SRC, HSP90AA1, IL6, CTNNB1, NFKB1, ESR1, RELA, MAPK1, BCL2, HRAS, MAPK3, IL1B, EGFR, PIK3CA, IFNG, and CASP3. (see **Supplementary Table S8** for their characteristic parameters).

To elucidate the mechanisms by which AR treats T2DM, we performed GO functional analysis and KEGG pathway enrichment analysis. The results demonstrate that the AR’s bioactive constituents are involved in 1,046 biological processes (BPs), 114 cellular components (CCs), and 203 molecular functions (MFs). The top 20 GO items in each category (BP, CC, MF) are summarised in **Supplementary Figure S2**.

From the 195 signalling pathways enriched in the KEGG analysis, we identified the top 21 pathways most relevant to T2DM (**Figure 3B**, **Supplementary Table S9**). These pathways were ranked by their association with T2DM, with the most significant including: AGE-RAGE signalling in diabetic complications, lipid metabolism and atherosclerosis, TNF signalling, PI3K-Akt signalling, prolactin signalling, Toll-like receptor signalling, insulin resistance, FoxO signalling, IL-17 signalling, and Ras signalling. From these pathways, we extracted 146 T2DM- associated target proteins, which were then used to select corresponding potential bioactive compounds for constructing the subsequent network pharmacology framework.

We constructed a “components–targets–pathways” network comprising 189 nodes (146 T2DM-related targets, 22 potential components, and the top 21 T2DM-related pathways) and 1043 edges (**Figure 3C**). Using the “Network Analyzer” plugin in Cytoscape, we analyzed the network’s topological properties. In this visualisation, nodes importance is indicated by: higher degree values, greater betweenness centrality, larger areas size, and darker colours intensity. The analysis revealed the top 5 candidate components with the highest degree values: mangiferin (AR2; degree: 94; betweenness centrality: 0.29042967), norathyriol (MA1; degree: 39; betweenness centrality: 0.05882475), digitogenin (MA2; degree: 36; betweenness centrality: 0.04215628), markogenin or neogitogenin (MA3; degree: 30; betweenness centrality: 0.02774043), and sarsasapogenin (MA4; degree: 30; betweenness centrality: 0.03258071). These components represent AR’s primary bioactive constituents for T2DM treatment.

### 3.5 Integrated analysis of metabolomics and network pharmacology

**Figure 4:**
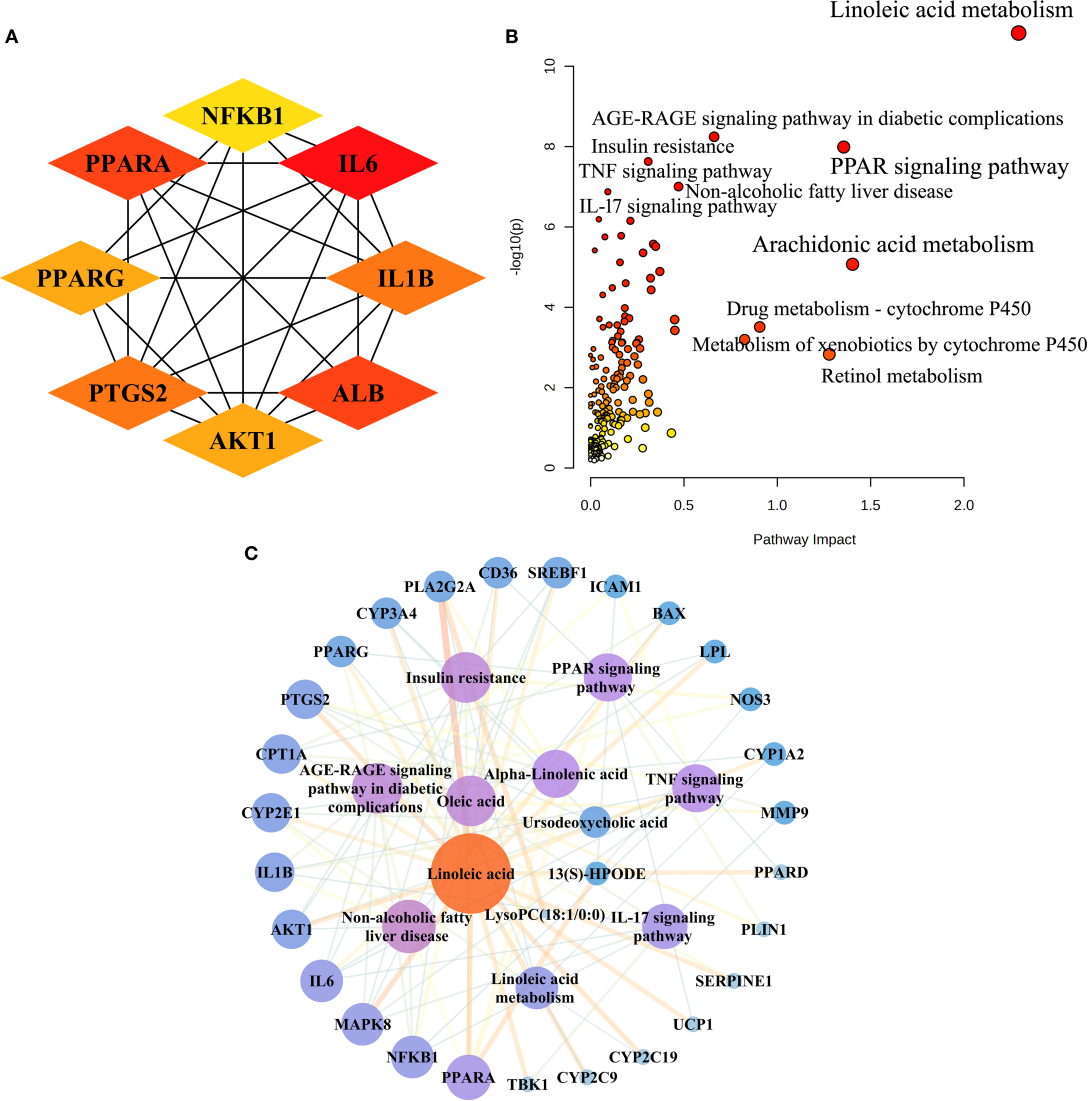
Integrated analysis of metabolomics and network pharmacology. **(A)** The top eight targets identified in the interactive PPI network of metabolic targets and component-disease intersection targets. **(B)** Joint pathway analysis of differential metabolites and shared metabolic targets between the ARH and DM groups. **(C)** The integrative “metabolites-targets-pathways” network underlying the therapeutic effects of AR in treating T2DM.

To elucidate the molecular mechanisms of AR in treating T2DM, we collected 489 metabolic targets. Using Venn diagrams, we performed an intersection analysis between component-disease targets and metabolic targets, identifying 52 common targets. We then analysed these 52 targets further via PPI network analysis (STRING 12.0). With the Cytoscape cytoHubba plugin, we selected the top eight genes based on network centrality: IL6, ALB, PPARA, PTGS2, IL1B, PPARG, AKT1, and NFKB1 (**Figure 4A**).

Next, we performed an integrative pathway analysis using MetaboAnalyst to combine the differential metabolites and common targets. This revealed key signalling pathways involved in AR’s therapeutic mechanisms, including linoleic acid metabolism, the PPAR signalling pathway, the AGE-RAGE signalling pathway in diabetic complications, insulin resistance, non-alcoholic fatty liver disease (NAFLD), the TNF signalling pathway, and the IL-17 signalling pathway (**Figure 4B**). Among the 52 common targets, 27 showed strong associations with the top seven T2DM-related pathways.

We constructed a “metabolites-targets-pathways” network, incorporating six key metabolites, 27 associated targets, and seven metabolic signalling pathways (**Figure 4C**). By analysing the PPI network and conducting joint pathway analysis, we identified five core targets—PPARA, NFKB1, IL6, AKT1, and IL1B—along with three key metabolites (linoleic acid, oleic acid, and α-linolenic acid) as central network components. These results indicate that these core targets and metabolites likely play pivotal roles in AR’s therapeutic mechanism against T2DM.

### 3.6 Molecular docking

**Figure 5:**
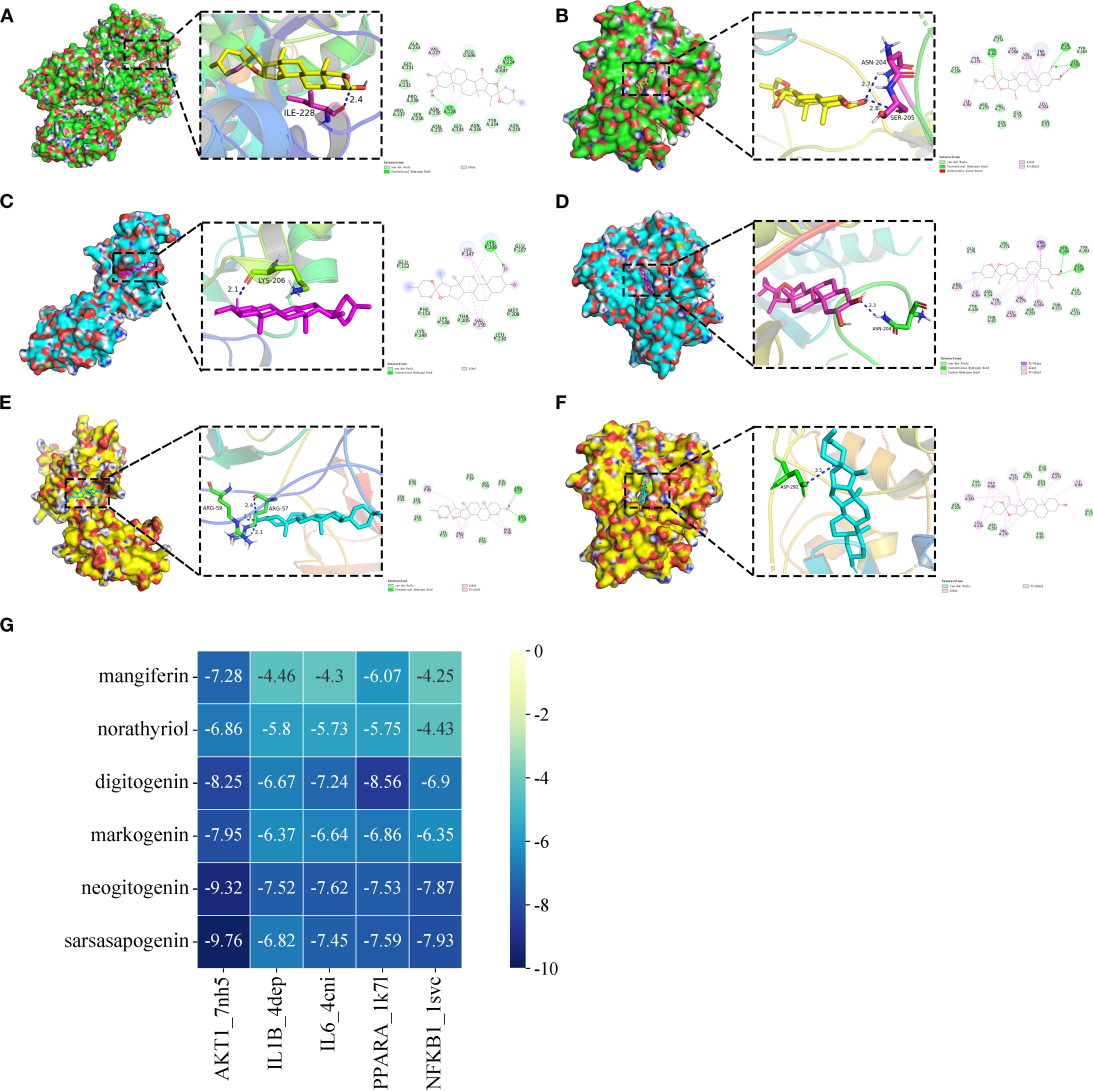
The optimum binding mode (2D and 3D conformation) of partial hub targets dock with potential bioactive components (The selected binding energies ≤ -7.8 kcal/mol). **(A)** digitogenin-PPARA (PDB ID 1K7L). **(B)** digitogenin-AKT1 (PDB ID 7NH5). **(C)** neogitogenin-NFKB1 (PDB ID 1SVC). **(D)** neogitogenin-AKT1 (PDB ID 7NH5). **(E)** sarsasapogenin-NFKB1 (PDB ID 1SVC). **(F)** sarsasapogenin-AKT1 (PDB ID 7NH5). **(G)** Heat maps of molecular docking results.

We performed molecular docking validation using AutoDockTools-1.5.6 to assess interactions between AR’s top five candidate components (one prototype and four metabolites) and five key target proteins from the joint-analysis network. Most component-target interactions showed binding energies below -5.0 kcal/mol, except for mangiferin with IL1B, IL6, and NFKB1, and norathyriol with NFKB1, which exhibited slightly higher energies (>-4.25 kcal/mol). Notably, mangiferin demonstrated particularly strong binding affinities with AKT1 (-7.28 kcal/mol) and PPARA (-6.07 kcal/mol). The steroidal saponin metabolites displayed optimal binding with AKT1, PPARA, and NFKB1 through multiple interactions, including: conventional hydrogen bonds, van der Waals forces, alkyl and pi-alkyl interactions, pi-sigma interactions, carbon-hydrogen bonds. **Figure 5** illustrates the optimal binding modes and docking results. Key interactions included: Sarsasapogenin forming two hydrogen bonds with the NFKB1 (ARG57 and ARG59). Neogitogenin binding to AKT1 (ASN204) and NFKB1 (LYS206). Digitogenin interacting with AKT1 (ASN204, SER205) and PPARA (ILE228).

### 3.7 Molecular dynamics simulations analysis

Based on the molecular docking results, sarsasapogenin-AKT1 and digitogenin-PPARA complexes exhibited the highest binding scores. To further assess the binding stability between sarsasapogenin, digitogenin, and their respective target proteins (AKT1 and PPARA), we analyzed key parameters including root mean square deviation (RMSD), root mean square fluctuation (RMSF), number of hydrogen bonds, radius of gyration (Rg), and solvent-accessible surface area (SASA).

**Figure 6:**
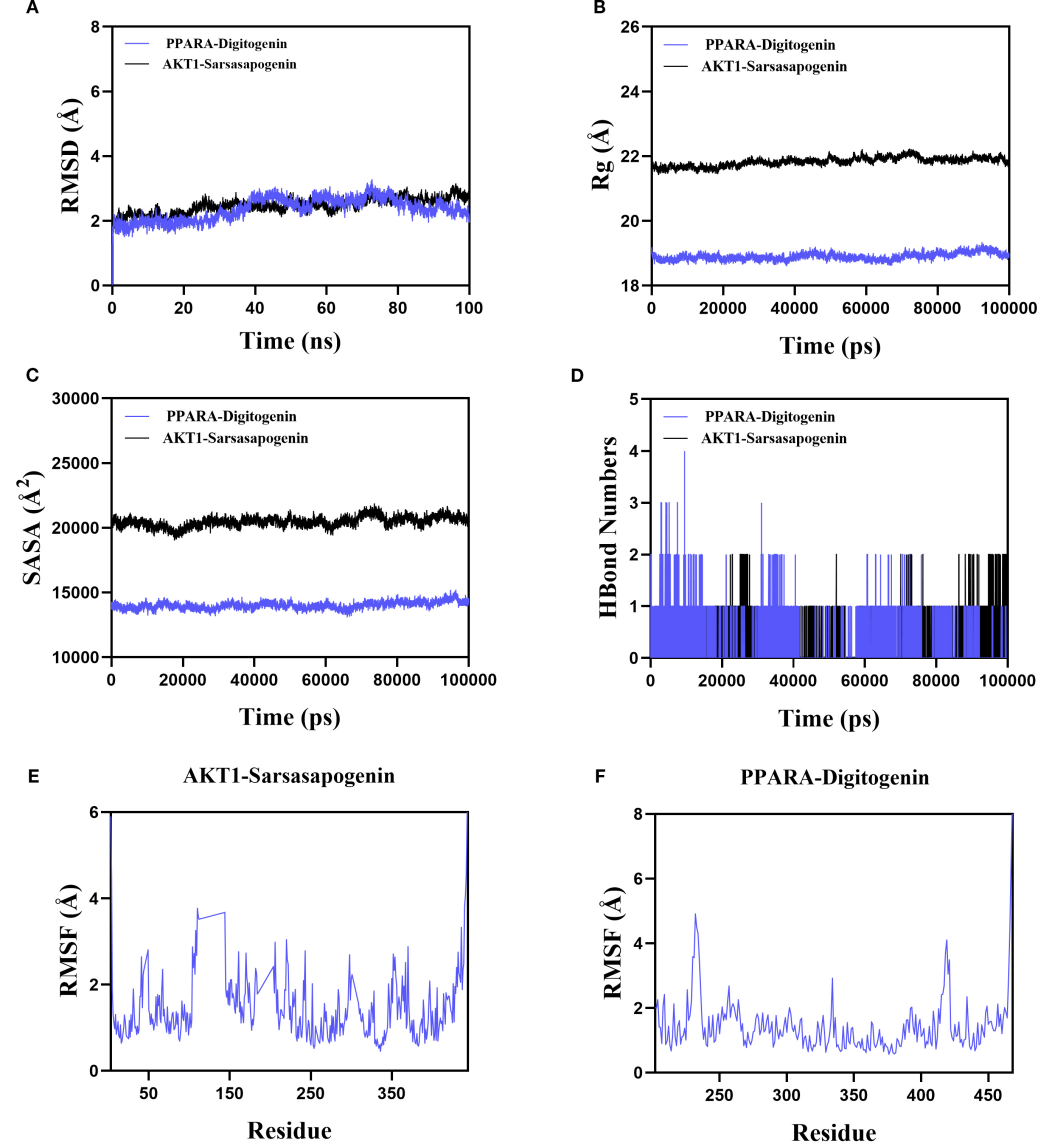
The results of MD simulation of PPARA-digitogenin and AKT1-sarsasapogenin. **(A)** RMSD. **(B)** Rg. **(C)** SASA analysis. **(D)** H bond of PPARA-digitogenin and AKT1-sarsasapogenin complex. **(E, F)** RMSF.

The RMSD serves as a reliable metric for assessing the conformational stability of protein-ligand complexes and atomic position deviations from their initial coordinates. Lower RMSD values indicate greater conformational stability. Our simulations demonstrated that the PPARA-digitogenin and AKT1-sarsasapogenin complexes reached equilibrium after 10 ns, maintaining stable RMSD fluctuations of approximately 2.7 Å and 2.5 Å, respectively (**Figure 6A**). These results confirm the high stability of digitogenin and sarsasapogenin when bound to their respective target proteins. Further analysis of the Rg and SASA (**Figures 6B, C**), indicating no significant structural contraction or expansion in either complex system during molecular dynamics.

Hydrogen bonds play a crucial role in ligand-protein binding. **Figure 6D** displays the number of hydrogen bonds formed between the small molecules and target proteins during the kinetic process. In the PPARA-digitogenin complex system, the number of hydrogen bonds fluctuated between 0 and 4, with one bond present in most cases. For the AKT1-sarsasapogenin system, the range was narrower (0 to 2), but again, one bond typically formed. These results suggest that the PPARA-digitogenin and AKT1-sarsasapogenin systems exhibit stable hydrogen-bonding interactions.

The RMSF reflects the flexibility of amino acid residues within a protein. As shown in **Figures 6E, F**, the RMSF values for the PPARA-digitogenin and AKT1-sarsasapogenin complexes remain relatively low, predominantly below 3 Å. These results indicate that the systems exhibit reduced flexibility and enhanced structural stability.

In summary, the PPARA-digitogenin and AKT1-sarsasapogenin complexes exhibit stable binding, supported by favorable hydrogen-bonding interactions. MD simulation results further validate the findings of the molecular docking. These observations provide robust evidence that PPARA, AKT1, IL1B, IL6, and NFKB1 are likely molecular targets of AR’s bioactive constituents, which contribute to its therapeutic potential. Notably, these targets associate with the PI3K-Akt, PPAR, and NF-κB signalling pathways, clarifying AR’s therapeutic mechanisms in T2DM treatment.

## 4 Discussion

In this study, we detected 47 prototype constituents and 11 metabolites of AR in blood and urine samples. Network pharmacology analysis further identified mangiferin, norathyriol, digitogenin, markogenin/neogitogenin, and sarsasapogenin as the key bioactive components of AR for T2DM treatment. A PPI network analysis uncovered 20 hub targets with high degree centrality, including TP53, AKT1, STAT3, TNF, SRC, HSP90AA1, IL6, CTNNB1, NFKB1, ESR1, RELA, MAPK1, BCL2, HRAS, MAPK3, IL1B, EGFR, PIK3CA, IFNG, and CASP3. These targets are predominantly associated with lipid metabolism, atherosclerosis, and diabetic complication pathways. Our findings indicate that AR mediates its anti-T2DM effects through multi-component regulation of critical metabolic and inflammatory signalling hubs, with particular importance placed on the PI3K-Akt and AGE-RAGE pathways as key therapeutic nodes.

AR-derived saponins demonstrate poor oral absorption and low bioavailability, requiring metabolic conversion by intestinal microbiota to produce pharmacologically active metabolites. Following oral administration, Timosaponin AIII and BII undergo rapid microbial biotransformation into sarsasapogenin, whose plasma concentration shows significant time-dependent accumulation (11). Both *in vitro* and *in vivo* studies indicate that sarsasapogenin exhibits superior anti-inflammatory activity compared to its parent compounds (12). Yu et al. proved that sarsasapogenin reduces high-fat diet-induced IR and adipose tissue inflammation by inhibiting IKK/NF-κB and c-Jun N-terminal kinase (JNK) pathways (13). As a key inflammatory mediator, activated PPARγ inhibits NF-κB transcriptional activity, which suppresses pro-inflammatory cytokines release. Zhang et al. reported that sarsasapogenin upregulated hippocampal PPARγ, p-GSK3β, and p-AKT levels in diabetic rats (14). Our results show that AR increases PPARγ protein expression in adipose tissue, thereby improving IR.

Mangiferin presents a similar metabolic pattern, with its deglycosylated metabolite norathyriol showing dramatically improved bioavailability (30.4% vs mangiferin’s 1.2%) (15). Mechanistic studies reveal that norathyriol demonstrates: Enhanced α-glucosidase inhibitory activity relative to mangiferin (16), improved glucose homeostasis through AMPK phosphorylation upregulation and increased insulin sensitivity via PTP1B inhibition (17). Furthermore, norathyriol demonstrates significantly greater efficacy than mangiferin in regulating lipid metabolism (18). Importantly, spirostanol saponins and norathyriol demonstrate superior drug-lead potential relative to their parent compounds, owing to their enhanced bioavailability and more potent hypoglycaemic and lipid-lowering activities.

Metabolomics analysis identified 33 differential metabolites associated with AR treatment in T2DM rats. Pathway enrichment analysis (FDR < 0.05, impact > 0.1) demonstrated significant modulation of linoleic acid metabolism, arachidonic acid metabolism, cytochrome P450-dependent drug metabolism, retinol metabolism, and xenobiotic metabolism by cytochrome P450.

In the analysis of differential metabolites, serum bile acid levels were significantly elevated in T2DM model rats, consistent with findings from multiple studies (19, 20). AR intervention markedly reduced these levels. Research suggests that glucose and insulin induce CYP7A1 gene expression, promoting bile acid synthesis and increasing circulating bile acid concentrations (21). As key signalling molecules in lipid and glucose metabolism, bile acids influence T2DM progression through multiple mechanisms, including the regulation of hepatic glycogen synthesis, gluconeogenesis, peripheral insulin sensitivity, and inflammation. Furthermore, bile acids modulate insulin secretion and intestinal GLP-1 production via Farnesoid X receptor (FXR) and Takeda G protein-coupled receptor 5 (TGR5) signalling pathways (22), underscoring their critical role in maintaining glucose homeostasis and adipocyte energy metabolism.

Under conditions of glucose and lipid metabolic dysregulation, plasma levels of LysoPC are significantly reduced. LysoPC plays a critical role in glucose-mediated insulin secretion and peripheral tissue insulin sensitivity. In this study, 14 LysoPC species exhibited significant decreased levels in the dysregulated state, which were markedly restored by AR treatment. This finding consistent with our prior research in diabetic human populations and animal models (4, 23).

13(S)-HPODE and 12,13-DHOME are lipoxygenase-catalysed peroxidation products of linoleic acid (LA). In T2DM model rats, enhanced lipid peroxidation leads to significant elevations in circulating 13(S)-HPODE and 12,13-DHOME levels (24). These peroxides are closely associated with inflammation, as they activate the NF-κB pathway and upregulate pro-inflammatory cytokines (including NLRP3 inflammasome components, TNF-α, IL-6, IL-1β, MCP-1) (25, 26). Metabolomic analyses demonstrated that AR and ROG interventions significantly reduced levels of these LA-derived peroxides. Consistent with pharmacological experimental data, these findings indicate that AR alleviates T2DM-associated inflammation and insulin resistance (IR) by inhibiting lipid peroxidation and suppressing NF-κB-mediated pro-inflammatory cytokine expression.

Research has demonstrated that serum free fatty acid (FFA) levels are significantly elevated in T2DM patients compared to healthy individuals. Elevated FFAs are strongly associated with multiple metabolic disorders, including inflammation, obesity, IR, atherosclerosis, and non-alcoholic fatty liver disease (NAFLD). However, not all fatty acids (FAs) exhibit lipotoxic effects: while saturated fatty acids (SFAs) such as stearic acid (SA) and palmitic acid (PA) promote IR and contribute to T2DM progression, monounsaturated fatty acids demonstrate protective properties. Plasma SFAs impair cellular homeostasis by disrupting endoplasmic reticulum (ER) and mitochondrial function, thereby inducing ER stress, generating reactive oxygen species (ROS), and activating the TLR4 signalling pathway, which collectively exacerbate inflammatory responses (27, 28). In contrast, OA—a monounsaturated fatty acid (MUFA)—counteracts SFA-induced inflammation by suppressing production of inflammatory factors (29). Mechanistically, OA restores AMPK activity, ameliorating ER and mitochondrial dysfunction while attenuating inflammatory pathways (30). Furthermore, OA enhances insulin sensitivity through modulation of the IRS1/PI3K signalling pathway (31).

A diet rich in ω-3 polyunsaturated fatty acids (PUFAs) - particularly α-linolenic acid (ALA), docosahexaenoic acid (DHA), eicosapentaenoic acid (EPA), and docosapentaenoic acid (DPA) - is well-established for its protective effects against T2DM and cardiovascular diseases (32, 33). Clinical evidence from a randomised controlled trial in Asian populations confirms that ω-3 PUFA supplementation improves plasma TG levels in T2DM patients (34). Studies indicated that with T2DM and advanced glycation (HbA1c 9.1–15%), plasma levels of SFAs - particularly PA and SA - are significantly elevated, with SA demonstrating a positive correlation with HbA1c. In contrast, as glycation levels rise (HbA1c 6–15%), levels of MUFAs (e.g., OA) and PUFAs (e.g., LA, EPA) decline markedly, with LA showing a negative correlation with HbA1c (35). Mechanistically, ω-3 PUFAs reduce obesity-associated inflammation by suppressing the release of inflammatory factors and ameliorating chronic inflammation-induced IR (36). Multiple studies demonstrate their hypoglycaemic and lipid-lowering effects, which arise through several pathways: upregulation GLP-1R expression to stimulate insulin synthesis and secretion, enhancing antioxidant defences (e.g., increased SOD activity), promoting pancreatic β-cell proliferation, inhibiting of β-cell apoptosis, and elevation adiponectin levels in T2DM patients (37, 38). Our results indicate that AR ameliorates fatty acid metabolic disorders in T2DM by normalising levels of multiple UFAs.

Joint pathway analysis pinpointed five core targets: PPARA, NFKB1, IL6, AKT1, and IL1B. The PPAR, PI3K-Akt, and NF-κB signalling pathways - which directly interact with these key targets - emerged as critical regulators of T2DM pathological progression.

Peroxisome proliferator-activated receptor alpha (PPARA) serves as a master regulator of glucose and lipid metabolism, coordinating energy homeostasis and inflammatory responses. It achieves this by inhibiting glycolysis and lipid synthesis while simultaneously enhancing glucose uptake, glycogen synthesis, and fatty acid β-oxidation (39). Endogenous PPARA ligands - including LA, OA, and ALA - activate the receptor to stimulate fatty acid β-oxidation, accelerate lipid catabolism, reduce adipose accumulation, and improve IR. Yang et al. reported that LA significantly increased the mRNA translation level of PPARA in AML12 cells, a type of hepatic parenchymal cells (40). PPARA activation also increases HDL-C levels and upregulates lipoprotein lipase (LPL) expression, thereby promoting triglyceride (TG) hydrolysis (41). PPARA activation potently inhibits NF-κB-mediated inflammation. Notably, PPARA exerts potent anti-inflammatory effects through NF-κB inhibition. The receptor directly interacts with the p65 subunit of NF-κB, blocking its nuclear translocation and subsequent transcriptional activation of pro-inflammatory genes (including IL-6, IL-1β, and TNF-α).

The PI3K-Akt signalling pathway acts as a central regulator in diabetes, cardiovascular disease, obesity, and other metabolic disorders, controlling glucose homeostasis and lipid metabolism. Phosphatidylinositol 3-kinase (PI3K) catalyses the phosphorylation of phosphatidylinositol (PI) to produce phosphatidylinositol 3,4,5-trisphosphate (PIP3), which triggers AKT1 phosphorylation at Thr308 and Ser473 (42). Activated AKT1 increases glucose uptake in skeletal muscle and adipose tissues by upregulating GLUT1/GLUT4 transporters through thioredoxin-interacting protein (TXNIP) modulation (43). The pathway also plays a key role in pancreatic β-cell proliferation and survival: Akt-mediated phosphorylation of mTOR, GSK3β, and FoxO1 suppresses caspase-9-dependent apoptosis, protecting β-cell mass (44).

At physiological concentrations, LA improves insulin sensitivity through two key mechanisms: it promotes tyrosine phosphorylation of the insulin receptor (INSR), activating PI3K-PIP3 signalling, and recruits Akt1 to the plasma membrane for phosphorylation by PDK1 and mTORC2 at Thr308/Ser473 (45, 46). This enhances downstream glucose uptake via GLUT4 translocation in myocytes and adipocytes. Simultaneously, LA inhibits pro-inflammatory NF-κB pathways, reducing TNF-α-induced serine phosphorylation of insulin receptor substrate 1 (IRS-1) and alleviating its inhibitory crosstalk with insulin signalling. In endothelial and neuronal cells, LA-driven Akt1 activation further promotes cell survival by suppressing Bad-dependent apoptosis and boosting antioxidant responses, highlighting its dual role in metabolic and vascular protection.

T2DM is increasingly recognised as a chronic low-grade inflammatory disease driven by cytokines. Pro-inflammatory cytokines directly or indirectly impair pancreatic β-cell function and induce apoptosis. Joint pathway analysis identified NFKB1, IL6, and IL1B are key targets mediating the anti-inflammatory effects of AR in T2DM. The NF-κB pathway plays a central role in inflammation by promoting the release of pro-inflammatory cytokines (TNF-α, IL-6, IL-1β) and upregulating iNOS and NO upon activation (47). These mediators chemotactically recruit monocytes/macrophages to pancreatic and renal tissues, where TLR4-mediated amplification of NF-κB signalling cascades drives further inflammatory factor release. This process promotes pancreatic amyloid deposition, fibrosis, and glomerular endothelial cell damage, exacerbating organ-specific inflammation (48, 49). Under oxidative stress, accumulated inflammatory factors activate IκB kinase (IKK), which induces serine phosphorylation of INSR and IRS. This disrupts of PI3K/Akt signalling leads to IR (50). Hyperglycemia further elevates TGF-β1 levels, and NF-κB activation enhances its expression—a key mechanism driving hepatic and renal fibrosis in T2DM. In this study, AR treatment significantly reduced serum levels of TNF-α, IL-1β, and IL-6 while inhibiting hepatic NF-κB and TGF-β1 gene expression. These effects collectively alleviated inflammation and tissue fibrosis in T2DM rats.

Furthermore, joint pathway analysis further confirmed that the AGE-RAGE signalling pathway, the TNF signalling pathway, and the IL-17 signalling pathway are also key mechanisms mediating the hypoglycaemic efficacy of AR in diabetes and its complications.

Advanced glycation end products (AGEs), toxic by-products of glucose metabolism, are positively and significantly associated with the risk of developing T2DM. For every 1-standard-deviation rise in circulating AGEs, the incidence of T2DM increases by approximately 52% (51). AGEs directly impair pancreatic β-cell function and attenuate insulin secretion. Concurrently, they enhance p38 MAPK phosphorylation in intestinal GLUTag cells, activate the NF-κB pathway, and stimulate the release of pro-inflammatory cytokines (TNF-α, IL-1, and IL-6), thereby inducing cellular damage and apoptosis (52). Furthermore, the AGE-RAGE axis disrupts insulin signalling in skeletal muscle and endothelial cells, provoking inflammation, oxidative stress, and apoptosis; these effects diminish tissue glucose uptake and ultimately elevate blood glucose through exacerbated IR.

Interleukin-17 (IL-17), a pro-inflammatory cytokine produced by Th17 cells, is central to immune regulation and inflammatory responses. It shapes the inflammatory microenvironment of T2DM and disrupts glucose homeostasis via MAPK cascades. Clinical studies consistently report higher serum IL-17 levels in newly diagnosed T2DM patients (53). Engagement of the IL-17 receptor complex activates MAPK and NF-κB signalling, which rapidly induces the release of pro-inflammatory mediators such as IL-6 and TNF-α. These cytokines sustain NF-κB activation, which in turn transcriptionally up-regulates TNF-α and IL-6, thereby establishing a self-amplifying inflammatory loop (54). This chronic inflammatory state ultimately precipitates hyperglycaemia and IR. Moreover, TNF-α directly impairs insulin sensitivity by inhibiting PI3K activity and PPAR-γ function, thus contributing to T2DM pathogenesis.

These interconnected and synergistic mechanisms collectively underscore the pivotal role of AR in modulating systemic glucose homeostasis and the pathogenesis and progression of diabetes. Consequently, AR may confer therapeutic benefits against diabetes by attenuating inflammatory responses. Future studies will experimentally elucidate the precise regulatory effects of AR on the AGE-RAGE, TNF, and IL-17 signalling pathways in diabetes and its complications, thereby deepening our understanding of its efficacy and underlying mechanisms in T2DM.

We further conducted molecular docking and MD simulations of the five core components against the five core targets. The results from molecular docking revealed that sarsasapogenin, markogenin/neogitogenin, and digitogenin exhibited strong binding affinity to all five core targets, with sarsasapogenin showing the highest binding efficacy. Norathyriol formed stable complexes with four core targets (excluding NFKB1), whereas mangiferin displayed favourable binding energy with AKT1 and PPARA but weaker interactions with the remaining three targets.

MD simulations confirmed that the PPARA-digitogenin and AKT1-sarsasapogenin complexes form stable binding conformations. Both complexes reach equilibrium quickly and maintain stable fluctuations. Notably, the two complexes exhibit distinct structural characteristics: the PPARA-digitogenin complex adopts a relatively compact conformation, while the AKT1-sarsasapogenin complex has a more expanded structure with a larger SASA. This structural discrepancy may be due to variations in the domain organization of the target proteins or differences in steric compatibility at their respective ligand-binding interfaces. Throughout the simulations, no significant structural changes were observed, and hydrogen-bonding patterns remained consistent, predominantly involving single bonds. Reduced residue flexibility further indicated enhanced structural rigidity. Overall, these findings validate the stable interactions between the ligands and their targets, supporting their potential biological functionality.

**Figure 7:**
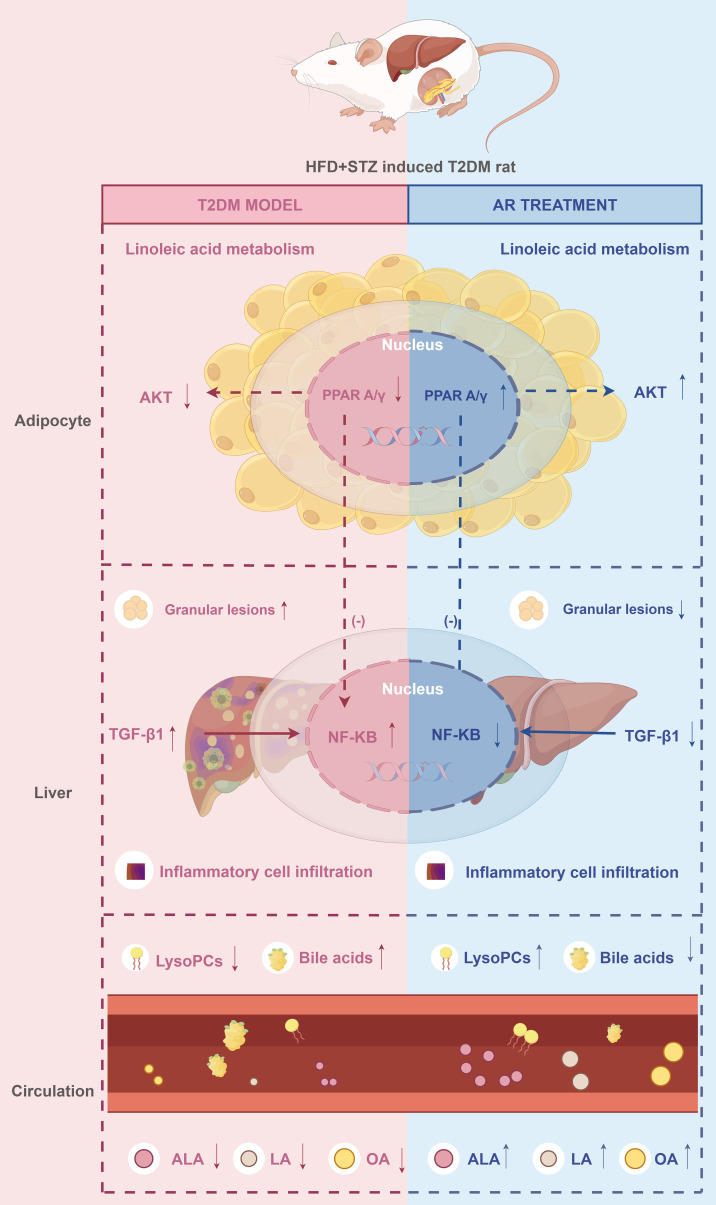
The proposed synergistic effect mechanisms of AR against T2DM. ↑: increase. ↓: decrease. Drawn by Figdraw. ALA, α-Linolenic acid; LA, Linoleic acid; OA, oleic acid.

In summary, we identified PPARA, NFKB1, IL6, AKT1, and IL1B as potential therapeutic targets of AR, suggesting that their associated signalling pathways (including PPAR, PI3K-Akt, NF-κB) mediate AR’s anti-diabetic effects. The metabolites sarsasapogenin, markogenin/neogitogenin, digitogenin and norathyriol emerged as AR’s primary hypoglycaemic components. These findings reflect the characteristic “multi-component, multi-target, multi-pathway” therapeutic approach of TCM, demonstrating how AR coordinately regulates T2DM by through integrated modulation of multiple molecular targets and signalling networks (**Figure 7**).

This study has several limitations that warrant attention. First, we conducted animal experiments in rats, and species-specific differences in metabolic enzyme activity may limit the extrapolation of these results to humans. Second, the bioavailability of active compounds (e.g., spirostanol saponins) in humans remains unclear, and further limitations include not only potential species differences but also discrepancies between the administration of AR in this study (ethanol extract via gavage) and its clinical use (aqueous decoction)—factors such as extraction solvent and route of administration may alter constituent bioavailability. This highlights the need for pharmacodynamic and pharmacokinetic studies on monomers such as sarsasapogenin to evaluate their actual therapeutic efficacy. Furthermore, although we preliminarily validated core targets identified via network pharmacology through molecular docking and MD simulations, the protein expression of the five core targets—except for NFKB1 mRNA expression in liver tissues—requires further cellular-level verification to strengthen mechanistic insights. Future experiments will address these limitations.

## 5 Conclusion

Using an integrative approach combining metabolomics, serum-urine pharmacochemistry, network pharmacology, molecular docking, MD simulations, and pharmacological experiments, this study identified spirostanol saponins and norathyriol as the primary active components of AR responsible for its anti-T2DM effects. The therapeutic action of AR arises from its regulation of five core targets (PPARA, NFKB1, IL6, AKT1, and IL1B) and six key signaling pathways (PPAR, AGE-RAGE, IR, NAFLD, TNF, and IL-17 signaling pathways), as well as its modulation of linoleic acid metabolism. These findings provide a robust foundation for understanding AR’s material basis and mechanisms of action in T2DM treatment, supporting its clinical application and offering insights for future T2DM drug development.

## Data Availability

The original contributions presented in the study are included in the article/**Supplementary Material**. Further inquiries can be directed to the corresponding authors.
