# Supplementary material for: Metabolomics integrated with network pharmacology and serum-urine pharmacochemistry unveils the antidiabetic mechanism of Anemarrhenae Rhizoma
Source: Front Endocrinol (Lausanne). 2025 Oct 9;16:1618584. doi: 10.3389/fendo.2025.1618584 (PMC12545018; doi:10.3389/fendo.2025.1618584)
Supplement: Supplementary file 1 [file Table1.docx]

Supplementary Material

# Supplementary Tables

1.1 Supplementary Table S1. Primer Information

| **Gene Name** | **Primer sequence** | **PCR fragment (bp)** |
| --- | --- | --- |
| β-actin | F：5' -GAGGGAAATCGTGCGTGAC- 3'  R：5' -TAGGAGCCAGGGCAGTAATCT-3' | 353 |
| TGF-β1 | F：5'-CGGCAGCTGTACATTGACTT-3'  R：5'-TCAGCTGCACTTGCAGGAGC-3' | 282 |
| NF-kB(P65) | F：5'-ATCTGTTTCCCCTCATCTTTCC-3'  R：5'-TGGGTGCGTCTTAGTGGTATCT-3' | 170 |

1.2 Supplementary Table S2. The gradient elution conditions of AR constituents identification *in vivo*

| Time ( min ) | Flow rate (mL/min) | Water with 0.1% formic acid (A%) | Acetonitrile (B%) | curve |
| --- | --- | --- | --- | --- |
| 0 | 0.4 | 95 | 5 | 6 |
| 3 | 0.4 | 85 | 15 | 6 |
| 8 | 0.4 | 80 | 20 | 6 |
| 12 | 0.4 | 78 | 22 | 6 |
| 22 | 0.4 | 73 | 27 | 6 |
| 27 | 0.4 | 50 | 50 | 6 |
| 30 | 0.4 | 0 | 100 | 6 |

Notes: the injection volume was 10 µL

1.3 Supplementary Table S3. The gradient elution conditions of serum metabolomic analysis

| Time ( min ) | Flow rate (mL/min) | Water with 0.1% formic acid (A%) | Acetonitrile with 0.1% formic acid (B%) | curve |
| --- | --- | --- | --- | --- |
| 0 | 0.4 | 95 | 5 | 6 |
| 5 | 0.4 | 70 | 30 | 6 |
| 7 | 0.4 | 60 | 40 | 6 |
| 9 | 0.4 | 60 | 40 | 6 |
| 11 | 0.4 | 50 | 50 | 6 |
| 18 | 0.4 | 20 | 80 | 6 |
| 19 | 0.4 | 0 | 100 | 6 |
| 20 | 0.4 | 95 | 5 | 6 |
| 22 | 0.4 | 95 | 5 | 6 |

Notes: the injection volume was 10 µL

**1.4 Supplementary Table S4. Post hoc comparisons of pharmacodynamic parameters among experimental groups**

| Index | Multiple comparisons | NC vs. DM | DM vs. ROG | DM vs. ARH | DM vs. ARM | DM vs. ARL |
| --- | --- | --- | --- | --- | --- | --- |
| FBG | Mean Diff. | -19.45 | 5.313 | 6.833 | 3.49 | 1.15 |
|  | 95%CI of diff. | -22.81 to -16.09 | 1.789 to 8.838 | 3.595 to 10.07 | 0.2524 to 6.729 | -2.210 to 4.510 |
|  | Adjusted P Value | ＜0.0001 | 0.0009 | ＜0.0001 | 0.0287 | 0.9011 |
| FINS | Mean Diff. | -14.86 | 10.49 | 13.59 | 11.44 | 8.26 |
|  | 95%CI of diff. | -17.75 to -11.97 | 7.462 to 13.53 | 10.80 to 16.38 | 8.655 to 14.23 | 5.368 to 11.15 |
|  | Adjusted P Value | ＜0.0001 | ＜0.0001 | ＜0.0001 | ＜0.0001 | ＜0.0001 |
| TC | Mean Diff. | -2.483 | 1.177 | 2.139 | 1.236 | 1.022 |
|  | 95%CI of diff. | -2.928 to -2.039 | 0.7111 to 1.644 | 1.711 to 2.567 | 0.8078 to 1.665 | 0.5771 to 1.466 |
|  | Adjusted P Value | ＜0.0001 | ＜0.0001 | ＜0.0001 | ＜0.0001 | ＜0.0001 |
| TG | Mean Diff. | -0.9300 | 0.4167 | 0.7924 | 0.4681 | 0.3317 |
|  | 95%CI of diff. | -1.104 to -0.7559 | 0.2340 to 0.5993 | 0.6246 to 0.9602 | 0.3003 to 0.6359 | 0.1575 to 0.5058 |
|  | Adjusted P Value | ＜0.0001 | ＜0.0001 | ＜0.0001 | ＜0.0001 | ＜0.0001 |
| LDL-C | Mean Diff. | -1.142 | 0.6240 | 0.9686 | 0.8286 | 0.5133 |
|  | 95%CI of diff. | -1.331 to -0.9524 | 0.4255 to 0.8225 | 0.7862 to 1.151 | 0.6462 to 1.011 | 0.3241 to 0.7026 |
|  | Adjusted P Value | ＜0.0001 | ＜0.0001 | ＜0.0001 | ＜0.0001 | ＜0.0001 |
| HDL-C | Mean Diff. | 0.8117 | -0.5420 | -0.6314 | -0.4457 | -0.3750 |
|  | 95%CI of diff. | 0.6554 to 0.9679 | -0.7058 to -0.3782 | -0.7820 to -0.4809 | -0.5963 to -0.2952 | -0.5312 to -0.2188 |
|  | Adjusted P Value | ＜0.0001 | ＜0.0001 | ＜0.0001 | ＜0.0001 | ＜0.0001 |
| TNF-α | Mean Diff. | -104.8 | 49.68 | 61.69 | 39.25 | 14.68 |
|  | 95%CI of diff. | -139.1 to -70.54 | 14.24 to 85.11 | 25.53 to 97.86 | 1.222 to 77.28 | -23.34 to 52.71 |
|  | Adjusted P Value | ＜0.0001 | 0.0016 | ＜0.0001 | 0.0392 | 0.8637 |
| IL-6 | Mean Diff. | -92.34 | 33.29 | 41.12 | 34.94 | 18.32 |
|  | 95%CI of diff. | -130.4 to -54.29 | -4.750 to 71.33 | 3.083 to 79.17 | -3.099 to 72.98 | -19.72 to 56.37 |
|  | Adjusted P Value | ＜0.0001 | 0.1108 | 0.0288 | 0.0848 | 0.6739 |
| IL-1β | Mean Diff. | -126.5 | 106.9 | 117.3 | 78.86 | 32.59 |
|  | 95%CI of diff. | -174.3 to -78.72 | 59.13 to 154.7 | 69.55 to 165.1 | 31.07 to 126.7 | -15.20 to 80.39 |
|  | Adjusted P Value | ＜0.0001 | ＜0.0001 | ＜0.0001 | 0.0003 | 0.3272 |

Data were analyzed using one-way ANOVA and Tukey’s HSD post hoc test, with statistical significance defined as p < 0.05. Results are presented as mean difference [95% confidence interval (CI)] and adjusted p-values for pairwise comparisons.

1.5 Supplementary **Table S5.** The MS and MS/MS data of chemical constituents from AR, as well as the prototype constituents in rat serum and urine after oral administration of AR, were analyzed in both positive and negative ion modes.

| **No** | **t_R_/min** | **Ion mode** | **Mass(*m/z*)** | | **Error**  **(ppm)** | **Molecular**  **formula** | **Identification** | **MS/MS fragment ions (*m/z*)** | **Observed** | |
| --- | --- | --- | --- | --- | --- | --- | --- | --- | --- | --- |
|  |  |  | **Calculated** | **Measured** |  |  |  |  | **Serum** | **Urine** |
| 1^*^ | 1.83 | [M+H]^+^ | 585.1456 | 585.1456 | 0.0 | C_25_H_28_O_16_ | Neomangiferin | 567.1329, 549.1257, 519.1105, 489.0994, 465.1039, 435.0957, 405.0806, 387.0746, 369.0598, 357.0589, 351.0495, 339.0492, 327.0567, 303.0557, 273.0331, 261.0388 | **√** |  |
|  |  | [M-H]^-^ | 583.1299 | 583.1273 | -4.5 |  |  | 565.1187, 493.0993, 463.0906, 421.0738, 403.0624, 331.0436, 301.0324, 259.0216 |  |  |
| 2 | 2.03 | [M+H]+ | 567.1350 | 567.1344 | -1.1 | C_25_H_26_O_15_ | Dehydration product of neomangiferin | 405.0800, 387.0733, 369.0635, 357.0594, 351.0546, 339.0556,273.0362, 261.0381 |  |  |
|  |  | [M-H]^-^ | 565.1193 | 565.1205 | 2.1 |  |  | 403.0669, 331.0468, 301.0369, 271.0235, 259.0253 |  |  |
| 3 | 2.51 | [M+H]+ | 423.0927 | 423.0939 | 2.8 | C_19_H_18_O_11_ | Mangiferin | 405.0796, 387.0709, 369.0613, 351.0488, 339.0475, 327.0549, 303.0550, 299.0555, 285.0418, 273.0405, 261.0382 | **√** | **√** |
|  |  | [M-H]^-^ | 421.0771 | 421.0784 | 3.1 |  |  | 403.0648, 385.0524, 355.0445, 343.0434, 331.0461, 313.0362, 301.0359, 285.0399, 271.0275, 259.0226 |  |  |
| 4 | 3.10 | [M+H]+ | 1115.2516 | 1115.2501 | -1.3 | C_50_H_50_O_29_ | Dehydrated dimer of neomangiferin | 953.1985, 585.1469, 423.0948 |  |  |
|  |  | [M-H]^-^ | 1113.2360 | 1113.2365 | 0.4 |  |  | 951.2011, 583.1317, 421.0810 |  |  |
| 5 | 3.41 | [M+H]+ | 585.1456 | 585.1476 | 3.4 | C_25_H_28_O_16_ | Isomer of neomangiferin | 567.1343, 549.1243, 519.1098, 489.0987, 465.1034, 435.0885, 405.0804, 387.0776, 369.0628, 357.0595, 351.0489, 339.0502, 327.0507, 303.0499, 273.0345, 261.0386 |  |  |
|  |  | [M-H]^-^ | 583.1299 | 583.1287 | -2.1 |  |  | 565.1183, 493.1001, 463.0856, 421.0745, 403.0627, 343.0422, 331.0464, 301.0336, 259.0203 |  |  |
| 6 | 3.56 | [M+H]+ | 1115.2516 | 1115.2478 | -3.4 | C_50_H_50_O_29_ | Dehydrated dimer of neomangiferin | 953.2064, 585.1476, 423.0939 |  |  |
|  |  | [M-H]^-^ | 1113.2360 | 1113.2377 | 1.5 |  |  | 951.2023, 583.1308, 421.0757 |  |  |
| 7 | 3.65 | [M+H]+ | 423.0927 | 423.0930 | 0.7 | C_19_H_18_O_11_ | Isomer of mangiferin | 387.0776, 369.0634, 351.0495, 339.0492, 327.0567, 303.0527, 299.0577, 285.0381, 273.0381, 257.0433 |  | **√** |
|  |  | [M-H]^-^ | 421.0771 | 421.0778 | 1.7 |  |  | 403.0641, 355.0435, 331.0451, 313.0362, 301.0309, 285.0360, 271.0217, 259.0266 |  |  |
| 8 | 3.86 | [M+H-H_2_O]^+^ | 919.4903 | 919.4925 | 2.4 | C_45_H_76_O_20_ | 27OH-timosaponin BⅡ | 757.4354, 595.3858, 433.3326, 415.3202, 289.2177, 273.2249, 255.2138 |  |  |
|  |  | [M-H]^-^ | 935.4852 | 935.4809 | -4.6 |  |  | 917.4755, 773.4393, 611.3427, 533.1269, 516.1101, 431.0987, 421.0722, 403.0699 |  |  |
| 9 | 3.98 | [M+H-H_2_O]^+^ | 919.4903 | 919.4860 | -4.7 | C_45_H_76_O_20_ | 25*S*-27OH-timosaponin BⅡ | 757.4338, 595.3867, 433.3306, 415.3214, 289.2187, 273.2234, 253.2113 |  |  |
|  |  | [M-H]^-^ | 935.4852 | 935.4828 | -2.6 |  |  | 917.4791, 773.4427, 611.3481, 533.1388, 516.1219, 431.0993, 421.0728, 403.0706 |  |  |
| 10 | 4.35 | [M+H]+ | 953.4957 | 953.4915 | -4.4 | C_45_H_76_O_21_ | 15OH-timosaponin N or its isomer | 935.4876, 773.4315, 755.4256, 611.3809, 593.3644, 431.3145, 413.3016, 287.2025, 271.2168, 251.1889 |  |  |
|  |  | [M-H]^-^ | 951.4801 | 951.4783 | -1.9 |  |  | 789.4295, 627.3767, 465.3254 |  |  |
| 11 | 4.41 | [M+H]+ | 423.0927 | 423.0939 | 2.8 | C_19_H_18_O_11_ | Isomer of mangiferin | 387.0755, 369.0586, 351.0539, 339.0541, 327.0511, 303.0535, 299.0548, 285.0415, 273.0358, 257.0497 |  |  |
|  |  | [M-H]^-^ | 421.0771 | 421.0778 | 1.7 |  |  | 403.0634, 385.0525, 355.0435, 331.0422, 313.0353, 301.0375, 285.0383, 271.0254, 259.0237 |  |  |
| 12 | 4.92 | [M+H-H_2_O]^+^ | 1213.5853 | 1213.5890 | 3.0 | C_56_H_94_O_29_ | Isomer of 25*S*-purpureagitosid | 1213.5822, 919.4869, 757.4396, 595.3735, 433.3373, 415.2974, 271.1017 |  |  |
|  |  | [M-H]^-^ | 1229.5803 | 1229.5820 | 1.4 |  |  | 1097.5392, 935.4822, 773.4458, 449.3247 |  |  |
| 13 | 5.24 | [M+H]+ | 953.4957 | 953.4968 | 1.2 | C_45_H_76_O_21_ | 15OH-timosaponin N or its isomer | 935.4833, 773.4349, 755.4205, 611.3785, 593.3679, 431.3155, 413.3067, 287.2069, 271.2067, 251.1919 |  |  |
|  |  | [M-H]^-^ | 951.4801 | 951.4844 | 4.5 |  |  | 789.4248, 627.3739, 465.3278 |  |  |
| 14 | 5.49 | [M+H]+ | 937.5008 | 937.5016 | 0.9 | C_45_H_76_O_20_ | Isomer of timosaponin N | 757.4397, 595.3824, 433.3353, 415.3213, 289.2183, 271.2122 |  |  |
|  |  | [M-H]^-^ | 935.4852 | 935.4866 | 1.5 |  |  | 917.4742, 773.4305, 755.4263, 611.3830 |  |  |
| 15 | 5.70 | [M+NH_4_]^+^ | 952.5117 | 952.5159 | 4.4 | C_45_H_74_O_20_ | 25(27)-ene-timosaponin N or its isomer | 935.4817, 917.4694, 755.4246, 593.3707, 431.3181 |  |  |
|  |  | [M-H]^-^ | 933.4695 | 933.4675 | -2.1 |  |  | 771.4561, 609.3925 |  |  |
| 16 | 5.87 | [M+H-H_2_O]^+^ | 935.4852 | 935.4847 | -0.5 | C_45_H_76_O_21_ | 15OH-macrostemonoside J | 773.4351, 755.4227, 611.3814, 593.3708, 431.3138, 413.3093, 287.1998, 271.2112, 251.1776 | **√** |  |
|  |  | [M-H]^-^ | 951.4801 | 951.4844 | 4.5 |  |  | 789.4281, 627.3789, 465.3186 |  |  |
| 17 | 6.46 | [M+NH_4_]^+^ | 954.5274 | 954.5232 | -4.4 | C_45_H_76_O_20_ | Macrostemonoside J | 757.4366, 595.3861, 433.3338, 415.3215, 397.3117, 289.2170, 271.2051, 253.1876 | **√** |  |
|  |  | [M-H]^-^ | 935.4852 | 935.4866 | 1.5 |  |  | 917.4756, 773.4361, 755.4279, 611.3746 |  |  |
| 18 | 6.76 | [M+H-H_2_O]^+^ | 917.4746 | 917.4765 | 2.1 | C_45_H_74_O_20_ | 25(27)-ene-timosaponin N or its isomer | 755.4239, 593.3744, 431.3208, 413.3100 |  |  |
|  |  | [M-H]^-^ | 933.4695 | 933.4725 | 3.2 |  |  | 771.4562, 609.4068 |  |  |
| 19 | 7.10 | [M+H-H_2_O]^+^ | 919.4903 | 919.4908 | 0.5 | C_45_H_76_O_20_ | Timosaponin N | 757.4369, 595.3826, 433.3324, 415.3241, 289.2158, 271.2063, 253.1997 | **√** | **√** |
|  |  | [M-H]^-^ | 935.4852 | 935.4874 | 2.4 |  |  | 917.4750, 773.4355, 755.4234, 611.3807 |  |  |
| 20 | 7.58 | [M+H-H_2_O]^+^ | 1241.6166 | 1241.6135 | -2.5 | C_58_H_98_O_29_ | Timosaponin BⅥ | 1079.5594, 917.5035, 755.4624, 737.4466, 593.3969, 575.3960, 413.3019 |  |  |
|  |  | [M-H]^-^ | 1257.6116 | 1257.6104 | -1.0 |  |  | 1095.5561, 933.5084, 915.4962, 591.3790 |  |  |
| 21 | 7.73 | [M+NH_4_]^+^ | 936.5168 | 936.5181 | 1.4 | C_45_H_74_O_19_ | Timosaponin M | 919.5037, 757.4350, 595.3915, 433.3379, 415.3215, 289.2157, 271.2076 | **√** | **√** |
|  |  | [M-H]^-^ | 917.4746 | 917.4756 | 1.1 |  |  | 755.4229, 593.3645 |  |  |
| 22 | 7.90 | [M+H-H_2_O]^+^ | 1211.5697 | 1211.5671 | -2.1 | C_56_H_92_O_29_ | Karatavioside C | 917.4784, 755.4324, 593.3769, 431.3324, 413.3019 | **√** |  |
|  |  | [M-H]^-^ | 1227.5646 | 1227.5673 | 2.2 |  |  | 1095.5304, 933.4655, 771.4123, 609.3619, 447.3150 |  |  |
| 23 | 7.96 | [M+NH_4_]^+^ | 1248.6225 | 1248.6188 | -3.0 | C_56_H_94_O_29_ | Purpureagitosid | 1213.5801, 919.4835, 901.4904, 757.4390, 739.4255, 595.3809, 577.3728, 433.3349, 415.3215, 397.3122, 271.0970 | **√** |  |
|  |  | [M-H]^-^ | 1229.5803 | 1229.5784 | -1.5 |  |  | 1097.5411, 935.4896, 773.4463, 611.3789, 449.3277 |  |  |
| 24 | 8.13 | [M+NH_4_]^+^ | 1248.6225 | 1248.6162 | -5.0 | C_56_H_94_O_29_ | 25*S*-purpureagitosid | 1213.5851, 919.4846, 901.4867, 757.4225, 739.4357, 595.3903, 577.3938, 433.3333, 415.3297, 397.3203, 301.1391 | **√** |  |
|  |  | [M-H]^-^ | 1229.5803 | 1229.5758 | -3.7 |  |  | 1097.5402, 935.4868, 773.4458, 611.3815, 449.3269 |  |  |
| 25 | 8.29 | [M+H-H_2_O]^+^ | 917.4746 | 917.4707 | -4.3 | C_45_H_74_O_20_ | Timosaponin S or its isomer | 755.4224, 593.3650, 431.3124, 413.3019 |  |  |
|  |  | [M-H]^-^ | 933.4695 | 933.4680 | -1.6 |  |  | 771.4694, 609.3774 |  |  |
| 26 | 8.46 | [M+NH_4_]^+^ | 954.5274 | 954.5302 | 2.9 | C_45_H_76_O_20_ | Timosaponin E_1_ | 757.4354, 595.3849, 433.3326, 289.2230, 271.2067, 253.2013 | **√** | **√** |
|  |  | [M-H]^-^ | 935.4852 | 935.4886 | 3.6 |  |  | 917.4769, 733.4309, 755.4263, 611.3823 |  |  |
| 27 | 8.61 | [M+H-H_2_O]^+^ | 1065.5482 | 1065.5508 | 2.4 | C_51_H_86_O_24_ | Asparagoside G | 903.4953, 741.4376, 579.3907, 417.3393, 399.3300, 273.2247, 255.2172 | **√** | **√** |
|  |  | [M-H]^-^ | 1081.5431 | 1081.5485 | 5.0 |  |  | 919.4946, 757.4409, 595.3827 |  |  |
| 28 | 8.94 | [M+H-H_2_O]^+^ | 919.4903 | 919.4916 | 1.4 | C_45_H_76_O_20_ | 25*R*-timosaponin E_1_ | 757.4403, 595.3819, 433.3334, 415.3220, 289.2242, 271.2098, 253.2016 | **√** | **√** |
|  |  | [M-H]^-^ | 935.4852 | 935.4809 | -4.6 |  |  | 917.4755, 773.4316, 755.4237, 611.3808 |  |  |
| 29 | 9.11 | [M+H-H_2_O]^+^ | 1065.5482 | 1065.5526 | 4.1 | C_51_H_86_O_24_ | Tomatoside A | 903.4942, 741.4448, 579.3935, 417.3361, 399.3257, 273.2229, 255.2126 | **√** |  |
|  |  | [M-H]^-^ | 1081.5431 | 1081.5420 | -1.0 |  |  | 919.4913, 757.4367, 595.3869 |  |  |
| 30 | 9.27 | [M+NH_4_]^+^ | 952.5117 | 952.5157 | 4.2 | C_45_H_74_O_20_ | Timosaponin S or its isomer | 935.4794, 917.4769, 755.4308, 593.3638, 431.3217, 413.3010 |  |  |
|  |  | [M-H]^-^ | 933.4695 | 933.4742 | 5.0 |  |  | 771.4662, 609.3900 |  |  |
| 31 | 9.37 | [M+H-H_2_O]^+^ | 1065.5482 | 1065.5526 | 4.1 | C_51_H_86_O_24_ | Petunioside N | 903.4927, 741.4452, 579.3915, 417.3435, 399.3250, 273.2195, 255.2121 |  | **√** |
|  |  | [M-H]^-^ | 1081.5431 | 1081.5398 | -3.1 |  |  | 919.4957, 757.4339, 595.3888 |  |  |
| 32 | 9.62 | [M+H-H_2_O]^+^ | 1065.5482 | 1065.5526 | 4.1 | C_51_H_86_O_24_ | 25*R*-petunioside N | 903.5012, 741.4486, 579.3834, 417.3336, 399.3247, 273.2275, 255.2176 |  |  |
|  |  | [M-H]^-^ | 1081.5431 | 1081.5398 | -3.1 |  |  | 919.4947, 757.4363, 595.3834 |  |  |
| 33 | 9.95 | [M+H-H_2_O]^+^ | 901.4797 | 901.4759 | -4.2 | C_45_H_74_O_19_ | Timosaponin L | 919.4956, 757.4347, 595.3876, 433.3363, 415.3237, 289.2239, 271.2116 | **√** | **√** |
|  |  | [M-H]^-^ | 917.4746 | 919.4706 | -4.4 |  |  | 755.4196, 593.3678 |  |  |
| 34 | 10.05 | [M+H-H_2_O]^+^ | 1065.5482 | 1065.5504 | 2.1 | C_51_H_86_O_24_ | 27OH-timosaponin BⅡ+Rha | 903.5029, 741.4467, 579.3846, 417.3344, 399.3255, 273.2281, 255.2203 |  | **√** |
|  |  | [M-H]^-^ | 1081.5431 | 1081.5448 | 1.6 |  |  | 919.4955, 757.4352, 595.3820 |  |  |
| 35 | 10.34 | [M+H-H_2_O]^+^ | 903.4953 | 903.4942 | -1.2 | C_45_H_76_O_19_ | 25*R*-timosaponin BⅡ | 741.4446, 579.3852, 417.3352, 399.3242, 273.2246, 255.2109 | **√** | **√** |
|  |  | [M-H]^-^ | 919.4903 | 919.4941 | 4.1 |  |  | 757.4395, 595.3876 |  |  |
| 36^*^ | 10.72 | [M+H-H_2_O]^+^ | 903.4953 | 903.4957 | 0.4 | C_45_H_76_O_19_ | Timosaponin BⅡ | 741.4458, 579.3925, 417.3352, 399.3246, 273.2236, 255.2186 | **√** | **√** |
|  |  | [M-H]^-^ | 919.4903 | 919.4900 | -0.3 |  |  | 757.4345, 595.3850 |  |  |
| 37 | 11.09 | [M+H-H_2_O]^+^ | 903.4953 | 903.4937 | -1.8 | C_45_H_76_O_19_ | 25*S*-Officinalisinin-Ⅰ | 741.4463, 579.3957, 417.3377, 399.3267, 273.2219, 255.2126 | **√** | **√** |
|  |  | [M-H]^-^ | 919.4903 | 919.4888 | -1.5 |  |  | 757.4404, 595.3839 |  |  |
| 38 | 11.28 | [M+H-H_2_O]^+^ | 1065.5482 | 1065.5526 | 4.1 | C_51_H_86_O_24_ | 25*S*-27OH-timosaponin BⅡ+Rha | 903.5003, 741.4428, 579.3911, 417.3379, 399.3288, 273.2224, 255.2117 | **√** | **√** |
|  |  | [M-H]^-^ | 1081.5431 | 1081.5457 | 2.4 |  |  | 919.4928, 757.4373, 595.3835 |  |  |
| 39 | 11.35 | [M+H-H_2_O]^+^ | 903.4953 | 903.4914 | -4.3 | C_45_H_76_O_19_ | Officinalisinin-Ⅰ | 741.4416, 579.3935, 417.3350, 399.3240, 273.2243, 255.2134 | **√** | **√** |
|  |  | [M-H]^-^ | 919.4903 | 919.4905 | 0.2 |  |  | 757.4362, 595.3865 |  |  |
| 40 | 11.84 | [M+H-H_2_O]^+^ | 1197.5904 | 1197.5845 | -4.9 | C_56_H_94_O_28_ | Timosaponin D_1_ | 1065.5370, 903.4904, 741.4362, 579.3836, 417.3352 | **√** |  |
|  |  | [M-H]^-^ | 1213.5853 | 1213.5792 | -5.0 |  |  | 1081.5432, 1051.5387, 919.4961, 757.4357 |  |  |
| 41 | 12.05 | [M+NH_4_]^+^ | 1230.6119 | 1230.6152 | 2.7 | C_56_H_92_O_28_ | Timosaponin C_1_ | 1213.5857, 1195.5823, 901.4885,  757.4374, 739.4340, 595.3797, 577.3832, 433.3342, 415.3246 | **√** |  |
|  |  | [M-H]^-^ | 1211.5697 | 1211.5679 | -1.5 |  |  | 1079.5256, 1049.5209, 917.4716,  755.4220, 593.3689 |  |  |
| 42 | 12.55 | [M+NH_4_]^+^ | 1232.6275 | 1232.6268 | -0.6 | C_56_H_94_O_28_ | Timosaponin I_1_ | 1215.6027, 1197.5989, 1065.5284,  903.4888, 741.4311, 579.3940, 417.3344 | **√** |  |
|  |  | [M-H]^-^ | 1213.5853 | 1213.5796 | -4.7 |  |  | 1081.5393, 1051.5350, 919.4942, 757.4374 |  |  |
| 43^*^ | 13.68 | [M+NH_4_]^+^ | 936.5168 | 936.5191 | 2.5 | C_45_H_74_O_19_ | Timosaponin D | 919.4918, 757.4353, 595.3840, 433.3360, 415.3218, 289.2216, 271.2054 | **√** | **√** |
|  |  | [M-H]^-^ | 917.4746 | 917.4776 | 3.3 |  |  | 755.4217, 593.3717 |  |  |
| 44 | 14.65 | [M+NH_4_]^+^ | 936.5168 | 936.5172 | 0.4 | C_45_H_74_O_19_ | Timosaponin R | 919.4978, 757.4329, 595.3845, 433.3324, 415.3241, 289.2258, 271.2093 | **√** | **√** |
|  |  | [M-H]^-^ | 917.4746 | 917.4701 | -4.9 |  |  | 755.4230, 593.3651 |  |  |
| 45 | 15.34 | [M+NH_4_]^+^ | 936.5168 | 936.5191 | 2.5 | C_45_H_74_O_19_ | Timosaponin P | 919.4902, 757.4334, 595.3846, 433.3352, 415.3172, 289.2210, 271.2048 |  |  |
|  |  | [M-H]^-^ | 917.4746 | 917.4750 | 0.4 |  |  | 755.4209, 593.3732 |  |  |
| 46 | 15.78 | [M+NH_4_]^+^ | 950.5325 | 950.5332 | 0.7 | C_46_H_76_O_19_ | Timosaponin Q | 771.4515, 609.4142, 447.3258 |  |  |
|  |  | [M-H]^-^ | 931.4903 | 931.4858 | -4.8 |  |  | 769.4335, 607.3878, 445.3358 |  |  |
| 47 | 15.89 | [M+NH_4_]^+^ | 1258.6432 | 1258.6439 | 0.6 | C_58_H_96_O_28_ | Dehydration product of Timosaponin BⅥ | 1079.5664, 917.4997, 755.4593, 737.4368, 593.3944, 575.3853, 413.3002 |  |  |
|  |  | [M-H]^-^ | 1239.6010 | 1239.6024 | 1.1 |  |  | 1077.5539, 933.4958, 915.4839, 771.4578, 753.4441 |  |  |
| 48 | 16.23 | [M+NH_4_]^+^ | 1230.6119 | 1230.6152 | 2.7 | C_56_H_92_O_28_ | Timosaponin H_1_ | 1213.5946, 1195.5748, 901.4831,  757.4374, 739.4269, 595.3915, 577.3820, 433.3240, 415.3279 | **√** |  |
|  |  | [M-H]^-^ | 1211.5697 | 1211.5636 | -5.0 |  |  | 1079.5244, 1049.5139, 917.4774,  755.4234, 593.3666 |  |  |
| 49 | 16.37 | [M+H]+ | 1211.5697 | 1211.5736 | 3.2 | C_56_H_90_O_28_ | 25(27)-ene-Timosaponin H_1_ or its isomer | 1193.5610, 899.4615, 755.4324, 737.4166, 593.3769, 575.3660, 431.3225, 413.2920 |  |  |
|  |  | [M-H]^-^ | 1209.5540 | 1209.5565 | 2.1 |  |  | 1077.5287, 1047.5034, 915.4565, 753.4052 |  |  |
| 50 | 16.82 | [M+NH_4_]^+^ | 1230.6119 | 1230.6152 | 2.7 | C_56_H_92_O_28_ | 25*R*-timosaponin H_1_ | 1213.5946, 1195.5759, 901.4861,  757.4509, 739.4342, 595.3915, 577.3832, 433.3342, 415.3207 | **√** |  |
|  |  | [M-H]^-^ | 1211.5697 | 1211.5692 | -0.4 |  |  | 1079.5281, 1049.5179, 917.4786,  755.4200, 593.3709 |  |  |
| 51 | 17.30 | [M+NH_4_]^+^ | 1082.5747 | 1082.5706 | -3.8 | C_51_H_84_O_23_ | Isomer of timosaponin BⅣ | 1065.5506, 903.4948, 741.4426, 579.3915, 417.3326, 399.3199, 273.2318, 255.2088 | **√** | **√** |
|  |  | [M-H]^-^ | 1063.5325 | 1063.5303 | -2.1 |  |  | 901.4787, 739.4277, 577.3834 |  |  |
| 52 | 17.64 | [M+NH_4_]^+^ | 1082.5747 | 1082.5728 | -1.8 | C_51_H_84_O_23_ | Isomer of timosaponin BⅣ | 1065.5468, 903.5022, 741.4412, 579.4033, 417.3346, 399.3232, 273.2152, 255.1743 | **√** |  |
|  |  | [M-H]^-^ | 1063.5325 | 1063.5280 | -4.2 |  |  | 901.4811, 739.4244, 577.3695 |  |  |
| 53 | 18.33 | [M+H]+ | 901.4797 | 901.4774 | -2.6 | C_45_H_72_O_18_ | 25(27)-ene-timosaponin BⅢ or its isomer | 739.4289, 577.3742, 415.3189, 397.3075 | **√** | **√** |
|  |  | [M-H]^-^ | 899.4640 | 899.4652 | 1.3 |  |  | 737.4117, 575.3533 |  |  |
| 54 | 20.25 | [M+H]+ | 903.4953 | 903.4975 | 2.4 | C_45_H_74_O_18_ | Timosaponin BⅢ | 741.4461, 579.3901, 417.3351, 399.3241, 273.2246, 255.2186 | **√** | **√** |
|  |  | [M-H]^-^ | 901.4797 | 901.4814 | 1.9 |  |  | 739.4265, 577.3761 |  |  |
| 55 | 20.67 | [M+H]+ | 903.4953 | 903.4975 | 2.4 | C_45_H_74_O_18_ | 25*R*-timosaponin BⅢ | 741.4395, 579.3927, 417.3379, 399.3245, 273.2182, 255.2125 | **√** | **√** |
|  |  | [M-H]^-^ | 901.4797 | 901.4810 | 1.4 |  |  | 739.4301, 577.3704 |  |  |
| 56 | 21.31 | [M+H]+ | 903.4953 | 903.4941 | -1.3 | C_45_H_74_O_18_ | Timosaponin C | 741.4388, 579.3871, 417.3377, 399.3251, 273.2158, 255.2146 | **√** | **√** |
|  |  | [M-H]^-^ | 901.4797 | 901.4792 | -0.6 |  |  | 739.4310, 577.3771 |  |  |
| 57 | 21.63 | [M+H]+ | 903.4953 | 903.4922 | -3.4 | C_45_H_74_O_18_ | Macrostemonoside F | 741.4403, 579.3863, 417.3378, 399.3245, 273.2219, 255.2151 | **√** | **√** |
|  |  | [M-H]^-^ | 901.4797 | 901.4822 | 2.8 |  |  | 739.4257, 577.3735 |  |  |
| 58 | 22.37 | [M+NH_4_]^+^ | 1082.5747 | 1082.5771 | 2.2 | C_51_H_84_O_23_ | Timosaponin BⅣ | 1065.5442, 903.4987, 741.4489, 579.3860, 417.3368, 399.3273, 273.2199, 255.2072 | **√** | **√** |
|  |  | [M-H]^-^ | 1063.5325 | 1063.5348 | 2.2 |  |  | 901.4796, 739.4300, 577.3790 |  |  |
| 59 | 22.72 | [M+NH_4_]^+^ | 1080.5591 | 1080.5637 | 4.3 | C_51_H_82_O_23_ | 25(27)-ene-Timosaponin BⅣ or its isomer | 901.4836, 739.4288, 577.3740, 415.3206 |  |  |
|  |  | [M-H]^-^ | 1061.5169 | 1061.5217 | 4.5 |  |  | 899.4651, 737.4127 |  |  |
| 60 | 23.03 | [M+NH_4_]^+^ | 1212.6013 | 1212.5977 | -3.0 | C_56_H_90_O_27_ | 25(27)-ene-Timosaponin BⅣ+Xyl | 1033.5244, 739.4270, 577.3697, 415.3248 | **√** | **√** |
|  |  | [M-H]^-^ | 1193.5591 | 1193.5636 | 3.8 |  |  | 1061.5181, 899.4679, 737.4117 |  |  |
| 61 | 23.14 | [M+NH_4_]^+^ | 1214.6170 | 1214.6140 | -2.5 | C_56_H_92_O_27_ | Timosaponin BⅣ+Xyl | 1035.5364, 741.4472, 579.3898, 417.3331 |  |  |
|  |  | [M-H]^-^ | 1195.5748 | 1195.5789 | 3.4 |  |  | 1063.5351, 901.4813, 739.4225 |  |  |
| 62 | 23.28 | [M+H]+ | 903.4953 | 903.4922 | -3.4 | C_45_H_74_O_18_ | Xilingsaponin B | 741.4421, 579.3908, 417.3380, 399.3252, 273.2230, 255.2101 |  |  |
|  |  | [M-H]^-^ | 901.4797 | 901.4784 | -1.4 |  |  | 739.4289, 577.3750 |  |  |
| 63 | 23.42 | [M+NH_4_]^+^ | 1214.6170 | 1214.6115 | -4.5 | C_56_H_92_O_27_ | 25*R*-timosaponin BⅣ+Xyl | 1035.5380, 741.4435, 579.3877, 417.3345 | **√** |  |
|  |  | [M-H]^-^ | 1195.5748 | 1195.5774 | 2.2 |  |  | 1063.5320, 901.4813, 739.4247 |  |  |
| 64^*^ | 23.52 | [M+H]+ | 741.4425 | 741.4437 | 1.6 | [C](https://pubchem.ncbi.nlm.nih.gov/#query=C39H64O13)_[39](https://pubchem.ncbi.nlm.nih.gov/#query=C39H64O13)_[H](https://pubchem.ncbi.nlm.nih.gov/#query=C39H64O13)_[64](https://pubchem.ncbi.nlm.nih.gov/#query=C39H64O13)_[O](https://pubchem.ncbi.nlm.nih.gov/#query=C39H64O13)_[13](https://pubchem.ncbi.nlm.nih.gov/#query=C39H64O13)_ | Timosaponin AⅣ | 741.4456, 579.3892, 417.3355, 399.3257, 273.2219, 255.2136 | **√** | **√** |
|  |  | [M-H]^-^ | 739.4269 | 739.4290 | 2.8 |  |  | 577.3690, 457.3341 |  |  |
| 65 | 23.85 | [M+H]+ | 917.4746 | 917.4703 | -4.7 | [C](https://pubchem.ncbi.nlm.nih.gov/#query=C39H64O13)_[45](https://pubchem.ncbi.nlm.nih.gov/#query=C39H64O13)_[H](https://pubchem.ncbi.nlm.nih.gov/#query=C39H64O13)_[72](https://pubchem.ncbi.nlm.nih.gov/#query=C39H64O13)_[O](https://pubchem.ncbi.nlm.nih.gov/#query=C39H64O13)_[19](https://pubchem.ncbi.nlm.nih.gov/#query=C39H64O13)_ | 25(27)-ene-Timosaponin D or its isomer | 755.4209, 593.3994, 431.3118, 413.3010, 269.1905 |  |  |
|  |  | [M-H]^-^ | 915.4590 | 915.4583 | -0.8 |  |  | 753.4083, 591.3899 |  |  |
| 66 | 23.93 | [M+H]+ | 873.4848 | 873.4813 | -4.0 | C_44_H_72_O_17_ | Timosaponin X-OCH_3_ | 711.4297, 579.3890, 417.3362, 399.3271, 273.2232, 255.2114 |  |  |
|  |  | [M-H]^-^ | 871.4691 | 871.4694 | 0.3 |  |  | 709.4177, 577.3757 |  |  |
| 67 | 24.12 | [M+NH_4_]^+^ | 774.4640 | 774.4657 | 2.2 | C_39_H_64_O_14_ | Timosaponin G(C39) | 595.3836, 433.3321, 415.3241, 397.3109,  289.2240, 271.2073, 253.2047 |  |  |
|  |  | [M-H]^-^ | 755.4218 | 755.4233 | 2.0 |  |  | 593.3719, 431.3189 |  |  |
| 68^*^ | 24.42 | [M+H-H_2_O]^+^ | 741.4425 | 741.4458 | 4.5 | C_39_H_66_O_14_ | AnemarrhenasaponinⅠ | 776.4819, 579.3902, 417.3352, 399.3274, 381.3169, 271.2025, 253.1948 | **√** | **√** |
|  |  | [M-H]^-^ | 757.4374 | 757.4391 | 2.2 |  |  | 595.3831, 433.3329 |  |  |
| 69 | 24.75 | [M+H-H_2_O]^+^ | 741.4425 | 741.4388 | -5.0 | C_39_H_66_O_14_ | AnemarrhenasaponinⅡ | 776.4786, 579.3904, 417.3366, 399.3252, 381.3164, 271.2125, 253.2008 | **√** | **√** |
|  |  | [M-H]^-^ | 757.4374 | 757.4382 | 1.1 |  |  | 595.3812, 433.3320 |  |  |
| 70 | 24.84 | [M+NH_4_]^+^ | 774.4640 | 774.4657 | 2.2 | C_39_H_64_O_14_ | Anemarrhenasaponin Ⅲ | 595.3820, 433.3347, 415.3266, 397.3130, 289.2192, 271.2010, 253.2012 | **√** | **√** |
|  |  | [M-H]^-^ | 755.4218 | 755.4208 | -1.3 |  |  | 593.3715, 431.3154 |  |  |
| 71 | 25.26 | [M+H-H_2_O]^+^ | 755.4218 | 755.4212 | -0.8 | C_39_H_64_O_15_ | Timosaponin F(C39) | 611.3810, 449.3237, 431.3243 |  |  |
|  |  | [M-H]^-^ | 771.4167 | 771.4182 | 1.9 |  |  | 609.3677, 447.3249 |  |  |
| 72 | 25.75 | [M+H-H_2_O]^+^ | 725.4476 | 725.4473 | -0.4 | C_39_H_66_O_13_ | Timosaponin X-Glc | 579.3884, 563.3690, 431.3253, 417.3387 |  |  |
|  |  | [M-H]^-^ | 741.4425 | 741.4459 | 4.6 |  |  | 565.3757, 433.3374 |  |  |
| 73 | 25.96 | [M+NH_4_]^+^ | 774.4640 | 774.4621 | -2.5 | C_39_H_64_O_14_ | Timosaponin AⅡ | 595.3849, 433.3315, 415.3216, 397.3116, 289.2168, 271.2163, 253.2036 | **√** | **√** |
|  |  | [M-H]^-^ | 755.4218 | 755.4240 | 2.9 |  |  | 593.3722, 431.3152 |  |  |
| 74 | 26.55 | [M+H]+ | 579.3897 | 579.3884 | -2.2 | C_33_H_54_O_8_ | Timosaponin AⅠ | 417.3377, 273.2224, 255.2147 | **√** | **√** |
|  |  | [M+HCOO]^-^ | 623.3795 | 623.3817 | 3.5 |  |  | 577.3717 |  |  |
| 75^*^ | 26.90 | [M+NH_4_]^+^ | 758.4691 | 758.4723 | 4.2 | [C](https://pubchem.ncbi.nlm.nih.gov/#query=C39H64O13)_[39](https://pubchem.ncbi.nlm.nih.gov/#query=C39H64O13)_[H](https://pubchem.ncbi.nlm.nih.gov/#query=C39H64O13)_[64](https://pubchem.ncbi.nlm.nih.gov/#query=C39H64O13)_[O](https://pubchem.ncbi.nlm.nih.gov/#query=C39H64O13)_[13](https://pubchem.ncbi.nlm.nih.gov/#query=C39H64O13)_ | Timosaponin AⅢ | 741.4466, 579.3861, 417.3384, 399.3248, 273.2190, 255.2058 | **√** | **√** |
|  |  | [M-H]^-^ | 739.4269 | 739.4297 | 3.8 |  |  | 577.3774, 457.3322 |  |  |
| 76 | 27.47 | [M+NH_4_]^+^ | 758.4691 | 758.4683 | -1.1 | [C](https://pubchem.ncbi.nlm.nih.gov/#query=C39H64O13)_[39](https://pubchem.ncbi.nlm.nih.gov/#query=C39H64O13)_[H](https://pubchem.ncbi.nlm.nih.gov/#query=C39H64O13)_[64](https://pubchem.ncbi.nlm.nih.gov/#query=C39H64O13)_[O](https://pubchem.ncbi.nlm.nih.gov/#query=C39H64O13)_[13](https://pubchem.ncbi.nlm.nih.gov/#query=C39H64O13)_ | 25*R*-timosaponin AⅢ | 741.4468, 579.3915, 417.3332, 399.3237, 273.2237, 255.2138 |  | **√** |
|  |  | [M-H]^-^ | 739.4269 | 739.4290 | 2.8 |  |  | 577.3707, 457.3345 |  |  |
| 77 | 27.66 | [M+NH_4_]^+^ | 904.5270 | 904.5303 | 3.6 | [C](https://pubchem.ncbi.nlm.nih.gov/#query=C39H64O13)_[45](https://pubchem.ncbi.nlm.nih.gov/#query=C39H64O13)_[H](https://pubchem.ncbi.nlm.nih.gov/#query=C39H64O13)_[74](https://pubchem.ncbi.nlm.nih.gov/#query=C39H64O13)_[O](https://pubchem.ncbi.nlm.nih.gov/#query=C39H64O13)_[1](https://pubchem.ncbi.nlm.nih.gov/#query=C39H64O13)7_ | Timosaponin V | 887.5014, 725.4437, 563.4027, 401.3369 |  |  |
|  |  | [M-H]^-^ | 885.4848 | 885.4804 | -5.0 |  |  | 723.4337, 561.3911, 399.3157 |  |  |

Note, “**√**” expressed as the prototype constituents from AR were detected in rat serum or urine; “*”expressed as the constituents were identified by the reference standards. The reference standards of neomangiferin, timosaponin BⅡ, timosaponin D, anemarrhenasaponin Ⅰ, timosaponin AⅣ, and timosaponin AⅢ were obtained from the National Institute for the Control of Pharmaceutical and Biological Products (Beijing, China).

1.6 Supplementary **Table S6. Identification of AR metabolites in rat serum and urine using UHPLC-Q-TOF-MS**

| **No** | **t_R_/min** | **Ion mode** | **Mass(*m/z*)** | | **Error**  **(ppm)** | **Molecular**  **formula** | **Identification** | **MS/MS fragment ions (*m/z*)** | **Observed** | |
| --- | --- | --- | --- | --- | --- | --- | --- | --- | --- | --- |
|  |  |  | **Calculated** | **Measured** |  |  |  |  | **Serum** | **Urine** |
| M1 | 2.22 | [M+H]^+^ | 613.1405 | 613.1413 | 1.3 | C_26_H_28_O_17_ | Monomethylation metabolites of mangiferin glucuronidation | / | **√** |  |
|  |  | [M-H]^-^ | 611.1248 | 611.1267 | 3.1 |  |  | 435.0931, 421.0797 |  |  |
| M2 | 2.56 | [M-H]^-^ | 515.0496 | 515.0511 | 2.9 | C_20_H_20_O_14_S | Monomethylation metabolites of mangiferin sulfation | 435.0941, 421.0791, 259.0280, 215.0362, 187.0396, 107.0116 |  | **√** |
| M3 | 2.73 | [M-H]^-^ | 435.0927 | 435.0918 | -2.1 | C_20_H_20_O_11_ | Monomethylation metabolites of mangiferin | 421.0743, 259.0255, 187.0408, 107.0137 | **√** |  |
| M4 | 2.82 | [M-H]^-^ | 515.0496 | 515.0501 | 1.0 | C_20_H_20_O_14_S | Monomethylation metabolites of mangiferin sulfation | 435.0931, 421.0744, 343.0431, 331.0461, 301.0338, 259.0248, 203.0301, 187.0427, 107.0131 | **√** |  |
| M5 | 3.48 | [M-H]^-^ | 501.0339 | 501.0363 | 4.8 | C_19_H_18_O_14_S | Mangiferin sulfation | 421.0757 |  | **√** |
| M6 | 3.57 | [M-H]^-^ | 435.0564 | 435.0571 | 1.6 | C_19_H_16_O_12_ | Norathyriol glucuronidation | 259.02569, 231.0306, 187.0381, 107.0143 |  | **√** |
| M7 | 3.60 | [M+H]^+^ | 437.1084 | 437.1082 | -0.5 | C_20_H_20_O_11_ | Monomethylation metabolites of mangiferin | / | **√** |  |
|  |  | [M-H]^-^ | 435.0927 | 435.0941 | 3.2 |  |  | 421.0765 |  |  |
| M8 | 15.42 | [M-H]^-^ | 449.1081 | 449.1084 | -0.7 | C_21_H_22_O_11_ | Dimethylation metabolites of mangiferin | 435.0965, 421.0778 | **√** |  |
| M9 | 25.25 | [M+H]^+^ | 449.3267 | 449.3250 | -3.8 | C_27_H_44_O_5_ | Digitogenin | 433.3318, 415.3185, 373.2730, 355.2655, 303.1770, 263.1714 |  | **√** |
| M10 | 25.43 | [M+H]^+^ | 433.3318 | 433.3309 | -2.1 | C_27_H_44_O_4_ | Markogenin or neogitogenin | 415.3204, 255.1627, 237.1794 |  | **√** |
| M11 | 27.69 | [M+H]^+^ | 417.3369 | 417.3378 | 2.2 | C_27_H_44_O_3_ | Sarsasapogenin | 399.3286, 381.3222, 318.2999, 274.2610 |  | **√** |

Note, “**√**” expressed as the prototype constituents from AR were detected in rat serum or urine.

**1.7 Supplementary Table S7. The molecular formulas, representative components, and canonical SMILES of the selected prototype constituents and metabolites were prepared for network pharmacology**

| **No.** | **Molecular formula** | **Representative prototypes and**  **metabolites** | **SMILES** |
| --- | --- | --- | --- |
| AR1 | C_25_H_28_O_16_ | Neomangiferin | C1=C2C(=CC(=C1OC3C(C(C(C(O3)CO)O)O)O)O)OC4=C(C2=O)C(=C(C(=C4)O)C5C(C(C(C(O5)CO)O)O)O)O |
| AR2 | C_19_H_18_O_11_ | Mangiferin | C1=C2C(=CC(=C1O)O)OC3=C(C2=O)C(=C(C(=C3)O)C4C(C(C(C(O4)CO)O)O)O)O |
| AR3 | C_33_H_54_O_8_ | Timosaponin AⅠ | CC1CCC2(C(C3C(O2)CC4C3(CCC5C4CCC6C5(CCC(C6)OC7C(C(C(C(O7)CO)O)O)O)C)C)C)OC1 |
| AR4 | C_39_H_64_O_13_ | Timosaponin AⅢ/  Timosaponin AⅣ | CC1CCC2(C(C3C(O2)CC4C3(CCC5C4CCC6C5(CCC(C6)OC7C(C(C(C(O7)CO)O)O)OC8C(C(C(C(O8)CO)O)O)O)C)C)C)OC1 |
| AR5 | C_39_H_64_O_14_ | Timosaponin AⅡ/  Anemarrhenasaponin Ⅲ | CC1CCC2(C(C3C(O2)C(C4C3(CCC5C4CCC6C5(CCC(C6)OC7C(C(C(C(O7)CO)O)O)OC8C(C(C(C(O8)CO)O)O)O)C)C)O)C)OC1 |
| AR6 | C_39_H_66_O_14_ | Anemarrhenasaponin Ⅰ/  Anemarrhenasaponin Ⅱ | CC1C2C(C(C3C2(CCC4C3CCC5C4(CCC(C5)OC6C(C(C(C(O6)CO)O)O)OC7C(C(C(C(O7)CO)O)O)O)C)C)O)OC1(CCC(C)C)O |
| AR7 | C_51_H_84_O_23_ | Timosaponin BⅣ | C[C@H](CCC1=C(C)C2C(CC3C4CCC5CC(CC[C@]5(C)C4CC[C@]23C)OC2OC(CO)C(O)C(O)C2OC2OC(CO)C(OC3OC(CO)C(O)C(O)C3O)C(O)C2O)O1)COC1OC(CO)C(O)C(O)C1O |
| AR8 | C_45_H_74_O_18_ | Timosaponin BⅢ/  Timosaponin C/  Macrostemonoside F | CC1=C(OC2C1C3(CCC4C(C3C2)CCC5C4(CCC(C5)OC6C(C(C(C(O6)CO)O)O)OC7C(C(C(C(O7)CO)O)O)O)C)C)CCC(C)COC8C(C(C(C(O8)CO)O)O)O |
| AR9 | C_45_H_74_O_19_ | Timosaponin D/  Timosaponin M/  Timosaponin L/  Timosaponin R | CC1=C(OC2C1C3(CCC4C(C3C2)CCC5C4(CC(C(C5)OC6C(C(C(C(O6)CO)O)O)OC7C(C(C(C(O7)CO)O)O)O)O)C)C)CCC(C)COC8C(C(C(C(O8)CO)O)O)O |
| AR10 | C_45_H_76_O_19_ | Timosaponin BⅡ/  Officinalisinin-Ⅰ | CC1C2C(CC3C2(CCC4C3CCC5C4(CCC(C5)OC6C(C(C(C(O6)CO)O)O)OC7C(C(C(C(O7)CO)O)O)O)C)C)OC1(CCC(C)COC8C(C(C(C(O8)CO)O)O)O)O |
| AR11 | C_45_H_76_O_20_ | Timosaponin E_1_ | C[C@@H](CCC1(O)OC2[C@H](O)C3C4CCC5C[C@H](CC[C@]5(C)C4CC[C@]3(C)C2[C@@H]1C)O[C@@H]1O[C@H](CO)[C@H](O)[C@H](O)[C@H]1O[C@@H]1O[C@H](CO)[C@@H](O)[C@H](O)[C@H]1O)CO[C@@H]1O[C@H](CO)[C@@H](O)[C@H](O)[C@H]1O |
| AR12 | C_45_H_76_O_20_ | Macrostemonoside J  /Timosaponin N | CC1C2C(CC3C2(CCC4C3CCC5C4(CC(C(C5)OC6C(C(C(C(O6)CO)O)O)OC7C(C(C(C(O7)CO)O)O)O)O)C)C)OC1(CCC(C)COC8C(C(C(C(O8)CO)O)O)O)O |
| AR13 | C_51_H_86_O_24_ | Asparagoside G/  Tomatoside A | CC1C2C(CC3C2(CCC4C3CCC5C4(CCC(C5)OC6C(C(C(C(O6)CO)OC7C(C(C(C(O7)CO)O)O)O)OC8C(C(C(C(O8)CO)O)O)O)O)C)C)OC1(CCC(C)COC9C(C(C(C(O9)CO)O)O)O)O |
| AR14 | C_56_H_92_O_28_ | Timosaponin H_1_/  Timosaponin C_1_ | C[C@H]1[C@H]2[C@H](C[C@@H]3[C@@]2(CC[C@H]4[C@H]3CC=C5[C@@]4(CC[C@@H](C5)O[C@H]6[C@@H]([C@H]([C@H]([C@H](O6)CO)O[C@H]7[C@@H]([C@H]([C@@H]([C@H](O7)CO)O)O[C@H]8[C@@H]([C@H]([C@@H](CO8)O)O)O)O[C@H]9[C@@H]([C@H]([C@@H]([C@H](O9)CO)O)O)O)O)O)C)C)OC1(CC[C@H](C)CO[C@H]1[C@@H]([C@H]([C@@H]([C@H](O1)CO)O)O)O)O |
| AR15 | C_56_H_92_O_29_ | Karatavioside C | CC1C2C(CC3C2(CCC4C3CC=C5C4(CC(C(C5)OC6C(C(C(C(O6)CO)OC7C(C(C(C(O7)CO)O)OC8C(C(C(CO8)O)O)O)OC9C(C(C(C(O9)CO)O)O)O)O)O)O)C)C)OC1(CCC(C)COC1C(C(C(C(O1)CO)O)O)O)O |
| AR16 | C_56_H_94_O_28_ | Timosaponin D_1_ /Timosaponin I_1_ | CC(CCC1(O)OC2CC3C4CCC5CC(CCC5(C)C4CCC3(C)C2C1C)OC1OC(CO)C(OC2OC(CO)C(O)C(OC3OCC(O)C(O)C3O)C2OC2OC(CO)C(O)C(O)C2O)C(O)C1O)COC1OC(CO)C(O)C(O)C1O |
| AR17 | C_56_H_94_O_29_ | Purpureagitosid | CC1C2C(CC3C2(CCC4C3CCC5C4(CC(C(C5)OC6C(C(C(C(O6)CO)OC7C(C(C(C(O7)CO)O)OC8C(C(C(CO8)O)O)O)OC9C(C(C(C(O9)CO)O)O)O)O)O)O)C)C)OC1(CCC(C)COC1C(C(C(C(O1)CO)O)O)O)O |
| AR18 | C_58_H_98_O_29_ | Timosaponin BⅥ | COC1(CCC(C)COC2OC(CO)C(O)C(O)C2O)OC2CC3C4CCC5CC(CCC5(C)C4CCC3(C)C2C1C)OC1OC(CO)C(OC2OC(CO)C(O)C(OC3OC(CO)C(O)C(O)C3O)C2OC2OC(CO)C(O)C(O)C2O)C(O)C1O |
| MA1 | C_13_H_8_O_6_ | Norathyriol | C1=C(C=C2C(=C1O)C(=O)C3=CC(=C(C=C3O2)O)O)O |
| MA2 | C_27_H_44_O_5_ | Digitogenin | CC1CCC2(C(C3C(O2)C(C4C3(CCC5C4CCC6C5(CC(C(C6)O)O)C)C)O)C)OC1 |
| MA3 | C_27_H_44_O_4_ | Markogenin /Neogitogenin | CC1CCC2(C(C3C(O2)CC4C3(CCC5C4CCC6C5(CC(C(C6)O)O)C)C)C)OC1 |
| MA4 | C_27_H_44_O_3_ | Sarsasapogenin | CC1CCC2(C(C3C(O2)CC4C3(CCC5C4CCC6C5(CCC(C6)O)C)C)C)OC1 |

**1.8 Supplementary Table S8. The characteristic parameters of top 20 targets of PPI network nodes**

| **Targets** | **Degree** | **AverageShortestPathLength** | **BetweennessCentrality** | **ClosenessCentrality** | **ClusteringCoefficient** |
| --- | --- | --- | --- | --- | --- |
| TP53 | 46 | 2.24186047 | 0.16733353 | 0.44605809 | 0.17101449 |
| AKT1 | 37 | 2.3255814 | 0.07530564 | 0.43 | 0.21171171 |
| STAT3 | 34 | 2.32093023 | 0.07555548 | 0.43086172 | 0.21212121 |
| TNF | 34 | 2.40465116 | 0.06065595 | 0.41586074 | 0.26381462 |
| SRC | 32 | 2.48837209 | 0.05448363 | 0.40186916 | 0.2358871 |
| HSP90AA1 | 31 | 2.49302326 | 0.06083875 | 0.4011194 | 0.15268817 |
| IL6 | 31 | 2.41395349 | 0.08037348 | 0.41425819 | 0.28602151 |
| CTNNB1 | 26 | 2.48837209 | 0.04148717 | 0.40186916 | 0.23384615 |
| NFKB1 | 26 | 2.46511628 | 0.03975421 | 0.40566038 | 0.30769231 |
| ESR1 | 25 | 2.3627907 | 0.08478421 | 0.42322835 | 0.32333333 |
| RELA | 25 | 2.49302326 | 0.02728916 | 0.4011194 | 0.29666667 |
| MAPK1 | 24 | 2.44651163 | 0.02951342 | 0.40874525 | 0.30434783 |
| BCL2 | 24 | 2.57209302 | 0.03878856 | 0.38878843 | 0.28623188 |
| HRAS | 23 | 2.70232558 | 0.03308246 | 0.37005164 | 0.27667984 |
| MAPK3 | 23 | 2.47906977 | 0.02778318 | 0.40337711 | 0.27667984 |
| IL1B | 23 | 2.66511628 | 0.03926037 | 0.37521815 | 0.41897233 |
| EGFR | 23 | 2.49302326 | 0.02876302 | 0.4011194 | 0.29644269 |
| PIK3CA | 21 | 2.68372093 | 0.01489775 | 0.37261698 | 0.35238095 |
| IFNG | 21 | 2.8 | 0.02601949 | 0.35714286 | 0.41904762 |
| CASP3 | 20 | 2.60465116 | 0.03658919 | 0.38392857 | 0.27894737 |

**1.9 Supplementary Table S9. Enrichment analysis of target pathway of AR in treatment of T2DM**

| **Term** | **Pathway** | **PValue** | **Count** | **Genes** |
| --- | --- | --- | --- | --- |
| hsa04933 | AGE-RAGE signaling pathway in diabetic complications | 4.72E-37 | 42 | CXCL8, SERPINE1, PIK3CD, PIK3CB, TNF, AGER, RELA, ICAM1, MAPK9, MAPK8, CCND1, CASP3, CCL2, AKT1, MAPK1, RAC1, HRAS, MAPK3, TGFB1, VCAM1, SMAD3, NOS3, STAT3, FN1, PRKCA, MAPK14, SELE, TGFBR1, NFKB1, AGT, VEGFA, MAPK10, COL1A1, IL1A, IL6, PIK3CA, COL4A1, CDK4, IL1B, BCL2, BAX, NOX4 |
| hsa05417 | Lipid and atherosclerosis | 4.57E-36 | 55 | GSK3B, CXCL8, PIK3CD, PIK3CB, TNF, ICAM1, CASP9, IKBKB, CASP7, TBK1, CASP8, CASP3, IL12B, AKT1, IL12A, CD36, RAC1, HRAS, HSP90AA1, CHUK, IL18, PRKCA, MMP9, PIK3CA, IL1B, PPARG, TLR6, TP53, SRC, AGER, RELA, MAPK9, MAPK8, CCL5, CCL2, MAPK1, NLRP3, MAPK3, VCAM1, IFNB1, NOS3, STAT3, MAPK14, SELE, NFKB1, NFKBIA, MAPK10, CYP2C9, IL6, BCL2, BAX, FAS, NFE2L2, BCL2L1, HSPA1A |
| hsa04668 | TNF signaling pathway | 6.90E-25 | 35 | CSF2, XIAP, PIK3CD, PIK3CB, PTGS2, TNF, RELA, ICAM1, IKBKB, MAPK9, CASP7, MAPK8, CASP8, CASP3, CCL5, CCL2, AKT1, MAPK1, DNM1L, MAPK3, VCAM1, CHUK, IFNB1, MAPK14, SELE, MMP9, NFKB1, MAPK10, NFKBIA, IL6, CREB1, PIK3CA, IRF1, IL1B, FAS |
| hsa04151 | PI3K-Akt signaling pathway | 2.56E-23 | 54 | GSK3B, CSF3, FLT3, FLT4, PIK3CD, PIK3CB, FGF2, PIK3CG, IGF1R, CASP9, IKBKB, CCND2, CCND1, KDR, AKT1, RAC1, HRAS, PDGFRB, HSP90AA1, CHUK, PRKCA, NGF, PGF, CREB1, PIK3CA, COL4A1, KIT, RAF1, MET, TP53, PRKAA1, EGFR, RELA, INS, PPP2CA, MAPK1, MAPK3, IFNB1, NOS3, FN1, MTOR, NFKB1, IL2, VEGFA, COL1A1, IL6, CDK6, CDK4, IL2RB, CDK2, BCL2, MDM2, TEK, BCL2L1 |
| hsa04917 | Prolactin signaling pathway | 2.26E-22 | 27 | GSK3B, SRC, PIK3CD, TNFRSF11A, PIK3CB, RELA, INS, CYP17A1, MAPK9, MAPK8, CCND2, CCND1, AKT1, TNFSF11, MAPK1, HRAS, MAPK3, STAT3, MAPK14, ESR1, GCK, ESR2, NFKB1, MAPK10, PIK3CA, IRF1, RAF1 |
| hsa04620 | Toll-like receptor signaling pathway | 2.16E-20 | 30 | CXCL8, PIK3CD, PIK3CB, TNF, RELA, IKBKB, MAPK9, MAPK8, CASP8, TBK1, CCL5, IL12B, AKT1, MAPK1, IL12A, RAC1, MAPK3, CHUK, IFNB1, MAPK14, NFKB1, MAPK10, NFKBIA, IL6, PIK3CA, IL1B, TLR9, TLR8, TLR7, TLR6 |
| hsa04931 | Insulin resistance | 2.16E-20 | 30 | GSK3B, PRKAA1, PIK3CD, PYGM, PIK3CB, SLC2A4, TNF, RELA, INS, IKBKB, MAPK9, RPS6KA3, MAPK8, AKT1, CD36, PPARGC1A, SREBF1, PTPN1, CPT1A, NOS3, STAT3, MTOR, NFKB1, AGT, MAPK10, NFKBIA, IL6, CREB1, PIK3CA, PPARA |
| hsa04068 | FoxO signaling pathway | 6.09E-19 | 31 | PRKAA1, PIK3CD, PIK3CB, SLC2A4, EGFR, IGF1R, INS, IKBKB, MAPK9, MAPK8, CCND2, CCND1, AKT1, MAPK1, HRAS, MAPK3, IL10, TGFB1, SMAD3, CHUK, STAT3, BRAF, MAPK14, TGFBR1, MAPK10, IL6, PIK3CA, CAT, CDK2, MDM2, RAF1 |
| hsa04657 | IL-17 signaling pathway | 1.35E-17 | 26 | GSK3B, CSF3, CSF2, CXCL8, PTGS2, TNF, RELA, IKBKB, MAPK9, MAPK8, CASP8, TBK1, CASP3, CCL2, MAPK1, MAPK3, HSP90AA1, CHUK, MAPK14, MMP9, NFKB1, MAPK10, NFKBIA, IL6, IFNG, IL1B |
| hsa04014 | Ras signaling pathway | 1.91E-16 | 37 | FLT3, FLT4, PIK3CD, PIK3CB, FGF2, RELA, EGFR, IGF1R, INS, IKBKB, MAPK9, GRIN2A, MAPK8, TBK1, KDR, ABL1, AKT1, MAPK1, RAC1, HRAS, MAPK3, PDGFRB, CHUK, PLA2G2A, PRKCA, NGF, GRIN2B, PGF, NFKB1, VEGFA, MAPK10, PIK3CA, KIT, TEK, RAF1, MET, BCL2L1 |
| hsa04010 | MAPK signaling pathway | 4.23E-16 | 41 | FLT3, FLT4, FGF2, TNF, RELA, EGFR, IGF1R, INS, IKBKB, MAPK9, RPS6KA3, MAPK8, CASP3, KDR, AKT1, MAPK1, RAC1, HRAS, MAPK3, PDGFRB, TGFB1, CHUK, PRKCA, BRAF, NGF, MAPK14, TGFBR1, PGF, NFKB1, VEGFA, MAPK10, IL1A, IL1B, KIT, FAS, PTPN7, TEK, RAF1, MET, TP53, HSPA1A |
| hsa04066 | HIF-1 signaling pathway | 4.66E-16 | 26 | SERPINE1, PIK3CD, PIK3CB, HIF1A, RELA, HK2, EGFR, IGF1R, HK1, INS, AKT1, HMOX1, MAPK1, MAPK3, NOS2, NOS3, STAT3, PRKCA, MTOR, NFKB1, VEGFA, IL6, IFNG, PIK3CA, BCL2, TEK |
| hsa04932 | Non-alcoholic fatty liver disease | 1.01E-15 | 30 | GSK3B, PRKAA1, CXCL8, PIK3CD, PIK3CB, TNF, RELA, INS, IKBKB, MAPK9, CASP7, MAPK8, CASP8, CASP3, AKT1, RAC1, SREBF1, TGFB1, MAPK14, NFKB1, MAPK10, IL1A, IL6, PIK3CA, IL1B, BAX, FAS, PPARG, CYP2E1, PPARA |
| hsa04015 | Rap1 signaling pathway | 1.82E-15 | 34 | SRC, FLT4, ITGB2, PIK3CD, PIK3CB, FGF2, EGFR, IGF1R, INS, GRIN2A, CDH1, CNR1, KDR, AKT1, MAPK1, RAC1, DRD2, HRAS, MAPK3, PDGFRB, PRKCA, BRAF, NGF, MAPK14, GRIN2B, PGF, VEGFA, PIK3CA, ADORA2B, KIT, CTNNB1, TEK, RAF1, MET |
| hsa04926 | Relaxin signaling pathway | 4.48E-15 | 27 | SRC, PIK3CD, PIK3CB, RELA, EGFR, MAPK9, MAPK8, AKT1, MAPK1, HRAS, MAPK3, TGFB1, NOS2, NOS3, PRKCA, MAPK14, MMP9, TGFBR1, NFKB1, VEGFA, MAPK10, NFKBIA, COL1A1, CREB1, PIK3CA, COL4A1, RAF1 |
| hsa04660 | T cell receptor signaling pathway | 8.58E-15 | 26 | GSK3B, CSF2, PIK3CD, PIK3CB, TNF, RELA, PPP2CA, IKBKB, MAPK9, MAPK8, AKT1, MAPK1, HRAS, MAPK3, IL10, CHUK, MAPK14, IL2, NFKB1, MAPK10, NFKBIA, PTPRC, IFNG, PIK3CA, CDK4, RAF1 |
| hsa04659 | Th17 cell differentiation | 4.43E-14 | 24 | HSP90AA1, TGFB1, SMAD3, CHUK, STAT3, AHR, MAPK14, HIF1A, TGFBR1, IL2, MTOR, RELA, NFKB1, MAPK10, NFKBIA, IKBKB, MAPK9, IL6, MAPK8, IFNG, IL1B, IL2RB, MAPK1, MAPK3 |
| hsa04910 | Insulin signaling pathway | 1.70E-13 | 26 | GSK3B, PRKAA1, PIK3CD, PYGM, PIK3CB, SLC2A4, HK2, ACACA, HK1, INS, IKBKB, MAPK9, MAPK8, AKT1, MAPK1, HRAS, PPARGC1A, MAPK3, SREBF1, PTPN1, BRAF, GCK, MTOR, MAPK10, PIK3CA, RAF1 |
| hsa05415 | Diabetic cardiomyopathy | 2.11E-13 | 31 | GSK3B, PIK3CD, PIK3CB, SLC2A4, AGER, RELA, INS, MAPK9, MAPK8, AKT1, TNNI3, CD36, RAC1, CTSD, G6PD, TGFB1, SMAD3, NOS3, GSR, PRKCA, MAPK14, MMP9, TGFBR1, MTOR, NFKB1, AGT, MAPK10, COL1A1, PIK3CA, REN, PPARA |
| hsa04071 | Sphingolipid signaling pathway | 6.91E-13 | 24 | NOS3, PIK3CD, PRKCA, PIK3CB, MAPK14, TNF, RELA, NFKB1, MAPK10, PPP2CA, MAPK9, MAPK8, PIK3CA, ADORA1, BCL2, BAX, AKT1, MAPK1, RAC1, RAF1, HRAS, CTSD, TP53, MAPK3 |
| hsa04930 | Type II diabetes mellitus | 2.31E-12 | 16 | PIK3CD, PIK3CB, SLC2A4, TNF, MTOR, GCK, HK2, INS, HK1, MAPK10, IKBKB, MAPK9, MAPK8, PIK3CA, MAPK1, MAPK3 |
