# Supplementary material for: Metabolomics integrated with network pharmacology and serum-urine pharmacochemistry unveils the antidiabetic mechanism of Anemarrhenae Rhizoma
Source: Front Endocrinol (Lausanne). 2025 Oct 9;16:1618584. doi: 10.3389/fendo.2025.1618584 (PMC12545018; doi:10.3389/fendo.2025.1618584)
Supplement: Supplementary file 2 [file DataSheet1.docx]

Supplementary Material

**1. Supplementary figure S1**


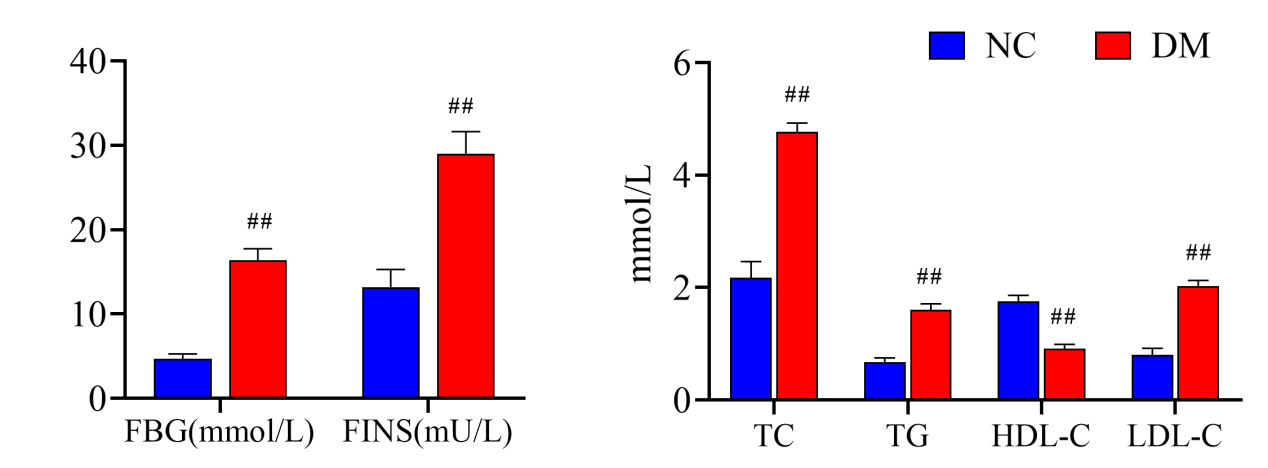


**Figure S1** Blood glucose and blood lipid levels in the normal control (NC) group and the diabetes model (DM) group

**2. Supplementary figure S2**


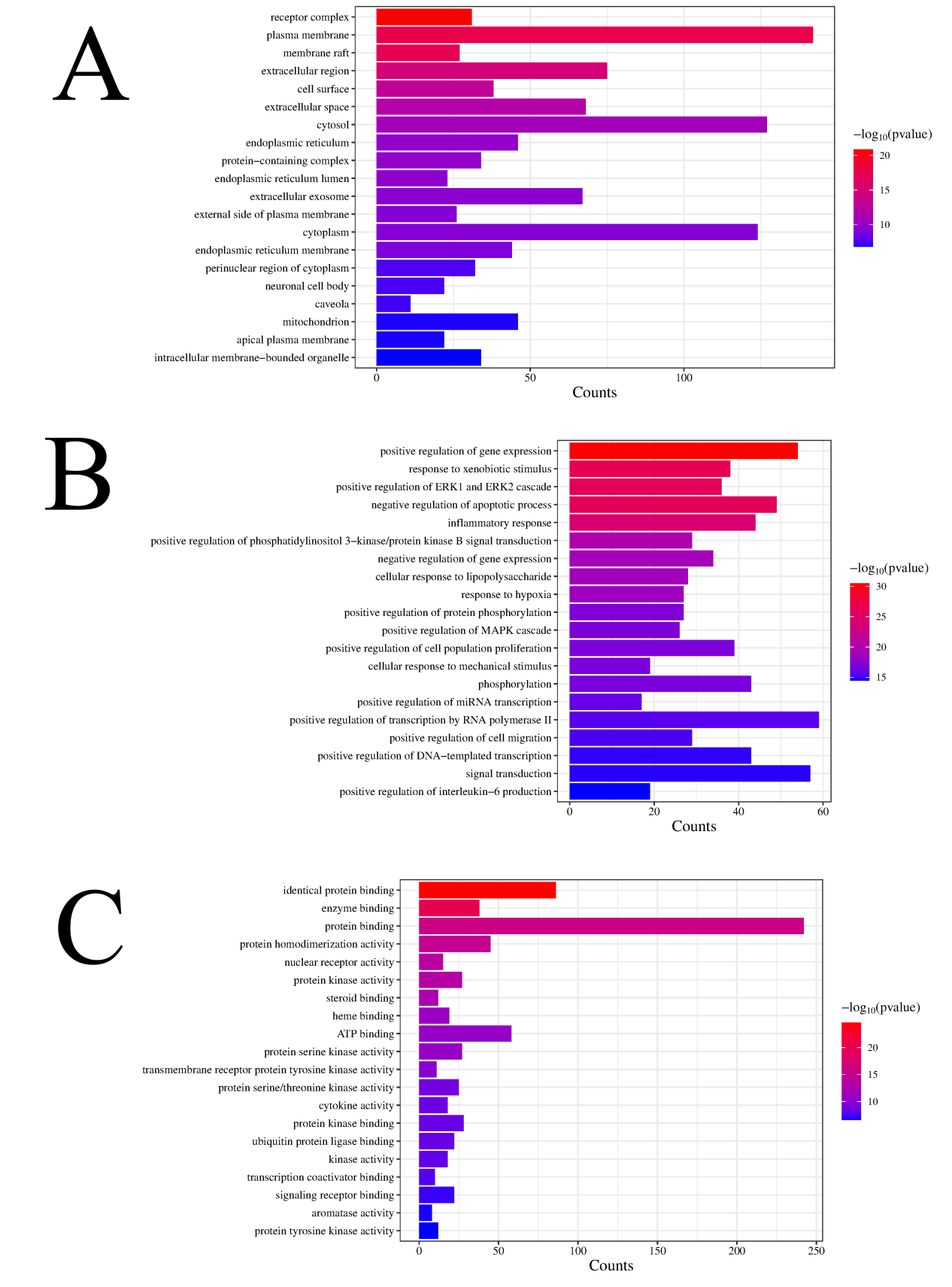


**Figure S2** Network pharmacology analysis of AR against T2DM. (A-C) The top 20 items of GO enrichment analysis of the targets of the bioactive components of AR.

1. **Methodological Investigation and Identification Process of Metabolomics Analysis**

To verify the stability and precision of the analytical method and instrument, we inserted a quality control (QC) sample after every four serum samples during the analysis. Figure S3 shows the PCA score plot of the QC samples. The relative standard deviation of peak areas for all QC samples remained within 2 SDs. Hotelling's T² results confirmed that all QC samples fell within the 95% confidence interval, demonstrating the stability of the UHPLC-MS system and the reliability of the data.


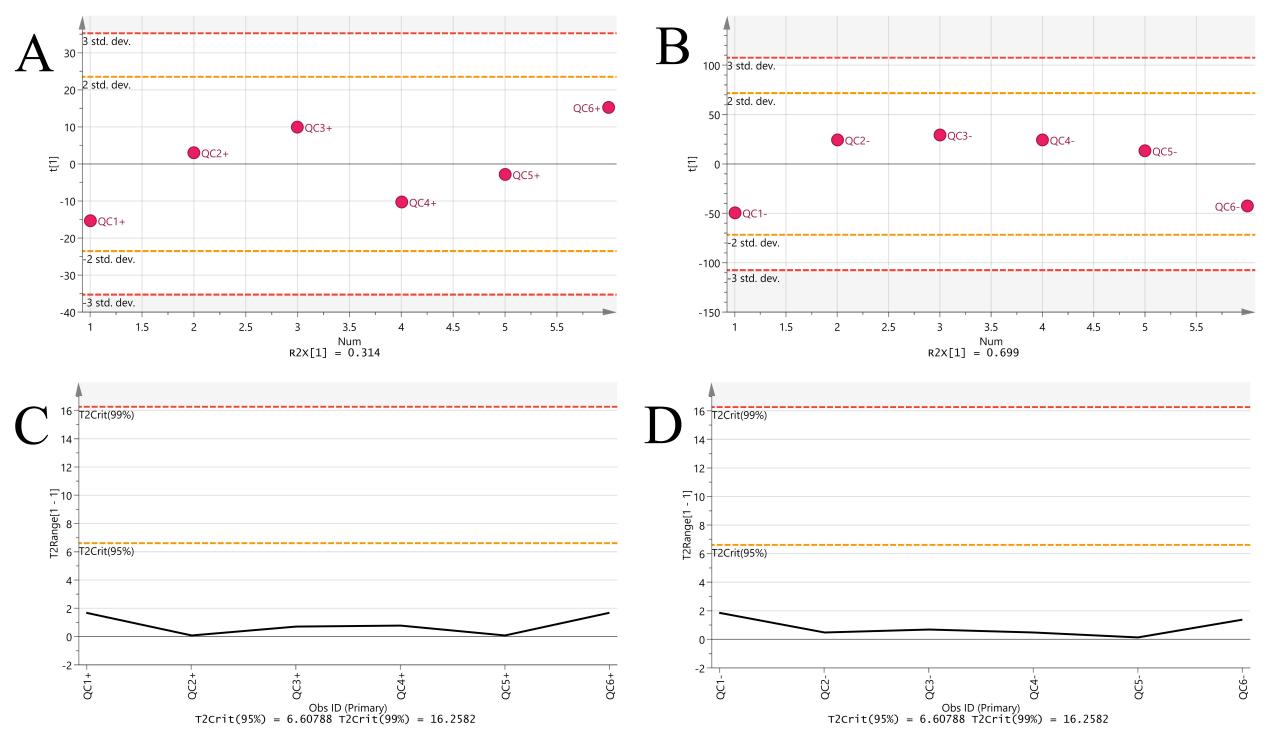


**Figure S3** PCA score plots of serum non-targeted QC samples under positive and negative ion modes and Hotelling's T2 plots (A, C: ESI+, B, D: ESI-). PCA, principal component analysis; QC, quality control.

We analysed the serum samples using unsupervised PCA and supervised OPLS-DA in both positive and negative ion modes, which revealed distinct separation and clustering patterns among the experimental group. Figure S4A-D shows that each group formed clusters to varying degrees in both ion modes. Notably, the normal control group clustered closely with the ROG and ARH groups but remained clearly separated from the DM model group. These results suggest that ARH and ROG treatment effectively ameliorated metabolic disorders in the DM model rats.

To further distinguish the clustering patterns between the DM model group, NC group, and ARH group, and to identify differential metabolites contributing to their separation, we conducted pairwise analyses using the OPLS-DA model. Higher values of the evaluation parameters R^2^(X), R^2^(Y), and Q^2^(cum) indicate a more robust model, particularly when R^2^(Y) and Q^2^(cum) approach 1. R^2^(Y) reflects the model's explanatory power, while Q^2^(cum) represents its predictive capability (detailed parameters in Table S10). As illustrated in Figure S4E-P, most OPLS-DA models showed differences between R^2^(Y) and Q^2^(cum) of < 0.3, with Q^2^(cum) > 50%, demonstrating strong predictive and explanatory performance. Permutation tests (n=200) confirmed model validity, as all Q^2^ fitting curves intersected the negative Y-axis, and regression line intercepts were < 0.05. These results confirm that the OPLS-DA models were reliably constructed, without overfitting, and statistically significant. Finally, we identified differential metabolites through volcano plot analysis (Figure S4Q), selecting variables based on FC value and P value.

**Supplementary Table S10 The OPLS-DA model parameters for the plasma lipid samples from each experimental group of rats were analyzed under positive and negative ion modes**

| Group | principal component  (Predictive component+Orthogonal component) | | R^2^X (cum) | | R^2^Y (cum) | | Q^2^ (cum) | | 200 Permutation test (Q2) | |
| --- | --- | --- | --- | --- | --- | --- | --- | --- | --- | --- |
|  | ESI+ | ESI- | ESI+ | ESI- | ESI+ | ESI- | ESI+ | ESI- | ESI+ | ESI- |
| NC *vs*. DM | 1+2+0 | 1+1+0 | 0.593 | 0.588 | 0.989 | 0.969 | 0.725 | 0.876 | -0.351 | -0.508 |
| DM *vs*. ROG | 1+3+0 | 1+2+0 | 0.658 | 0.663 | 0.998 | 0.978 | 0.756 | 0.793 | -0.233 | -0.357 |
| DM *vs*. ARH | 1+2+0 | 1+1+0 | 0.525 | 0.414 | 0.974 | 0.974 | 0.423 | 0.844 | -0.283 | -0.648 |


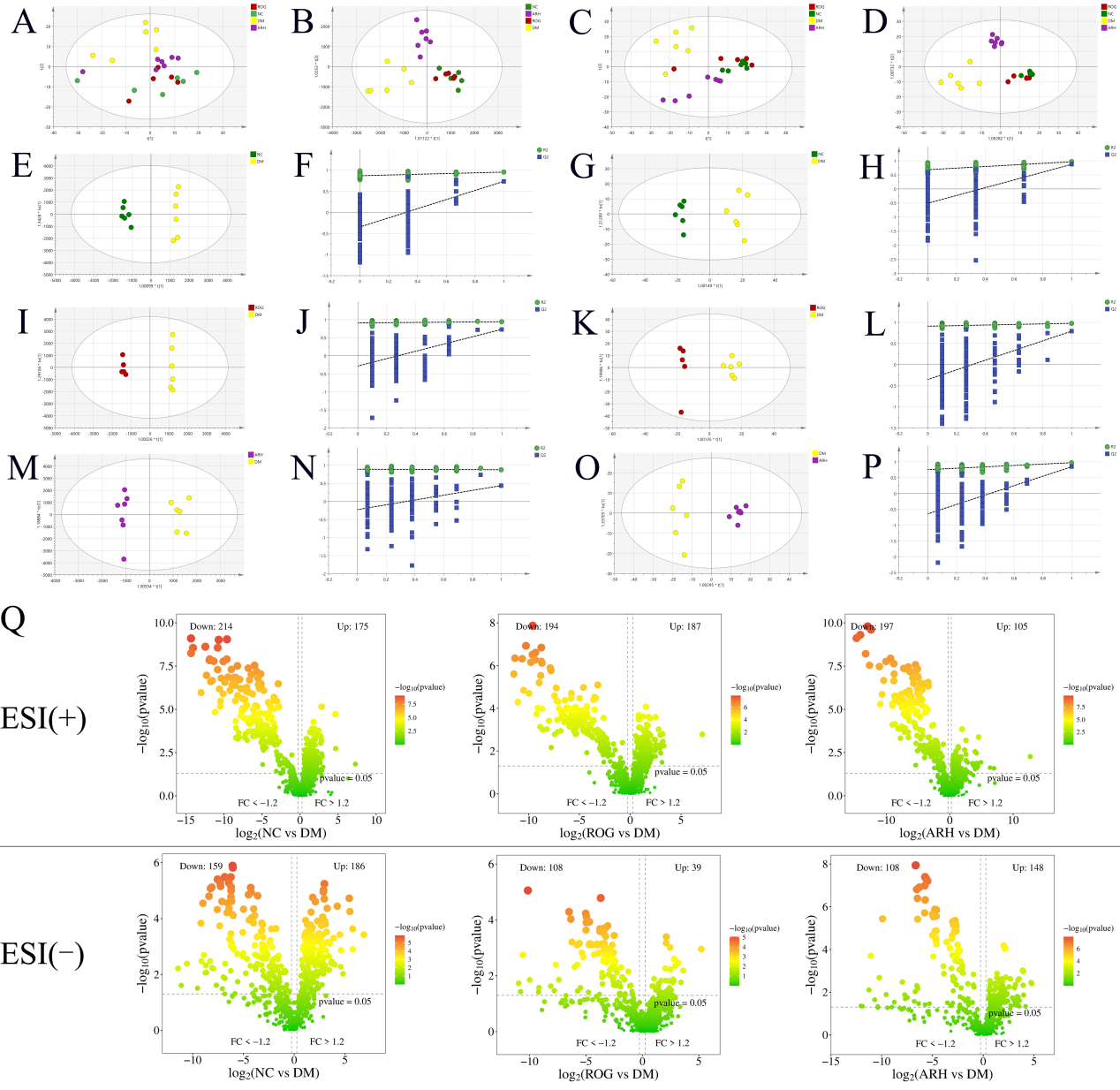
**Figure S4**  Multivariate statistical analyses of serum metabolomics. (A, C) PCA and (B, D) OPLS-DA score plots of the NC (green color), DM (yellow color), ROG (red color), and ARH (purple color) groups in positive (A, B) and negative (C, D) ion modes. (E, G) OPLS-DA score plots of the NC and DM groups in positive (E) and negative (G) ion modes. (I, K) OPLS-DA score plots of the ROG and DM groups in positive (I) and negative (K) ion modes. (M, O) OPLS-DA score plots of the ARH and DM groups in positive (M) and negative (O) ion modes. (F, H, J, L, N, P) 200 Permutation test plots of the OPLS-DA model in both modes. (Q) Volcano plot of differential metabolites among NC, DM, ROG, and ARH groups. PCA, principal component analysis; OPLS-DA, orthogonal projection discriminate analysis; NC, normal control group; DM, diabetes mellitus group; ROG, rosiglitazone group; ARH, high-dose AR group; ARM, medium-dose AR group; ARL, low-dose AR group.

1. **The process of AR components analysis**

The base peak ion (BPI) chromatograms of AR extracts in positive and negative ion modes are shown in Figure S5A-B. The AR extract primarily contained two component types: 8 flavonoids and 69 steroid saponins. Total ion current (TIC) chromatograms of rat serum (collected at 30, 60, and 120 min) and 24-hour urine samples after AR administration appear in Figure S5C-N. We detected 47 prototype constituents from AR extracts (Table S5) and 11 metabolites (Table S6) in rat serum and urine. Figure S6 displays the chemical structures of all 77 AR constituents and their metabolites, while Figures S7-S10 provide detailed mass spectrometric characterisation data for these components.

The AR extract was rapidly absorbed into the bloodstream within 30 minutes of administration, followed by metabolism or elimination. By two hours post-administration, both the quantity and concentration of prototype constituents had declined significantly. We detected 11 metabolites in serum and urine, eight of which were identified as of mangiferin or norathyriol derivatives (Figure S11). Of these, five metabolites appeared in rat serum, while three were unique to urine. These identifications relied primarily on characteristic fragment ions (*m/z* 215, 203, 187, and 107), which are diagnostic of norathyriol fragmentation (Figure S12). The principal metabolic transformations of mangiferin/norathyriol involved glucuronidation, sulfation, and methylation (Figure S13). Additionally, we detected three steroidal sapogenins in rat urine—digitogenin, markogenin/neogitogenin, and sarsasapogenin—with one also present in serum. These sapogenins arise from the sequential cleavage of sugar moieties from steroid saponin.

**
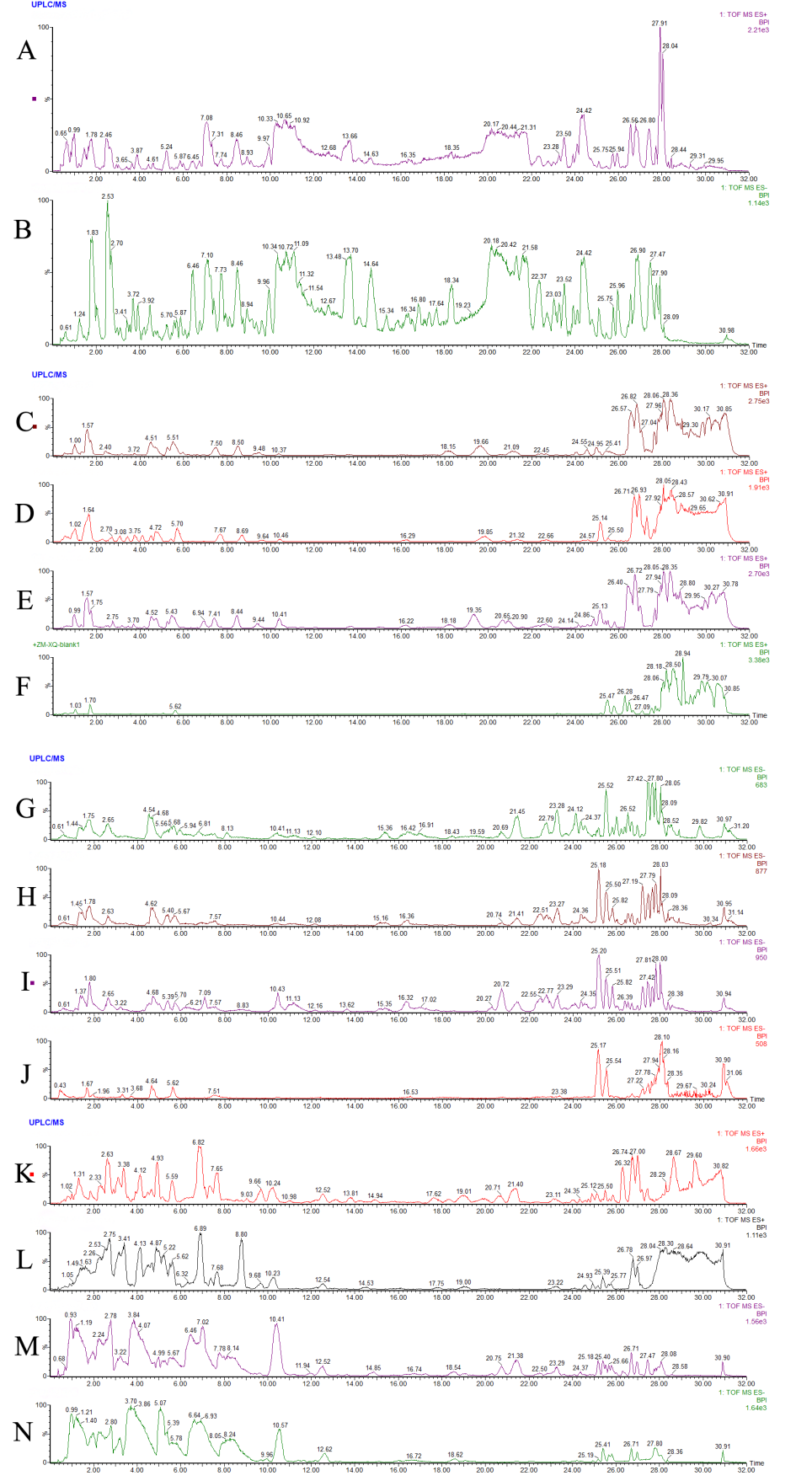
**

**Figure S5** Base peak ion (BPI) chromatograms of AR extracts: Positive ion mode (A) and negative ion mode (B). Total ion current (TIC) chromatograms of rat serum samples at different time points (30, 60, 120 min) post-oral administration of AR: Positive ion mode (C-F) and negative ion mode (G-J). (C, G) 120 min; (D, H) 60 min; (E, I) 30 min; (F, J) 0 min. Total ion current (TIC) chromatograms of rat 24h urine samples post-oral administration of AR: Positive ion mode (K, L) and negative ion mode (M, N). (K, M) AR group; (L, N) NC group.


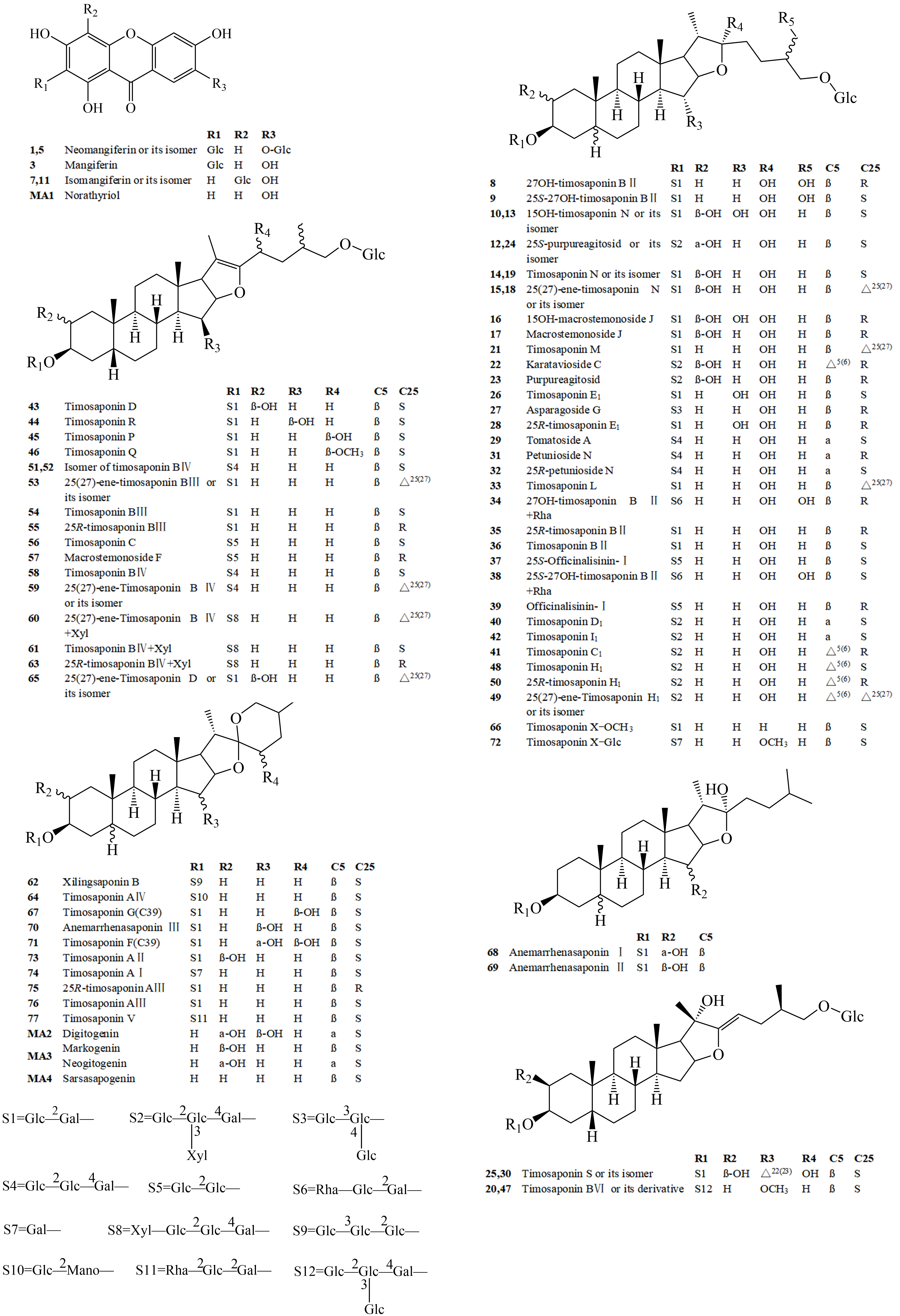
**Figure S6** The chemical structures of 74 constituents in AR and their metabolites in serum and urine. The chemical structures of Peaks 2, 4, and 6 are not listed, and they are dehydration products and dimers of neomangiferin.


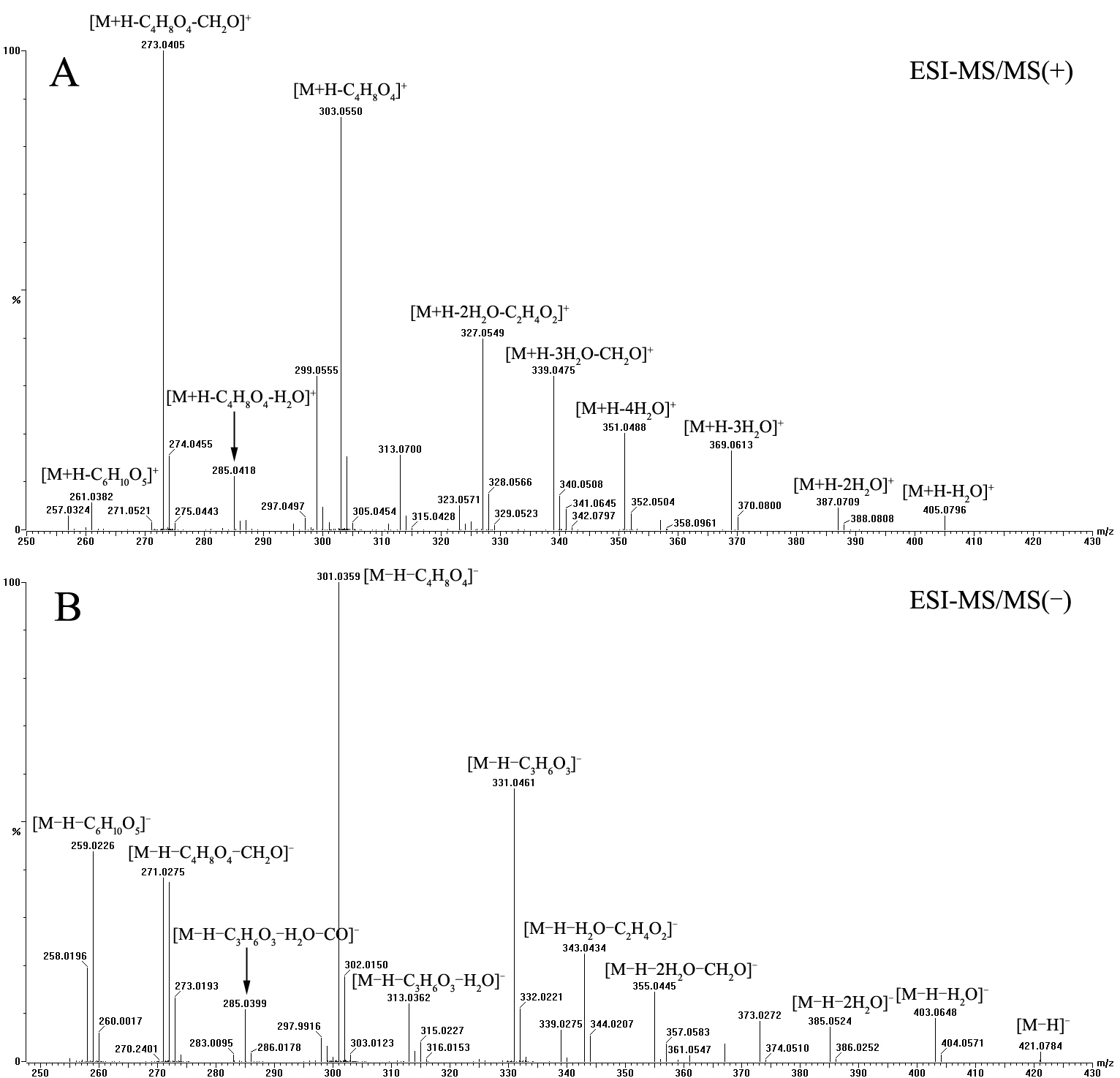


**Figure S7** The MS/MS spectrum of peak 3 in both positive (A) and negative (B) ion modes

*
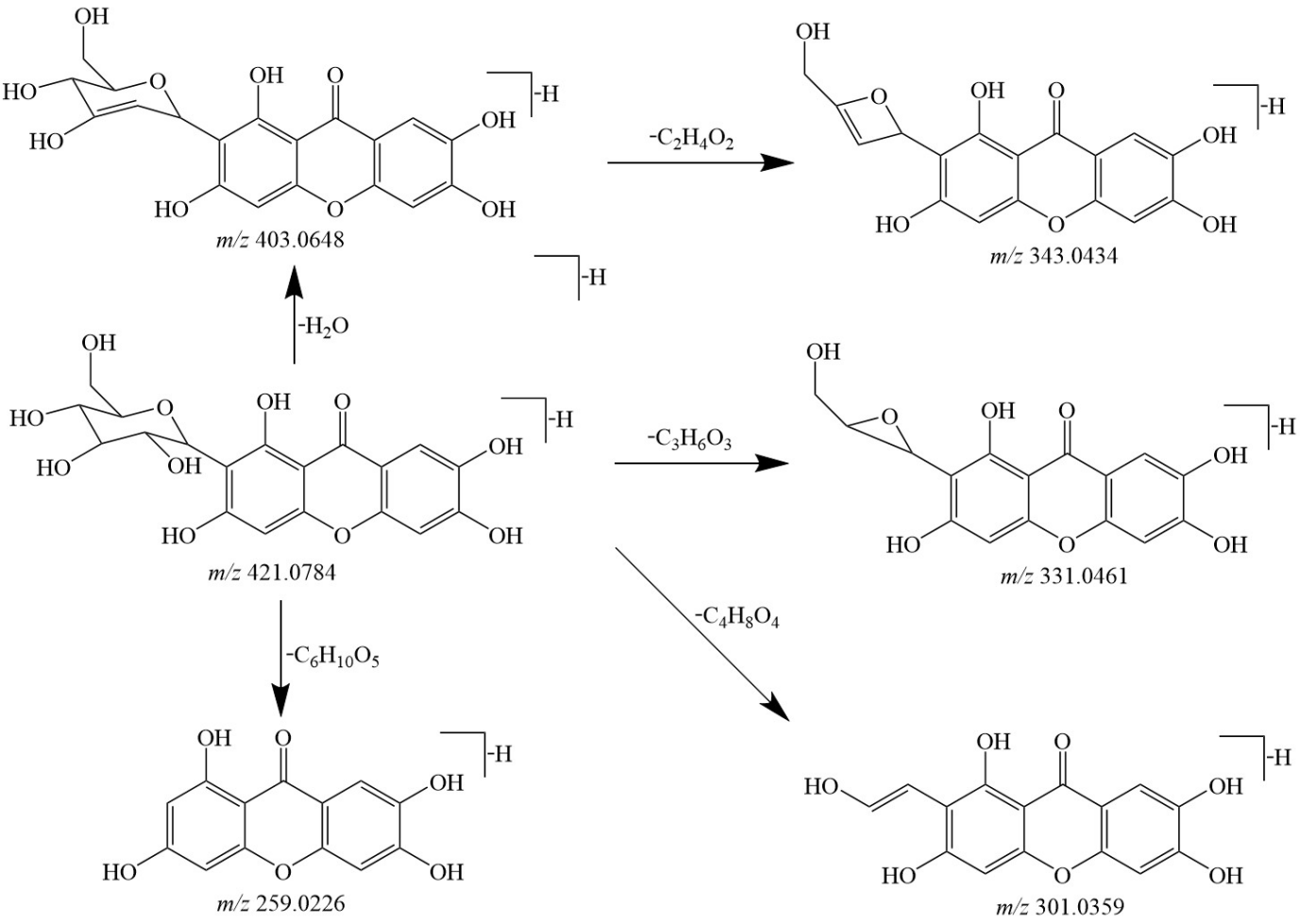
*

**Figure S8** The characteristic fragmentation pathway of peak 3 in negative ion mode


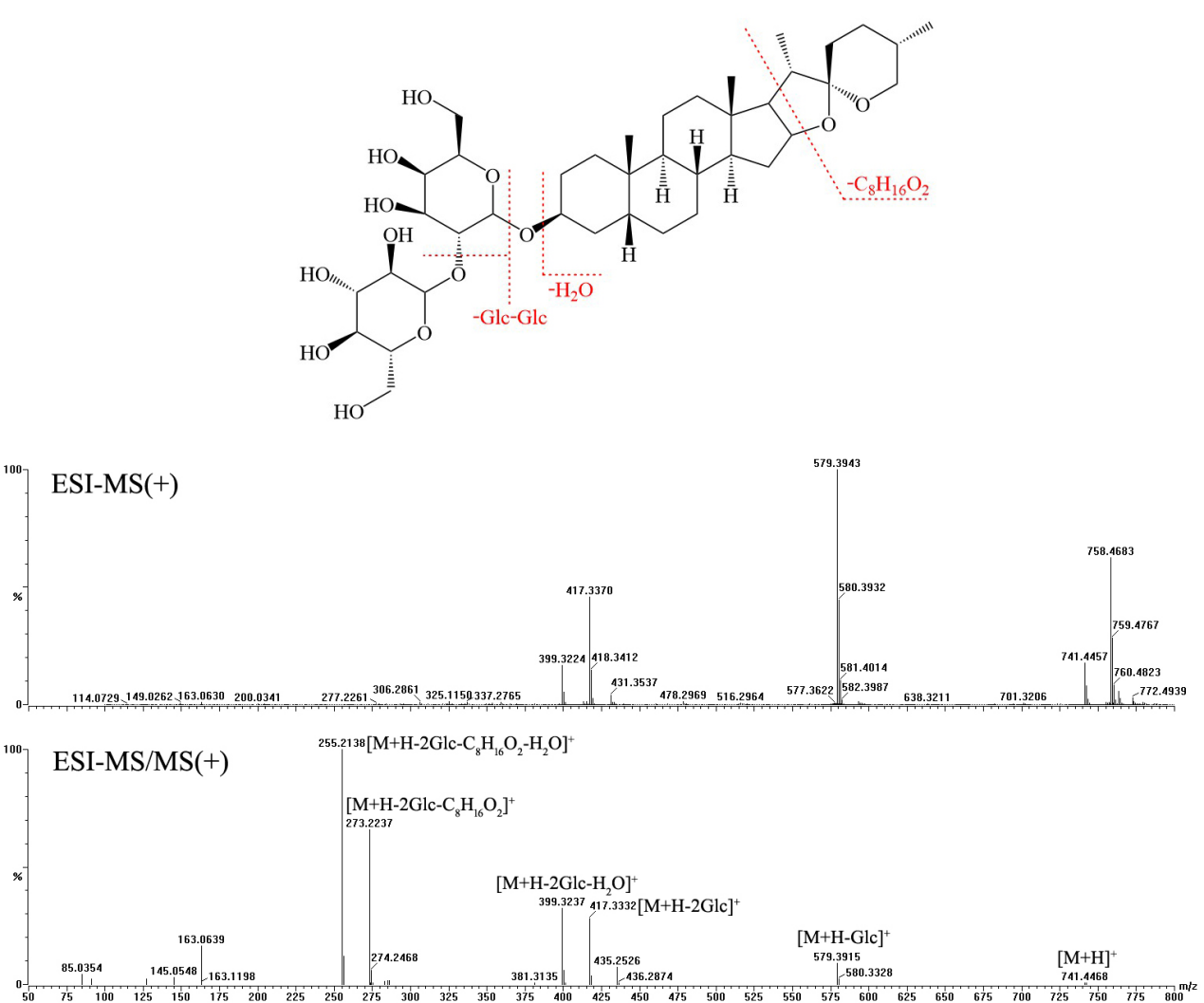


**Figure S9** The MS and MS/MS spectrum of peak 19 in positive ion mode


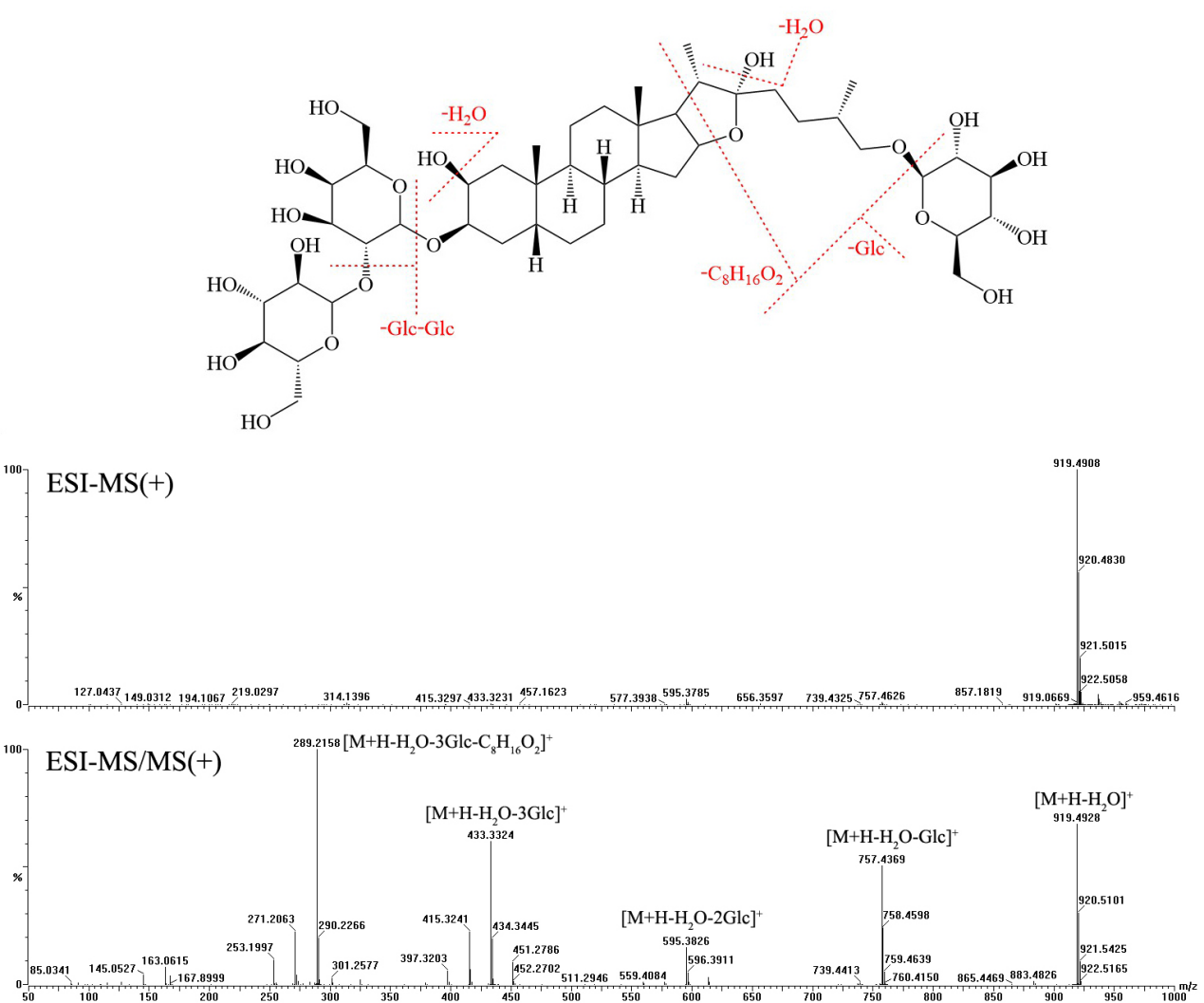


**Figure S10** The MS and MS/MS spectrum of peak 75 in positive ion mode


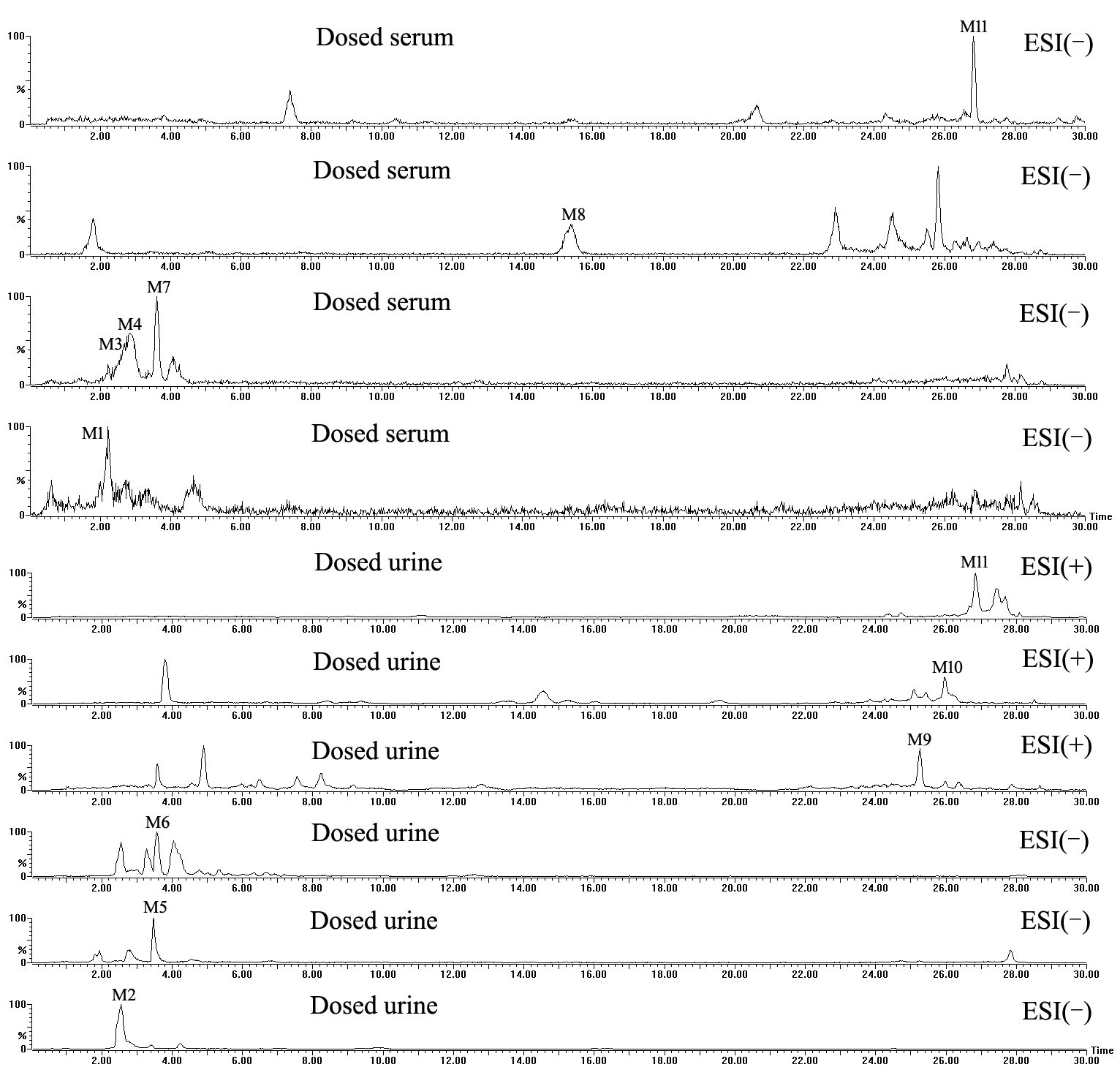


**Figure S11** The extracted ion chromatography of 11 metabolites in serum and urine samples


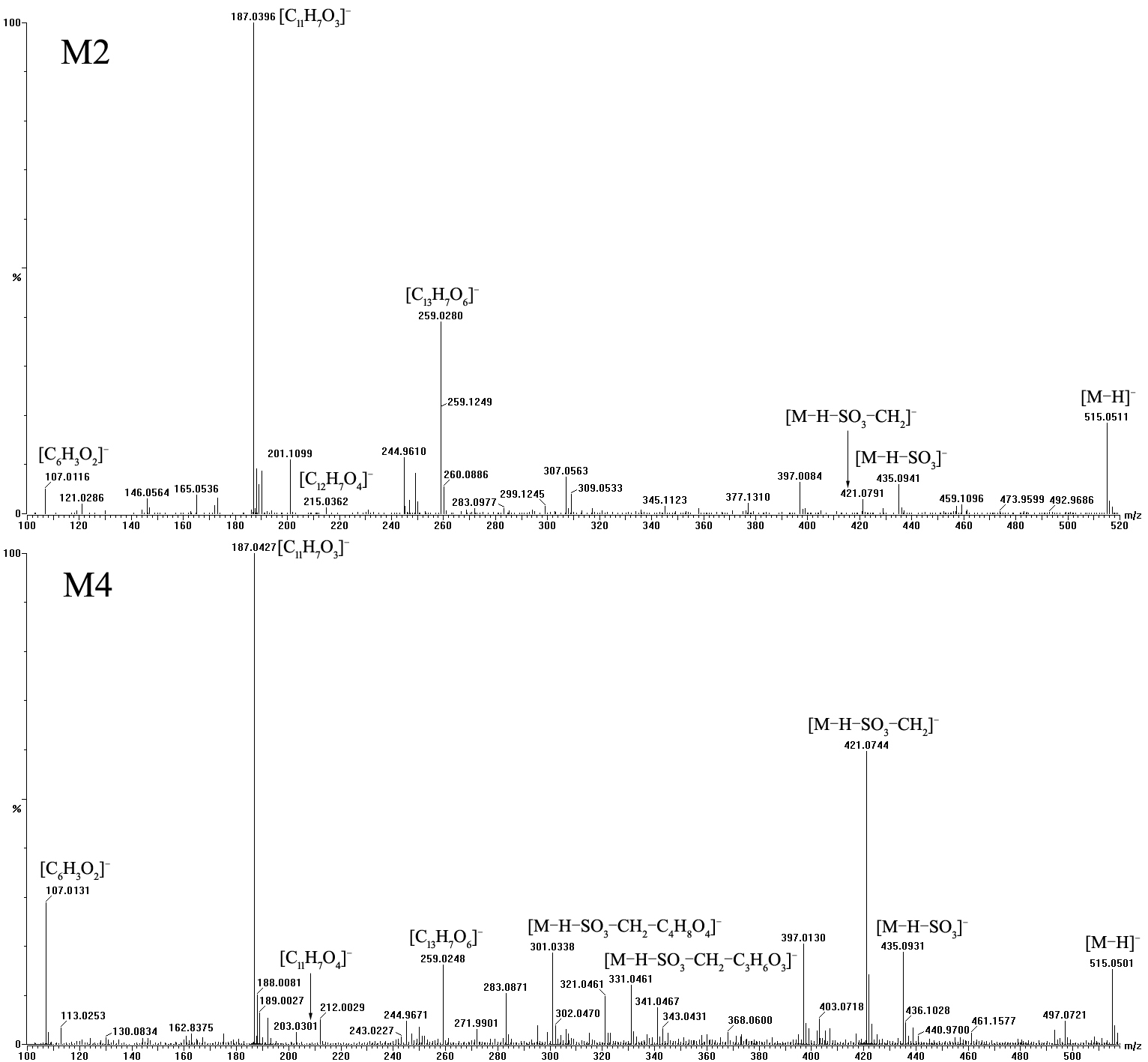


**Figure S12** The MS/MS spectrum of M2 and M4 in negative ion mode

**
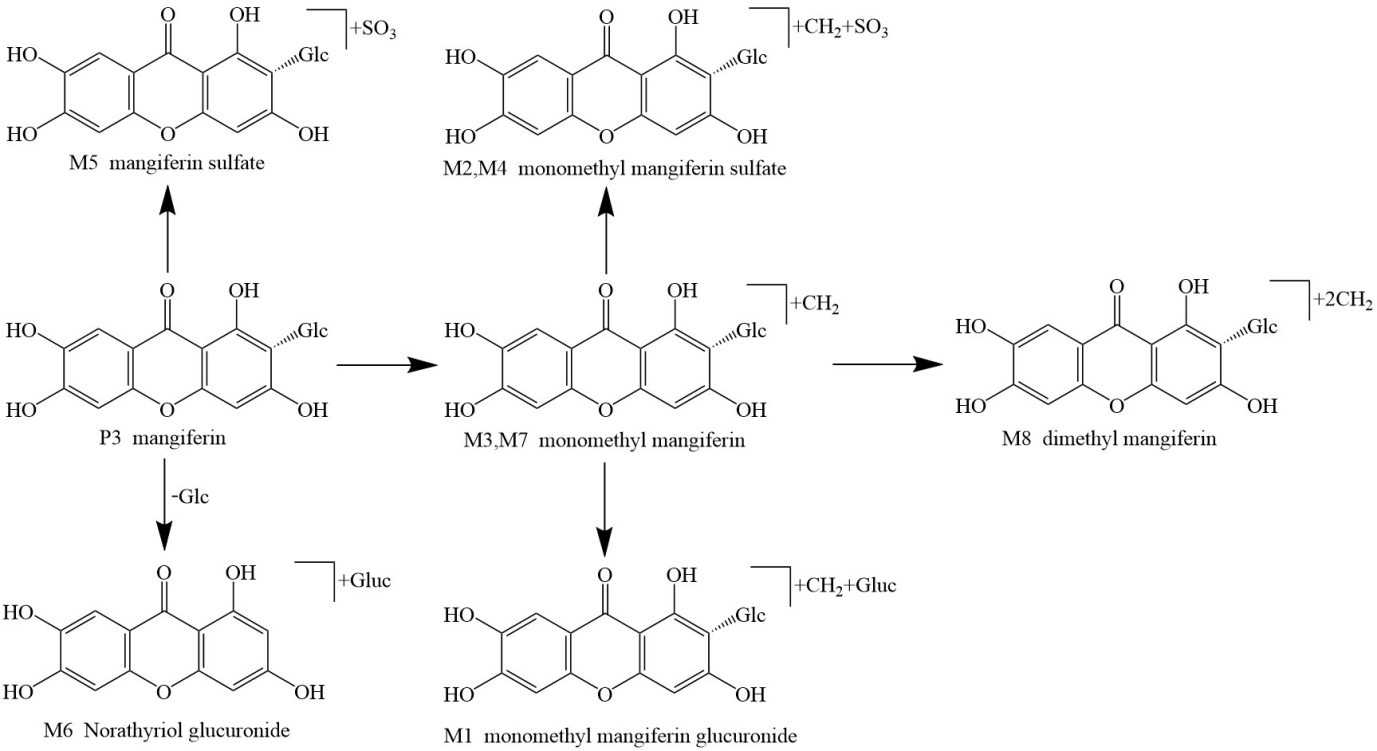
**

**Figure S13** The proposed metabolic pathways of mangiferin or its isomer in rats serum and urine following oral administration
